# European society for organ transplantation clinical practice guideline on prevention and treatment of chronic lung allograft dysfunction

**DOI:** 10.3389/ti.2026.16959

**Published:** 2026-09-18

**Authors:** Saskia Bos, Merel Hellemons, Kavita Dave, Maarten F. M. Engel, Are Holm, Letizia Morlacchi, Veronika Müller, Stefano Pavanello, Clément Picard, Veronica Rossi, Berta Saez-Gimenez, Jens Gottlieb, Robin Vos

**Affiliations:** 1 Department of Respiratory Diseases, University Hospitals Leuven, Leuven, Belgium; 2 Department of Chrometa, KU Leuven, Leuven, Belgium; 3 Erasmus Medical Center Transplant Institute, Erasmus Medical Center, Rotterdam, Netherlands; 4 Department of Respiratory Medicine, Erasmus Medical Center, Rotterdam, Netherlands; 5 Department of Cardiothoracic Transplantation and Mechanical Circulatory Support, Royal Brompton and Harefield Hospitals, Guy’s and St Thomas’ NHS Foundation Trust, London, United Kingdom; 6 Medical Library, Erasmus Medical Center, Rotterdam, Netherlands; 7 Department of Respiratory Medicine, Oslo University Hospital, and Institute of Clinical Medicine, University of Oslo, Oslo, Norway; 8 Respiratory Diseases and Cystic Fibrosis Unit, Medical Area Department, Fondazione IRCCS Ca’ Granda Ospedale Maggiore Policlinico di Milano, Milan, Italy; 9 Physiopathology and Transplantation Department, Università Degli Studi di Milano, Milan, Italy; 10 Department of Pulmonology, Semmelweis University, Budapest, Hungary; 11 European Pulmonary Fibrosis Federation (EU-PPF), Unione Trapianti Polmone Di Padova, Padova, Italy; 12 Respiratory and Lung Transplantation Unit, Foch Hospital, Suresnes, France; 13 Department of Healthcare Professions, Fondazione IRCCS Ca’ Granda Ospedale Maggiore Policlinico, Milan, Italy; 14 Lung Transplant Unit, Respiratory Department, Hospital Universitari Vall D'Hebron, Barcelona, Spain; 15 Department of Respiratory Medicine and Infectious Diseases, Hannover Medical School, Hannover, Germany

**Keywords:** chronic lung allograft dysfunction, prevention, treatment, GRADE, practice guideline

## Abstract

Chronic lung allograft dysfunction (CLAD) is the primary cause of late graft loss after lung transplantation, affecting up to half of lung transplant recipients within 5 years and presenting with heterogeneous phenotypes, most commonly bronchiolitis obliterans syndrome (BOS) and restrictive allograft syndrome. Prevention and treatment of CLAD are challenging. This European Society for Organ Transplantation (ESOT) clinical practice guideline was developed by a multidisciplinary Task Force, including patient and allied healthcare representation, using systematic reviews and GRADE methodology. Eight key PICO questions addressing prevention and treatment of CLAD were evaluated. In terms of prevention, recommendations are in favor of using tacrolimus over cyclosporine, either mycophenolate or azathioprine, and azithromycin to reduce the risk of CLAD. Regarding treatment, montelukast could be considered in early-stage BOS. Current data are inconclusive in demonstrating benefits of anti-thymocyte globulin, alemtuzumab or extracorporeal photopheresis compared with standard of care, and do not support the use of antifibrotics in BOS. The Task Force emphasizes the urgent need for high-quality clinical trials and standardized care pathways in CLAD management.

## Introduction

Chronic lung allograft dysfunction (CLAD) is the leading long-term complication after lung transplantation and remains the major cause of death beyond the first post-transplant year [1]. CLAD is characterized by gradual and irreversible lung function decline, which severely affects quality of life after lung transplantation and may eventually be fatal [2].

CLAD develops in approximately one-third to one-half of lung transplant recipients within 5 years of transplantation and can manifest in different phenotypes [2–4]. The most common are bronchiolitis obliterans syndrome (BOS), characterized by an obstructive spirometric pattern, and restrictive allograft syndrome (RAS), characterized by a restrictive spirometric pattern and radiological opacities. Mixed or undefined forms also occur. The disease course is highly heterogeneous, but mostly progressive over time, necessitating timely intervention to stabilize graft function, alleviate symptoms and prevent graft loss [2, 5].

A range of alloimmune and non-alloimmune factors contribute to CLAD development and progression. Key pathogenic alloimmune drivers include acute cellular rejection, antibody-mediated rejection, human leukocyte antigen (HLA) mismatches, and donor-specific anti-HLA antibodies (DSAs). Non-alloimmune drivers include inflammatory and infectious injuries to the lung allograft, due to ischemia-reperfusion injury, bacterial, viral, or fungal respiratory infections, gastroesophageal reflux with micro-aspiration, and environmental exposures [6].

After lung transplantation, immunosuppressive therapy is used to prevent graft rejection and associated functional decline, graft loss, and mortality, while preserving the best possible quality of life. Standard maintenance immunosuppression includes a calcineurin inhibitor (CNI), a cell-cycle inhibitor and corticosteroids [7]. Although the optimal immunosuppressive regimen remains unknown, practice has changed over time, and the most commonly used combination worldwide is currently tacrolimus, mycophenolate mofetil (MMF), and prednisolone [8–10]. Immunomodulatory drugs, including preventive azithromycin to delay CLAD onset, are used in some centers but are not universally supported or adopted. Other preventive strategies for CLAD focus on reducing modifiable risks and intervening early to preserve graft function. Vigilant monitoring and management of known risk factors, including prompt treatment of acute rejection, pulmonary infections, and gastroesophageal reflux, are considered essential to preserve graft function.

Once CLAD is established, no therapies have been definitively proven to halt or reverse disease progression, and retransplantation remains the only curative option for carefully selected patients. A variety of treatment modalities are suggested and used, ranging from changes in maintenance immunosuppression to lymphocyte depleting and modulatory therapies (e.g., azithromycin, anti-thymocyte globulin (ATG), alemtuzumab, extracorporeal photopheresis (ECP)), as well as other adjunctive therapies (e.g., montelukast) [11, 12]. Most of these drugs are used off-label in the management of CLAD. Considering the limited available evidence and lack of consensus guidelines on therapeutic strategies in CLAD, practice patterns show substantial inter-center variability [10], with no universally accepted standard of care. This clinical practice guideline therefore aims to provide evidence-based recommendations for prevention and treatment strategies for CLAD in lung transplant recipients.

## Methods

This guideline was developed by a European Society for Organ Transplantation (ESOT) Task Force, including specialists in respiratory and transplant medicine, with recognized expertise in the management of CLAD, as well as an allied healthcare specialist and patient representative. The patient representative was actively involved in all discussions as a full member of the panel and provided input into the final recommendation. Between June 2024 and November 2025, the panel met 15 times (all videoconferences and one hybrid meeting). In addition, a smaller methodology subgroup met frequently and regular discussions on individual topics were held via email.

### PICO and narrative questions

Key clinical questions for both clinicians and patients regarding prevention and treatment of new-onset or progressive CLAD were discussed. Following ESOT methodology, we formulated eight questions in accordance with the PICO format (Patient, Intervention, Comparison, Outcomes). Due to the limited number of PICO questions that could be included, it was not possible to address all therapeutic options for CLAD prevention and treatment (e.g., induction immunosuppression, anti-reflux surgery, pulse corticosteroid therapy, total lymphoid irradiation). The final selection of PICO questions was determined based on voting by the Task Force members (Supplementary Material 1).

All Task Force committee members decided on the outcomes of interest for each PICO question, based on their relative importance to adults with CLAD and clinical decision-making [13]. Critical outcomes were graft loss, overall survival and serious adverse events for all PICO questions, as well as CLAD onset for the PICO questions regarding prevention (PICO questions 1–3).

Systematic reviews were performed to answer these PICO questions.

### Disclosure of potential conflicts of interest

Committee members disclosed all potential conflicts of interest prior to the start of the Task Force (Supplementary Material 1). Members with potential conflicts abstained from voting on recommendations in which there was potential conflict. The librarian and medical writer were non-voting members of the Task Force.

### Systematic review

A systematic review was performed according to ESOT methodology. An experienced information specialist (ME) designed and ran the search strategies on the electronic databases of PubMed, EMBASE and Cochrane Library from inception using MeSH terms and keywords for each clinical question. The initial searches undertaken in October 2024 were updated in December 2025. Results of the searches were sent to panel member pairs and the title/abstract and full text were independently screened using predefined inclusion and exclusion criteria. A detailed description of the methods and the PRISMA flow diagrams [14] for all PICO questions are summarized in Supplementary Material 1.

Studies were summarized using the GRADE approach (Grading of Recommendations, Assessment, Development and Evaluation) for guideline development, including both systematic (for PICO questions) and narrative (for additional sources) reviews of the evidence [15–19].

### Assessment of the level of evidence and degree of recommendations

The GRADE approach was used to assess the certainty of evidence and the degree of recommendations [14, 18, 19]. Recommendations were graded as strong or conditional after considering the certainty of evidence, balance between desirable and undesirable outcomes, assumptions about the relative importance of outcomes, implications for resource use, and acceptability and feasibility of implementation. Key considerations correlated with these gradings are summarized in Table 1.

**TABLE 1:** GRADE-based recommendations used in this document, based on GRADE [15] and used in accordance with the European Society for Organ Transplantation methodology.

Target group	Strong recommendation^#^ (“we recommend”)	Conditional recommendation (“We suggest”)
Patients	All or almost all informed people would follow the recommended advice for or against an intervention	Most informed people would choose the recommended course of action, but a substantial number would not
Clinicians	Most patients should receive the recommended course of action	The health professional should acknowledge that different choices may be appropriate for individual patients and should devote time to the process of shared decision‐making by which they ensure that the informed choice reflects individual values and preferences
Policymakers	The recommendation can be adopted as a health policy in most situations	Policy making will require substantial debate and involvement of many stakeholders

^#^ Strong recommendations based on high-quality evidence will apply to most patients for whom these recommendations are made, but they may not apply to all patients in all conditions; no recommendation can take into account all of the unique features of individual patients and clinical circumstances.

Evidence profiles and evidence-to-decision (EtD) frameworks were generated for each PICO question [16] (Supplementary Material 2, 3). Based on these formats, the Task Force Committee formulated clinical recommendations and decided on their strength by consensus or voting, if required. Following the GRADE approach, strong recommendations are phrased as “we recommend”, while conditional recommendations are phrased as “we suggest” [14, 18, 19].

## Results

The number of studies identified and selected for each PICO question is displayed in the PRISMA flow diagrams (Supplementary Material 1). The EtD frameworks for all questions are summarized here, with complete versions in the Supplementary Material, and grouped into prevention and treatment.

### Prevention

**Table:** 

*PICO question 1*
In adult lung transplant recipients, should azithromycin be used to prevent the onset of CLAD?

*Recommendation*
• In adult lung transplant recipients, we suggest using azithromycin to prevent the onset of CLAD (Conditional recommendation in favor of the intervention, very low certainty of evidence)

#### Summary of evidence

Two single-center randomized placebo-controlled trials (RCT) evaluated azithromycin for CLAD prevention (203 patients) [20, 21]. Azithromycin or placebo was administered either from discharge (initiated on average at 36 days post-transplant) for 2 years [16] or preoperatively until 31 days post-transplant [21], respectively. In the latter study, open-label azithromycin was prescribed after 90 days post-transplant and continued in 91% (prior placebo) and 97% (prior azithromycin) of included patients. Long-term outcomes (>2 years) of the first trial were later assessed in a retrospective *post-hoc* analysis (n = 83) [22].

Azithromycin significantly reduced CLAD incidence at 2 years compared with placebo (13% vs. 44%; *p* = 0.017) [20]. At a median follow-up of 5.5 years, azithromycin-treated patients from Vos et al. still showed a lower incidence of CLAD (28% vs. 51%; *p* = 0.048) and significantly longer CLAD-free survival [22].

#### Other supportive evidence

Two additional retrospective single-center studies assessed the effects of azithromycin prophylaxis. One reported increased overall survival (*p* = 0.002) and reduced risk of BOS in patients receiving azithromycin, although not significant (*p* = 0.07) [23]. The other study found that azithromycin prophylaxis, initiated at week 3 post-transplant or later than 3 weeks post-transplant, compared to patients not receiving azithromycin, was associated with a significantly lower risk of CLAD, with the earliest initiation showing the lowest incidence [24].

In summary, data suggest that azithromycin delays and may prevent the onset of CLAD post-transplant; but there is yet no clear evidence from RCTs that prophylactic azithromycin reduces graft loss and improves overall survival, although longer follow-up data are needed for these outcomes. Evidence on optimal initiation time is limited, though early initiation post-transplant may be preferred [24]. It is currently unclear whether the beneficial effects of azithromycin prophylaxis are more pronounced in specific patient populations.

Across the RCTs and *post-hoc* analysis, adverse effects, including serious allergic, cardiac, neurological, gastrointestinal events (requiring treatment discontinuation in 5% in the azithromycin group vs. 0% in the placebo group), and QT interval changes, were comparable between groups or clinically insignificant [20–22]. Similarly, the two retrospective single-center studies found no increased risk of adverse events, renal dysfunction, or malignancy associated with azithromycin [23, 24]. Of note, azithromycin prophylaxis did not affect the bronchial bacterial microbiota of lung transplant recipients [25].

In contrast, clinical experience indicates that gastrointestinal intolerance to azithromycin, related to its gastroprokinetic properties, may necessitate dose reduction or discontinuation. Ototoxicity was not assessed in the included studies, but prior data from patients with chronic lung disease have shown an association between long-term azithromycin use and increased risk of hearing impairment, which physicians should be aware of [26].

Overall, azithromycin is generally well tolerated. However, some adverse effects, such as ototoxicity, have not been systematically studied, and the long-term impact of continuous use remains uncertain.

#### Justification of recommendation

The Task Force supports the use of azithromycin for CLAD prevention, based on evidence demonstrating a clinically meaningful reduction in CLAD incidence and a generally favorable safety profile with mild and infrequent adverse events.

#### Implementation considerations

Oral administration of azithromycin can be initiated during index admission or at routine follow-up visits. No additional monitoring beyond standard post-transplant care is required.

#### Future research

Future research on azithromycin should address the optimal timing of initiation after lung transplantation and whether treatment should be applied universally or targeted to specific risk groups. Studies should also incorporate patient-reported outcome measures and further evaluate long-term effects, including impact on graft and overall survival.

Task Force members had mixed opinions on whether a large multi-center randomized trial of azithromycin to prevent CLAD is needed. Better evidence would be useful, especially on giving the drug to all patients versus only higher-risk ones, and on the best timing. However, a placebo-controlled trial raises ethical and practical problems: the drug is already widely used, recruiting patients would be difficult and the study would need long follow-up. The main uncertainty is therefore not whether azithromycin works overall, but how and when it should be used, including which patients to treat, how long to treat them, and when to stop. Pragmatic randomized trials or large multi-center observational studies may be more realistic ways to answer these questions.

**Table:** 

*PICO question 2*
In adult lung transplant recipients, should tacrolimus be used over cyclosporine to prevent the onset of CLAD?

*Recommendation*
• In adult lung transplant recipients, we recommend using tacrolimus over cyclosporine in the prevention of CLAD. (Strong recommendation in favor of the intervention, low certainty of evidence)

#### Summary of evidence

Four RCTs were identified (662 patients; 329 tacrolimus, 333 cyclosporine), with follow-up duration ranging from 507 days to 3 years [4, 27–29].

Across included trials, tacrolimus was associated with a significantly lower incidence of CLAD with moderate certainty of evidence [4, 27–29]. No statistically significant difference between groups was observed for overall survival (RR 0.91, 95% CI 0.68–1.21, *p* = 0.63–52) [4, 24–26] and graft loss (RR 0.70, 95% CI 0.6–1.06, *p* = 0.10) [4, 27, 29], but studies were not primarily powered for these outcomes.

Serious adverse events were only reported in the ScanCLAD trial [4] and were comparable between groups. Pooled data showed similar rates of infection [4, 27–29]; kidney dysfunction (serum creatinine >2 mg/dL) occurred significantly more often with tacrolimus [27–29] and this was also observed in the ScanCLAD trial [4]. In this study, however, upon switching from extended-release tacrolimus to immediate-release tacrolimus (protocol change during the study), the incidence of kidney dysfunction was no longer higher than with cyclosporine. No significant difference in malignancy was reported with tacrolimus compared to cyclosporine treatment [4, 27, 28].

#### Other supportive evidence

In addition to the RCTs, three quasi-randomized trials [30–32], three observational studies [8, 33, 34] and a recent large registry study [35] were identified.

Reported overall survival was similar [34] or higher [8, 33] with tacrolimus compared to cyclosporine. In the quasi-randomized trials, one-year survival did not differ significantly in two studies [30, 31], while it was higher in the tacrolimus group in the other [27]. CLAD incidence was lower with tacrolimus compared with cyclosporine [31, 32]. The large registry study demonstrated that receiving cyclosporine for maintenance immunosuppression (compared with tacrolimus) was associated with an increased risk of developing CLAD (HR 1.16, 95% CI 1.08–1.23, *p* < 0.001) and with an increased overall risk of death/retransplant (HR 1.16, 95% CI 1.09–1.23, *p* < 0.001).

It should be noted that trough levels varied across studies, and there is currently no consensus on optimal trough targets at different time points after transplantation. The target trough levels for tacrolimus used in the ScanCLAD trial were 10–14 ng/mL at 0–3 months, 8–12 ng/mL at 3–6 months, 8–10 ng/mL at 6–12 months, and 6–8 ng/mL beyond 12 months [4].

Across solid organ transplantation, tacrolimus provides superior early rejection control and short-term graft survival compared with cyclosporine in kidney [36] and liver [37] transplants and is at least non-inferior in heart transplants [38]. However, robust, direct evidence that tacrolimus prevents late chronic allograft dysfunction beyond these surrogate benefits is limited.

No differences in infection rates and kidney dysfunction between tacrolimus and cyclosporine were reported [32, 33]. Tacrolimus was associated with a higher risk of post-transplant diabetes, neurotoxicity and gastrointestinal side effects in liver and kidney transplants [36, 37] but lower risk of hypertension [39].

Conversion from twice-daily, immediate release to once-daily, extended-release tacrolimus formulation appears safe for renal, metabolic, and allograft function and may improve adherence [40], but additional benefits regarding adverse effects remain uncertain. The ongoing Revolution Trial (NCT05001074) compares extended- and immediate-release tacrolimus in lung transplant recipients, assessing renal function and other clinical outcomes [41]. Results expected in 2026 may give insights into the optimal formulation.

#### Justification of recommendation

Data supports the use of maintenance tacrolimus over cyclosporine in reducing CLAD incidence. Mortality and re-transplantation appeared neutral within the limited time frame of most studies, which were not adequately powered to detect a clinically meaningful effect. The registry study, which included a longer follow-up up to 14 years post-transplant, demonstrated an increased overall risk of death/retransplant in cyclosporine-treated patients. Overall certainty of evidence was considered low based on the low certainty of evidence for the critical outcomes graft loss and overall survival, despite moderate certainty of evidence for the outcome CLAD, necessitating recommendation downgrading for risk of bias and imprecision according to GRADE. The moderate certainty of evidence for CLAD is in line with other Refs. [12, 42, 43], but our overall certainty was rated low because of GRADE methodology [44]. However, given the moderate certainty of evidence for the CLAD outcome, the Task Force issued a strong recommendation for the use of tacrolimus even though data on its effect on long-term graft and overall survival are limited.

#### Implementation considerations

There are no major implementation concerns, as both tacrolimus and cyclosporine are widely available and included on the WHO Model List of Essential Medicines [35]. It remains insufficiently clear whether once-daily dosing with extended-release formulation offers advantages over twice-daily dosing with immediate-release formulation in terms of adverse events and side effects. Ongoing and future studies may help clarify this issue. Cyclosporine continues to serve as an essential therapeutic option for patients who experience unacceptable side effects with tacrolimus.

#### Future research

Future research should prioritize long-term patient and graft survival, renal outcomes and adverse effects of different tacrolimus formulations and dosing schedules, and management of severe adverse events. Further study is needed on quality of life, optimal CNI trough levels, therapeutic drug monitoring and dose reduction strategies. Cost-effectiveness of tacrolimus formulations and *versus* cyclosporine could also be important.

**Table:** 

*PICO question 3*
In adult lung transplant recipients, should mycophenolate mofetil be used over azathioprine to prevent the onset of CLAD?

*Recommendation*
• In adult lung transplant recipients, we suggest using either mycophenolate mofetil or azathioprine to prevent the onset of CLAD. (Conditional recommendation for either the intervention or the comparison, low certainty of evidence)

*Remarks*
There may be specific patient subgroups in which selecting one agent over the other is clinically justified. For example, in patients planning pregnancy, azathioprine (AZA) is preferred due to the teratogenic potential of mycophenolate mofetil (MMF). The choice of cell cycle inhibitor may also be influenced by individual susceptibility. AZA may be preferred in patients susceptible to or with persistent gastrointestinal side effects due to mycophenolic acid. Conversely, in patients with or susceptible to myelotoxicity, MMF might be preferred. Similarly, patients with gout requiring treatment with allopurinol (which may cause a serious drug interaction with AZA) may benefit from MMF. Finally, patients with genetic mutations in the thiopurine S-methyltransferase (TPMT) gene should avoid AZA (when homozygous deficient) or require reduced dosing (when heterozygous), as AZA treatment may induce severe, potentially fatal, bone marrow suppression by accumulation of toxic metabolites (6-thioguanine nucleotides) in these patients, which does not occur when treated with MMF

#### Summary of evidence

Two multicenter, randomized prospective open-label studies (399 patients) compared post-lung transplant outcomes between AZA and MMF [45, 46], with just one study reporting CLAD onset [45].

Six-month overall survival rates were similar in one study (86% vs. 82%; *p* = 0.57) [40], while the other study reported an overall three-year patient survival of 75% in the MMF group and 69% in the AZA group (*p* = 0.18) [45]. CLAD incidence at 3 years post-randomization, CLAD grade, time to onset and survival did not differ between groups, but analysis of the composite endpoint (CLAD, death, re-transplantation for graft failure, or withdrawal due to lack of therapeutic response) showed a significant advantage for MMF over AZA, largely driven by a higher rate of treatment withdrawal in the AZA group [45].

With respect to side effects, no significant differences between AZA and MMF were reported in the incidence of CMV infection or disease [46], rates of opportunistic infections, sepsis, abnormal kidney function, or malignancy [45].

#### Other supportive evidence

Several retrospective studies assessed CLAD incidence, yielding inconsistent results [8, 33, 47–51].

One study found a numerically, but not statistically, higher incidence of CLAD in the MMF group (58% MMF vs. 49% AZA) [48]. Three studies reported similar rates of CLAD incidence, graft loss, and survival between MMF and AZA [8, 49, 51]. By contrast, Speich et al. reported a significantly lower graft loss rate and a trend toward better survival in the MMF group; however, interpretation of these findings is limited because of substantial cross-over (42% of patients receiving AZA were switched to MMF) [50].

According to the 2022 consensus on maintenance immunosuppression in solid organ transplantation [7], mycophenolic acid may be more effective than AZA in preventing acute rejection across solid organ transplants, including kidney, pancreas, liver, and heart. Although not a predefined outcome of our guideline, it is worth mentioning that the RCTs found no significant difference in the incidence of acute cellular rejection at 6 months, 1 year and 3 years between MMF and AZA [45, 46]. Conversely, a lower risk with MMF was reported in some retrospective studies [47, 50–52].

Similar adverse events were generally reported across studies, although a trend toward increased gastrointestinal problems with MMF and a higher incidence of anemia with AZA were observed [47, 50]. Rates of infections, malignancy and diabetes were comparable between groups [8, 49]. By contrast, one study reported lower CMV-related disease with MMF [50].

Regarding drug discontinuation, one study found a higher frequency in the MMF group (not significant, *p* = 0.19), mainly due to leukopenia and gastrointestinal issues [46], while another showed higher rates in the AZA group (*p* = 0.026) [50]. Gastrointestinal tolerance may be improved by spreading daily dose throughout the day (*e.g*., three times daily instead of twice daily) or switching to enteric-coated mycophenolate sodium, which delays release of mycophenolic acid until the small intestine. The latter has been associated with improved gastrointestinal symptoms, health-related quality of life, and psychological wellbeing in case series of solid organ transplant recipients, and may limit dose reductions, interruptions or discontinuation [53, 54].

It should be noted that a higher incidence of cutaneous squamous cell carcinoma has been observed in solid organ transplant recipients exposed to AZA [55].

Switching drugs (*e.g*., MMF to AZA for fertility reasons) or omitting cell cycle inhibitors for a longer time may increase the risk of developing *de novo* DSAs and acute rejection [56].

Finally, MMF warrants caution in patients (both females and males) planning pregnancy due to its teratogenic potential [57].

#### Justification of recommendation

Evidence shows no significant difference in effectiveness between AZA and MMF, and both have acceptable safety profiles. Accordingly, the Task Force does not suggest the use of one agent over the other. Overall, there has been an increased utilization of MMF over time. The Task Force additionally reported a modest clinical preference for MMF, primarily attributed to its perceived lower risk of acute cellular and antibody-mediated rejection [7].

#### Implementation considerations

MMF is considered easy to manage in clinical practice, although, unlike AZA, it is not included in the 2025 WHO Model List of Essential Medicines [58]. Regarding individual susceptibility, the U.S. Food and Drug Administration advise thiopurine S-methyltransferase (TPMT) genotyping/phenotyping prior to commencing AZA, due to the risk of severe myelotoxicity in TPMT-deficient individuals [59]. MMF effectiveness and side effects are influenced by polymorphisms in UDP-glucuronosyltransferases enzymes, which regulate bioavailability of its active metabolite; routine pharmacogenetic testing is not yet standard but may guide individualized MMF dosing [60]. Prescribers should be aware that cyclosporine can significantly lower MMF blood levels, necessitating higher doses to achieve the same area under the curve dose level, by inhibiting its enterohepatic recirculation. Finally, caution is advised with both AZA and MMF in patients with documented short telomere syndrome due to increased myelotoxicity, which may necessitate a dose reduction or discontinuation of the cell cycle inhibitor.

Out of the scope of this systematic review is the role of proliferation signal inhibitors, which may represent an alternative in patients intolerant to both MMF and AZA.

#### Future research

Long-term real-world data may complement RCTs by comparing cell cycle inhibitors in lung transplantation. Key unresolved questions include DSA development with MMF vs. AZA, optimal and individualized dosing, identification of patient subgroups that may benefit preferentially from one agent, long-term drug effects, management of MMF during infections, and guidance on treatment interruption around vaccinations.

### Treatment

**Table:** 

*PICO question 4*
In adults with (progressive) CLAD, should extracorporeal photopheresis be used?

*Recommendation*
• In adults with (progressive) CLAD, we suggest either using extracorporeal photopheresis or not using it. (Conditional recommendation for either the intervention or the comparison, very low certainty of evidence)

*Remarks*
There may be specific patient populations that respond preferentially to ECP (e.g., BOS patients, non-rapid decliners, and/or patients with early CLAD stages), but responder profiles need to be consolidated by more data

#### Summary of evidence

Currently, there are no published RCTs on the use of ECP in CLAD. We identified seven cohort studies with a control group assessing 182 CLAD patients treated with ECP compared to 364 controls [61–67]. All studies suffered from significant selection or indication bias; the ECP group often included patients with more advanced CLAD stages or rapid decliners. Treatment protocols varied with different numbers of cycles per month and in total.

Concerning critical outcomes, one study found a higher re-transplantation rate in the ECP group compared with controls (35% vs. 15%; *p* = 0.004) [63]; of note, in this study, the ECP group included more patients with higher CLAD stages and more rapid decliners than the control group. Two studies showed 31%–55% graft loss during follow-up between 12 and 36 months in both ECP and control groups [62, 67]. Four studies found no difference in overall survival compared with controls [61, 62, 65, 67], whereas the study by Pecoraro et al. documented significantly higher survival in the ECP group (155 vs. 114 months; *p* = 0.03) [66]. In Jaksch et al., survival benefit for ECP was only found in responders, not in the overall ECP group, compared to controls [63].

Different methods were used to measure the effect on forced expiratory volume in one second (FEV1) evolution. One study reported an overall positive effect on FEV1 of ECP compared to controls [66], while two studies found no differences [64, 65]. Greater FEV1 decline in the ECP group was observed in two studies 61,63], and in one study assessment was not possible due to a high rate of early mortality in the ECP group [62].

Response rate—defined as FEV1 stabilization and/or improvement—ranged from 33% to 80%. This variation likely reflects differences in patient populations or CLAD stages. For instance, Del Fante et al. found 60% stable ECP vs. 70% stable controls, but with less CLAD stage I patients in the ECP group; over time, overall treatment failure occurred in 67% of patients in the ECP group compared with 93% of controls [61].

Regarding undesirable effects, no difference in infection rates between groups was reported in three studies [63, 65, 66], but one study observed a significantly lower incidence of CMV infections in the ECP group [63]. A decrease in bacterial and CMV infections after ECP initiation compared with the pre-ECP period has also been reported, despite lack of direct comparison to the control group [61]. Serious complications associated with ECP were infrequent across studies.

#### Other supportive evidence

Thirteen additional observational studies evaluating ECP in CLAD were identified, with sample sizes ranging from 8 to 631 patients and some patient overlap among larger cohorts [68–80].

Survival outcomes varied widely. The largest study (631 patients) reported overall survival of 86% at 6 months and 77% at 12 months, with 5-year post-ECP graft survival ranging from 70% in responders, to 56% in stable patients and 35% in non-responders [79], while another large cohort (373 patients) reported 46% overall survival and 41% graft survival at a median follow-up of 92 months [73]. Other studies reported a median overall survival of 7 years, with 50% 10-year graft survival [71], 66% 5-year overall survival [75], or median survival ranging from 2.6 years post-ECP initiation [72] to a mean of 15 months [80].

Most studies focused on graft function, particularly FEV1 evolution before and after ECP initiation. In the largest cohort [79], FEV1 stabilization or improvement was observed in 62% of patients at 6 months and 51% at 12 months, while other studies reported response rates of 19%–80% [68–70, 72, 74, 79, 80]. Interestingly, a recent randomized trial evaluated the preventive use of ECP in the early post-operative course and found a significantly lower incidence of CLAD at year 3 in the ECP group [81].

Several subgroup analyses attempted to identify responder profiles. Patients with a BOS phenotype generally demonstrated better responses than those with a RAS phenotype [65, 74, 79], although findings were inconsistent [61]. Similarly, patients with earlier CLAD stages (I–II) tended to respond better than those with advanced disease (III–IV) [63, 73, 75, 79], although this association was not uniformly observed [61, 66, 74]. Rapid FEV1 decliners showed reduced or trending toward reduced responses in some studies [63, 74], while others reported no effect [64] or even better responses [77].

Adverse effects were generally infrequent. Since ECP is thought to have mainly immunomodulatory rather than immunosuppressive effects, it may cause fewer infections [79] compared to other treatments. Treatable line-related infections occurred in 13%–14% of patients requiring central venous access [69, 72]. Venous access is a notable concern and may pose a significant barrier to treatment with ECP; based on our experience, the use of central lines varies considerably across centers. No ECP-specific long-term toxicities were reported, with long-term follow-up studies showing reassuring safety profiles [71, 75]. Fatigue and iron deficiency anemia, though underreported, are observed in clinical practice and warrant monitoring. In a RCT where ECP was used as a preventive immunomodulatory treatment to reduce the risk of CLAD, the main adverse event attributable to ECP was anemia, reported as a reason for discontinuation in 38% [81].

In conclusion, the actual efficacy of ECP in the treatment of CLAD remains uncertain. Multiple sources of bias were identified across studies, including logistical and co-intervention bias, and the lack of adequately matched control groups limited the ability to distinguish treatment effects from the natural history of CLAD. Results from the ongoing randomized eCLAD trial (UK, NCT05721079) are awaited to provide more robust evidence [82].

Quality of life and patient-reported outcome measures were not assessed in the published studies, representing an important gap. Given the burden of long-term, repeated ECP sessions, patient engagement, counseling, and stopping protocols are essential.

#### Justification of recommendation

Although ECP shows few severe side effects, the limited and not well-established desirable effects, along with the significant therapeutic and logistical burden for patients and hospitals, preclude the group from making a recommendation either for or against the use of ECP in the management of CLAD. This recommendation may require revision when additional data become available, especially following the results of the ongoing multicenter randomized controlled eCLAD trial.

#### Implementation considerations

High procedure costs (see Table 2), requirement for specialized equipment and trained personnel at hospitals, potential need for a central line, and coordination of travel logistics for the patients should be considered when assessing ECP indication. Reimbursement policies across healthcare systems are variable.

**TABLE 2:** Dosing and costs of various CLAD treatments.

Treatment	Typical CLAD regimen	Costs*	Notes
Azithromycin	250 mg orally 3×/week or 250 mg every other day	€	Generic; very low cost across EU. Often <€10 for a month’s supply
Montelukast	10 mg orally daily	€	Generic; inexpensive in most EU countries
Pirfenidone	801 mg 3×/day (standard dose)	€€	Generic; widely available. Actual reimbursed costs vary
ECP	Initial: 2 days every 1–2 weeks; Maintenance: 2 days every 4 weeks**	€€€	Varying availability. Highly variable by center, frequency, and technology (online vs. offline). Reimbursement differs by country
ATG	Short course (3–10 doses, total 3–9 mg/kg)	€€€	Varying availability. Vial costs vary; total depends on weight and protocol
Alemtuzumab	Usually 1–few 10–30 mg IV doses	€€€	Varying availability

* Monthly costs in Europe € - < 100€, €€ 100–1.000€, €€€ = >1.000€.

** Differing schedules exist.

ATG, anti-thymocyte globulin; CLAD, chronic lung allograft dysfunction; ECP, extracorporeal photopheresis.

#### Future research

Further research on ECP in established CLAD should clarify treatment response, define responder subgroups, standardize treatment schedules, and assess biological samples to elucidate its mechanism of action, patient-reported outcomes, side effects, and cost-effectiveness. Additional studies are also needed to evaluate the role of ECP in CLAD prevention.

**Table:** 

*PICO question 5*
In adults with (progressive) CLAD, should anti-thymocyte globulin be used?

*Recommendation*
• In adults with (progressive) CLAD, we suggest either using anti-thymocyte globulin or not using it. (Conditional recommendation for either the intervention or the comparison, very low certainty of evidence)

*Remarks*
We suggest using ATG with caution in patients with a history of or risk for malignancy (*e.g*., recent malignancy, malignancy with a high risk of recurrence, Epstein-Barr virus (EBV) mismatch), or a history of infections (especially difficult-to-treat or fungal infections)

#### Summary of evidence

We identified nine retrospective observational studies assessing ATG as a treatment for CLAD (390 treated patients, 10–108 per study) [83–91].

Graft survival ranged from 77% to 80% at a median follow-up of 10–63 months [84, 88]; in one study with an ATG-free control group, median graft survival was lower in ATG-treated patients (26 vs. 45 months; *p* = 0.021), possibly reflecting more severe baseline disease and residual confounding despite propensity matching [90]. Overall survival was 95% at 6 months [87], 90% at 10 months [84], 54% at 12 months [89] and 56% at 17 months [86]. The study by Padhye et al. showed a lower 1-year post-CLAD mortality compared with CLAD patients who did not receive ATG, although not significant (SHR 0.66, 95%CI 0.39–1.14, *p* = 0.134) [91].

Across studies, FEV1 response rates varied depending on the definition applied. Improvement in FEV1 was observed in 13%–40% of patients, while improvement or stabilization was reported in 23%–52% [83, 85, 86, 88, 89]. A >20% reduction in FEV1 decline occurred in 40%–65% of cases [87, 90]. Padhye et al. reported no difference in rate of FEV1 decline post- vs. pre-ATG and compared with non-ATG treated patients, although no pre-defined lung function period was assessed; *i.e*., the rate of decline 3 months after CLAD diagnosis to last follow-up was compared with lung function evolution from transplant up to 3 months before CLAD diagnosis [91].

Reported undesirable effects included skin cancer (9%–19%) and other types of malignancy (7%–8%) [88, 90]. Post-transplant lymphoproliferative disorder (PTLD) occurred in 0%–10% of cases [83, 84].

#### Other supportive evidence

The rate of FEV1 decline as a predictor of ATG response yielded inconsistent results. A trend toward a better response in rapid decliners [90] and CLAD stage I-II patients [88, 89] was observed, although this association was not consistently reported [87, 90]. No association between response and CLAD phenotype was reported in three studies [87, 88, 90].

There was wide variability across studies in terms of follow-up and time between CLAD diagnosis and ATG treatment. Studies also differed in total dose and type of ATG administered, as well as monitoring of lymphopenia. ATG was generally used as a second- or third-line therapy, with prior treatments varying across studies.

The Task Force judged that the evidence for a positive effect was limited, mostly because most studies lacked control groups. However, clinical experience suggests that ATG may contribute to lung function stabilization in some cases.

Treatment tolerance varied across studies. Fever and chills were common [84, 86, 87, 89], while most adverse events were mild [84, 89]. Infusion-related reactions ranged from 4% [86] to 15% cytokine release syndrome and 22% serum sickness [87]. Overall, severe reactions appeared rare, and premedication with corticosteroids, acetaminophen, and/or antihistamines may reduce symptoms.

Bacterial and/or viral infection rates ranged from 12% to 35%, with serious infections occurring in 8%–14% of patients [88, 89]. Cytopenia was inconsistently reported: severe leukopenia occurred in 4% of patients [86] and neutropenia in up to 14% [89].

Additional safety information can be derived from ATG use as an induction therapy. A systematic review of six small RCTs found no clear evidence of a higher risk of adverse outcomes associated with T-cell antibody induction (including ATG) compared with no induction or with each other, in lung transplantation [92].

Although ATG increases overall immunosuppressive burden, retrospective studies showed no clear increase in the risk of PTLD in lung or other solid organ transplantation [93]. Current expert opinion favors use of the lowest effective dose, with caution in EBV-seronegative recipients and other high-risk populations.

#### Justification of recommendation

There is no high-quality data supporting the use of ATG in the management of CLAD, although existing studies and Task Force members’ clinical experience suggest potential for lung function stabilization. The main limitation of ATG therapy is an increased risk of infections. Overall, ATG may be considered as a treatment option in selected CLAD patients, including those with concomitant ACR.

#### Implementation considerations

Availability of this therapy may be limited in certain countries, and treatment necessitates hospitalization and intravenous access. Administration of ATG via a central line or large-bore intravenous access is generally preferred given the risk of local irritation and thrombophlebitis. At present, no definitive recommendations exist on the optimal dosing, duration of treatment, and monitoring of its effects.

#### Future research

RCTs are warranted to better define efficacy and safety. Additional evidence is required to determine optimal dosing, treatment duration, and appropriate monitoring strategies. Furthermore, data is lacking for high-risk groups, such as EBV-mismatched recipients at increased PTLD risk, and for specific clinical subgroups, including different CLAD phenotypes.

**Table:** 

*PICO question 6*
In adults with (progressive) CLAD, should alemtuzumab be used?

*Recommendation*
• In adults with (progressive) CLAD, we suggest either using alemtuzumab or not using it. (Conditional recommendation for either the intervention or the comparison, very low certainty of evidence)

*Remarks*
Alemtuzumab should be used with caution in patients with a history of or risk for malignancy (*e.g*., recent cancer, malignancy with a high risk of recurrence, EBV mismatch) or a history of infections, particularly difficult-to-treat or fungal infections

#### Summary of evidence

Three small retrospective observational studies were identified (41 patients; 10–17 per study) [65, 94, 95], including one with a control group of 78 untreated patients [65].

No significant difference in overall survival was observed between the treatment group and controls at 6–12 months [65]. The other studies reported graft and patient survival of 69% at 1 and 2 years [94] and 5-year overall survival of 76% [95].

In one study, mean FEV1 did not improve following alemtuzumab treatment [94]. In another cohort, a >10% increase in FEV1 was observed at 30 days, followed by a 10% decline by day 120 [95]. In the controlled study, alemtuzumab was associated with a slower decline in FEV1 at 1 month, but not at later time points [65]. Reams et al. observed improvement in BOS grade in 40% of patients, stability in 30%, and worsening in 30% [94]. Ensor et al. reported 53% freedom from BOS progression at 6 months [95].

Overall, alemtuzumab was associated with short-term attenuation of spirometric decline, mainly in BOS and early-stage disease patients, but no survival benefit was shown, and effects beyond 3–6 months remain unclear [65, 94, 95]. Interpretation of the results is limited by selection bias, as alemtuzumab was preferentially used for rapidly progressive disease. Further data is needed to determine the true effect size. Nonetheless, experience from some Task Force centers suggests possible stabilization of lung function, and alemtuzumab may be a potential option in patients with prior DSAs.

#### Other supportive evidence

In the identified studies, infection rates ranged from 50% to 77% while infection-related mortality ranged from 10% to 30% [65, 94, 95]. Patients with CLAD already have an increased baseline infection risk; alemtuzumab may further increase the incidence.

In kidney transplant studies, alemtuzumab or ATG treatment for acute T-cell–mediated or glucocorticoid-resistant rejection was associated with good long-term graft survival and function, but frequent infections and reduced patient survival were reported compared with patients not requiring alemtuzumab [96–98]. Infection rates were comparable between the alemtuzumab- and ATG-treated groups [97].

In a study of alemtuzumab induction therapy in lung transplantation, 44% of patients developed (mostly viral) infections under prophylaxis (27% hospitalized, 4% ICU), nearly 50% occurring within 6 months. Malignancies occurred in 9% of patients (2% PTLD, 7% non-PTLD) [99].

Although data does not demonstrate a clear increase in post-transplant malignancies with alemtuzumab in kidney or lung recipients, clinical experience supports cautious use in patients with a prior history of cancer.

#### Justification of recommendation

Alemtuzumab may stabilize lung function in selected patients, but its effect size remains uncertain, and further data is warranted. Its use is supported by limited clinical experience, and potential infection risks should be carefully weighed in clinical decision-making.

#### Implementation considerations

Alemtuzumab may have limited availability in some regions, potentially affecting access. It is generally administered intravenously, though subcutaneous or intramuscular routes are alternative options. The costs of the drug and management of potential adverse events should be carefully evaluated during treatment planning.

#### Future research

RCTs are necessary to establish definitive evidence regarding the efficacy, safety, optimal dosing and scheduling of alemtuzumab. Future research should additionally assess risk factors for adverse events (*e.g.,* PTLD risk in patients with EBV mismatch) and focus on specific subgroups, such as different CLAD phenotypes and patients with DSAs.

**Table:** 

*PICO question 7*
In adults with (progressive) CLAD, should antifibrotics be used?

*Recommendation*
• In adults with (progressive) CLAD phenotype BOS, we suggest not using antifibrotic therapy with pirfenidone. (Conditional recommendation against the intervention, low certainty of evidence) • In adults with (progressive) CLAD phenotype RAS, we suggest either using antifibrotic therapy or not using it. (Conditional recommendation for either the intervention or the comparison, low certainty of evidence)

*Remarks*
We do not suggest using antifibrotic therapy with pirfenidone in BOS patients given the lack of efficacy. However, antifibrotic therapy might be considered in RAS patients, as – in theory – it might be more effective in this CLAD phenotype demonstrating more extensive fibrosis, although more data are needed to better understand the effect size in this patient population

#### Summary of evidence

Two RCTs have evaluated pirfenidone use in lung transplant recipients [100, 101]. No published studies of nintedanib were identified at the time of writing, although there is a recently completed RCT in BOS (NCT03283007) [102].

The STOP-CLAD trial did not meet enrollment goals and was prematurely ended; therefore, it was underpowered for the primary outcome, which was change in radiographic assessment of small airways disease. The study included 23 patients (14 BOS and 9 RAS; 12 pirfenidone and 11 placebo), and there was no significant difference in FEV1 change over the 24-week study period between pirfenidone and placebo (−3.1% vs. −3.7%; *p* = 0.90). No life-threatening adverse events were reported. Five serious adverse events requiring medical intervention occurred, all in the placebo group, including four infections requiring antibiotic treatment and one case of hyponatremia [100].

In the EPOS trial (90 BOS patients; 48 pirfenidone and 42 placebo), both the pirfenidone and control groups showed a similar continued FEV1 decline. Secondary endpoints (graft loss, death, re-transplantation) and the incidence of serious adverse events were similar between groups [101].

As pirfenidone did not demonstrate superiority over placebo and standard care, we do not recommend it for treating BOS.

#### Other supportive evidence

In addition to serious adverse events, the RCTs reported a similar overall number of adverse events in the pirfenidone and placebo groups [100, 101]; with more gastrointestinal events [100, 101] and fewer infections [100] in patients receiving pirfenidone. Adverse events judged by blinded adjudication to be attributable to the study drug were more common in the pirfenidone group [100].

Two small additional observational studies (9–11 patients) assessed the role of pirfenidone in lung transplant recipients with CLAD [103, 104]. In Vos et al. median 3-year overall survival following RAS diagnosis was 55% and graft loss occurred in 73% of patients, with a median graft loss–free survival of 1.8 years [103]. In the other study, median overall survival from treatment initiation was 7 months [104].

Across the two studies, a non-significant reduction in the rate of FEV1 decline was observed post- vs. pre-pirfenidone at 3 and 6 months [103, 104], with a significant decrease at 12 months in the study by Vos et al. [103] (−50 to −10 mL/month; *p* < 0.05). However, only 6/11 (55%) patients could still be evaluated at that time point.

Antifibrotics may cause notable gastrointestinal symptoms, requiring dose reduction or treatment discontinuation. Dose adjustment due to anorexia or nausea was reported in 55% of patients [103], while a single case required treatment interruption for gastrointestinal toxicity in the other study [104]. Additional risks include phototoxicity and hepatotoxicity.

The final results of the Pirfenidone for Restrictive Chronic Lung Allograft Dysfunction (PIRCLAD) study, a single-center study on safety and tolerability of pirfenidone in restrictive CLAD, are not available yet (NCT03359863) [105]. A multicenter RCT evaluating nintedanib in lung transplant recipients with BOS Grade 0p-1–2 (INFINITx BOS) was recently completed, and the results are awaited (NCT03283007) [102]. Other novel antifibrotics, such as nerandomilast [106], may warrant investigation in CLAD. Inhaled antifibrotics, currently studied in interstitial lung diseases, could be explored for future research in CLAD-RAS [107].

#### Justification of recommendation

Upon review of the current scientific evidence, we do not suggest using pirfenidone for CLAD-BOS, as there is no substantial indication of efficacy. Conversely, data are insufficient to draw conclusions on antifibrotic therapy for CLAD-RAS and further research is warranted.

#### Implementation considerations

Limited availability and reimbursement may restrict implementation. In some countries, nintedanib may be prescribed for CLAD-RAS when classified as progressive pulmonary fibrosis and/or as an off-label treatment. Patients should be monitored for adverse effects, including gastrointestinal symptoms, hepatotoxicity, and skin toxicity. Additionally, trough levels of CNI should be assessed due to the potential for drug interactions [103].

#### Future research

Based on the anticipated outcomes from the PIRCLAD study involving patients with restrictive CLAD, further investigation in this patient population might be recommended. Studies with other antifibrotics, such as nerandomilast, could be considered.

**Table:** 

*PICO question 8*
In adults with (progressive) CLAD, should montelukast be used?

*Recommendation*
• In adults with (progressive) CLAD, we suggest either using montelukast or not using it. (Conditional recommendation for either the intervention or the comparison, low certainty of evidence)

*Remarks*
Montelukast may be effective in cases of early BOS. In patients with new-onset BOS stage 1, a trial of montelukast can be considered

#### Summary of evidence

One RCT (30 patients; 15 montelukast, 15 placebo) assessed montelukast in lung transplant recipients with BOS and slowly progressive FEV1 decline despite prior azithromycin [108].

Graft loss at 1 and 2 years was similar in montelukast and control groups. Overall, montelukast had no significant effect on FEV1 decline, but a *post-hoc* subgroup analysis demonstrated a decrease in FEV1 decline in BOS stage 1 patients, which was not seen in BOS stage 2–3 patients.

Serious adverse events were similar between groups, and treatment discontinuation rates did not differ significantly. Rates of respiratory infections were also comparable.

#### Other supportive evidence

Two additional observational studies assessed montelukast in CLAD patients [109, 110]; all studies were from the same center.

In the largest study (153 patients; 115 BOS, 38 RAS), montelukast stabilized or improved FEV1 in 81% of cases at 3 months, with benefits persisting up to 12 months, and responders showing superior progression-free and overall survival compared with non-responders [110]. A smaller study (n = 22) reported slower FEV1 decline with montelukast compared to standard therapy (13 vs. 114 mL/month) [109].

Overall, these data suggest montelukast may stabilize lung function and delay disease progression in selected CLAD patients. Rapid decliners were less likely to respond [110] and were excluded from the randomized trial [108].

Based on available data on pulmonary chronic graft-versus-host disease (BOS-phenotype) following allogeneic hematopoietic stem cell transplantation, montelukast is recommended as part of the standard first-line “FAM” therapy, which includes inhaled corticosteroids, azithromycin, and montelukast [111]. Nevertheless, the magnitude of montelukast’s therapeutic effect in this context remains uncertain.

No severe adverse events were attributed to montelukast; some patients reported vivid dreams [110]; such sleep disturbances, including nightmares, are known from clinical experience. Rare neuropsychiatric adverse events are recognized from broader use in asthma and allergic disease, leading the U.S. Food and Drug Administration to issue a Boxed Warning for montelukast regarding serious mental health–related adverse effects [112]. Although data largely derive from non-transplant populations, they are relevant to transplant recipients, who experience neuropsychiatric vulnerability. No severe neuropsychiatric events were reported in the study by Vos et al. [110], and such effects appear uncommon from clinical experience.

Overall, data suggest an acceptable safety profile with a low likelihood of serious harm in the transplant population.

#### Justification of recommendation

Overall, the RCT did not demonstrate an effect of montelukast on FEV1; however, some benefit was observed in patients with early-stage BOS (BOS stage 1). Therefore, montelukast may be considered in this group, with discontinuation advised in the event of disease progression.

#### Implementation considerations

The advantage of montelukast compared with other treatments (*e.g*., ECP or ATG) is its oral administration and low costs. Treatment response should be monitored and discontinuation should be considered in the absence of response. Patients should also be monitored for neurological and psychiatric adverse effects, and changes in sleep patterns.

#### Future research

The Task Force did not formulate specific future research recommendations for montelukast, as they perceive priorities rather lie elsewhere.

### Treatment dosages and schedules

The most common dosages and schedules of CLAD therapeutics are summarized in Table 2. Since many studies in this field are single center, the dosages used also vary. Regarding tacrolimus, different target levels have been used even in RCTs. The target levels for tacrolimus from the ScanCLAD trial were 10–14 ng/mL at 0–3 months, 8–12 ng/mL at 3–6 months, 8–10 ng/mL at 6–12 months, and 6–8 ng/mL beyond 12 months [4]. A recently published guideline for German-speaking countries for follow-up care after lung transplantation summarizes the target levels for tacrolimus used in other studies [12].

## Conclusion

The ESOT Task Force recommendations on prevention and treatment of CLAD are summarized in Table 3 and displayed in Figures 1–3. Future research recommendations for prevention and treatment are provided in Tables 4–5.

**TABLE 3:** Overview of recommendations.

PICO number	PICO question	Recommendation
PICO 1	In adult lung transplant recipients, should azithromycin be used to prevent the onset of CLAD?	In adult lung transplant recipients, we suggest using azithromycin to prevent the onset of CLAD *Conditional recommendation in favor of the intervention, very low certainty of evidence*
PICO 2	In adult lung transplant recipients, should tacrolimus be used over cyclosporine to prevent the onset of CLAD?	In adult lung transplant recipients, we recommend using tacrolimus over cyclosporine in the prevention of CLAD *Strong recommendation in favor of the intervention, low certainty of evidence*
PICO 3	In adult lung transplant recipients, should mycophenolate mofetil be used over azathioprine to prevent the onset of CLAD?	In adult lung transplant recipients, we suggest using either mycophenolate mofetil or azathioprine to prevent the onset of CLAD *Conditional recommendation for either the intervention or the comparison, low certainty of evidence*
PICO 4	In adults with (progressive) CLAD, should extracorporeal photopheresis be used?	In adults with (progressive) CLAD, we suggest either using extracorporeal photopheresis or not using it *Conditional recommendation for either the intervention or the comparison, very low certainty of evidence*
PICO 5	In adults with (progressive) CLAD, should anti-thymocyte globulin be used?	In adults with (progressive) CLAD, we suggest either using anti-thymocyte globulin or not using it *Conditional recommendation for either the intervention or the comparison, very low certainty of evidence*
PICO 6	In adults with (progressive) CLAD, should alemtuzumab be used?	In adults with (progressive) CLAD, we suggest either using alemtuzumab or not using it *Conditional recommendation for either the intervention or the comparison, very low certainty of evidence*
PICO 7	In adults with (progressive) CLAD, should antifibrotics be used?	In adults with (progressive) CLAD phenotype BOS, we suggest not using antifibrotic therapy with pirfenidone *Conditional recommendation against the intervention, low certainty of evidence* In adults with (progressive) CLAD phenotype RAS, we suggest either using antifibrotic therapy with pirfenidone or not using it *Conditional recommendation for either the intervention or the comparison, low certainty of evidence*
PICO 8	In adults with (progressive) CLAD, should montelukast be used?	In adults with (progressive) CLAD, we suggest either using montelukast or not using it *Conditional recommendation for either the intervention or the comparison, low certainty of evidence*

BOS, bronchiolitis obliterans syndrome; CLAD, chronic lung allograft dysfunction; RAS, restrictive allograft syndrome.

**FIGURE 1:**
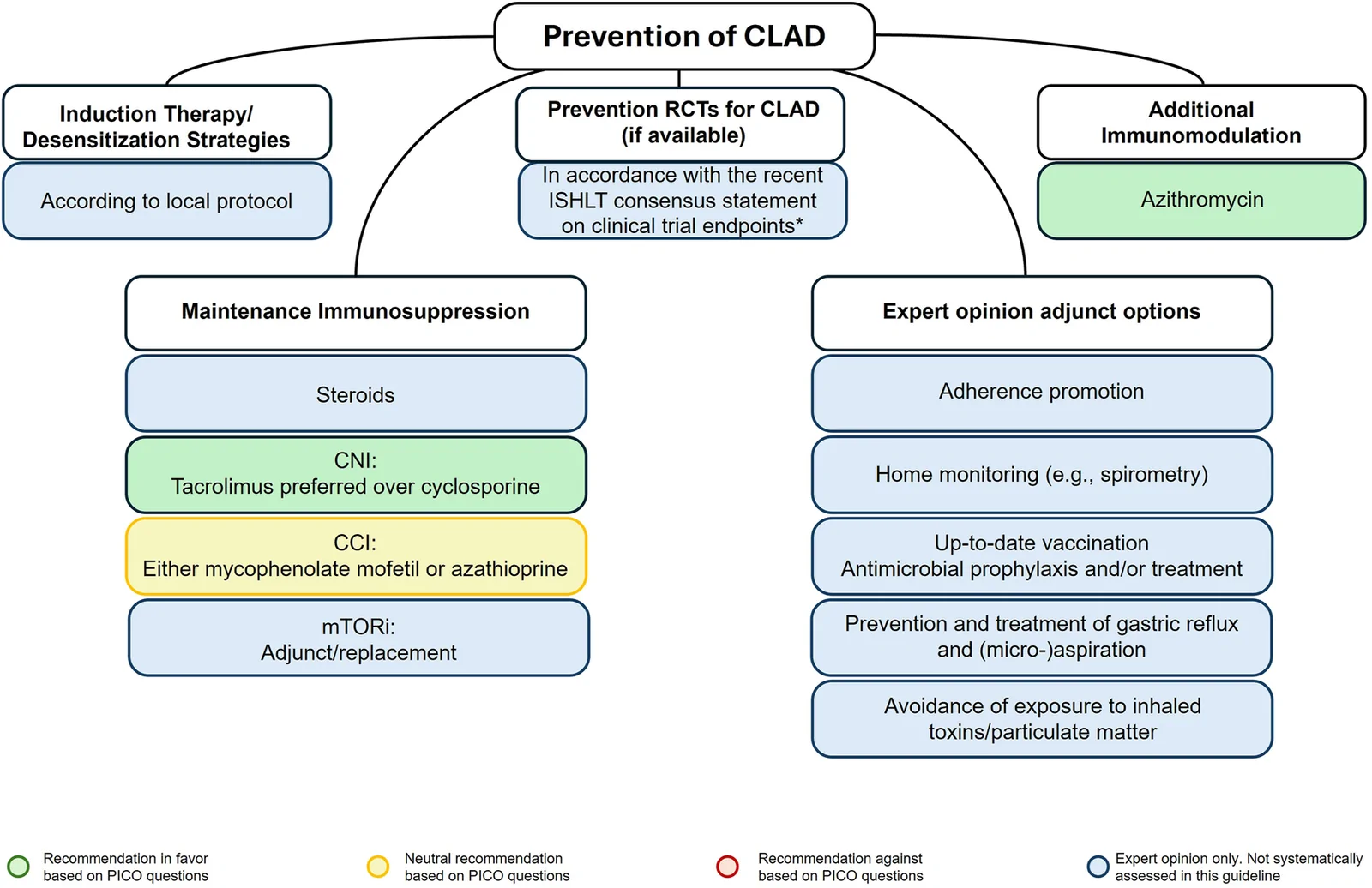
Recommendations for prevention of chronic lung allograft dysfunction. Overview of the different preventive strategies for chronic lung allograft dysfunction (CLAD) discussed in this Clinical Practice Guideline.

**FIGURE 2:**
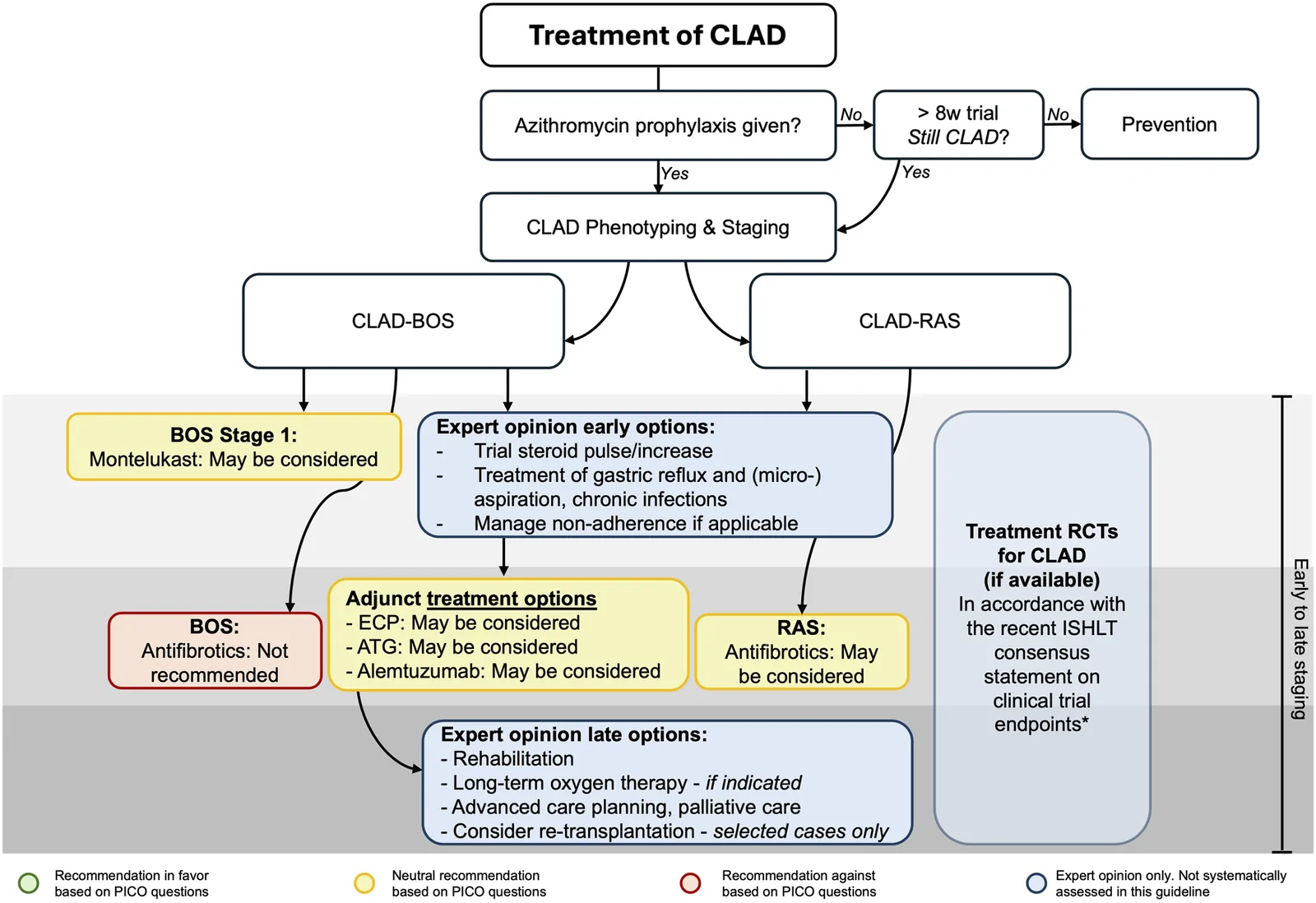
Recommendations for treatment of chronic lung allograft dysfunction. Overview of the different treatment strategies for chronic lung allograft dysfunction (CLAD) discussed in this Clinical Practice Guideline.

**FIGURE 3:**
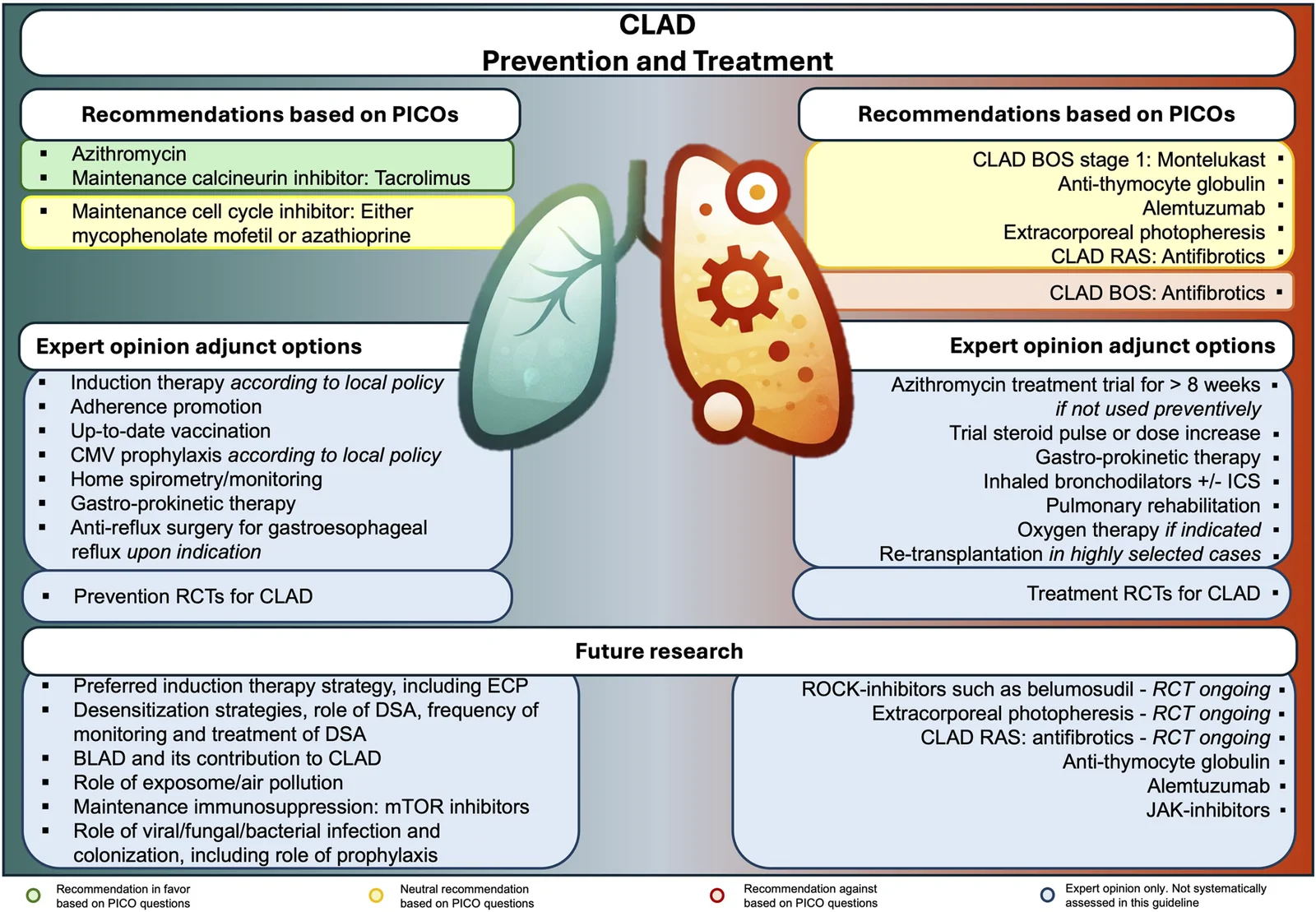
Summary of prevention and treatment strategies for chronic lung allograft dysfunction. Overview of the different preventive and treatment strategies for chronic lung allograft dysfunction (CLAD) discussed in this Clinical Practice Guideline.

**TABLE 4:** Research recommendations regarding prevention.

Future research recommendations: prevention	
Azithromcyin	⁃ Optimal timing of initiation after lung transplantation ⁃ Whether treatment should be applied universally or targeted to specific risk groups ⁃ Patient-reported outcome measures ⁃ Evaluation of long-term effects, including impact on graft and overall survival
Tacrolimus vs. cyclosporine	⁃ Long-term data on overall survival and graft survival; updated results from the ScanCLAD study are anticipated ⁃ Comparative effects of once-daily, extended release vs. twice-daily, immediate-release dosing and of different tacrolimus formulations on renal outcomes and other side effects ⁃ Management of unacceptable side effects, including thrombotic microangiopathy, posterior reversible encephalopathy syndrome ⁃ Comparative quality of life outcomes with tacrolimus vs. cyclosporine in lung transplantation; follow-up reports from the ScanCLAD study are expected ⁃ Optimal trough levels for CNI at different time points after transplantation, including feasibility and safety of dose reduction ⁃ Adjustment of CNI target levels based on kidney function, immunological risk, risk factors, and drug–drug interactions ⁃ CNI-reduction or CNI-free immunosuppressive strategies, such as mTOR inhibitor-based quadruple low CNI maintenance immunosuppressive regimen vs. standard triple maintenance immunosuppressive regimen ⁃ Best practices for therapeutic drug monitoring, including frequency, methodology, and dose adjustment ⁃ Cost-effectiveness of tacrolimus compared with cyclosporine in lung transplantation
MMF vs. AZA	⁃ Optimal dosing, considering pharmacogenetic factors beyond lymphopenia ⁃ Definition of patient subgroups (*e.g*., those with telomeropathies) that may benefit preferentially from one agent ⁃ Development of DSAs in patients receiving MMF vs. AZA ⁃ Long-term effects of each cell cycle inhibitor ⁃ Alternatives of cell cycle inhibitors, such as the use of mTOR inhibitors instead of MMF or AZA ⁃ Timing and management of MMF discontinuation during infections and its impact on CLAD incidence ⁃ Guidance on pausing cell cycle inhibitors around vaccinations
Other	⁃ Clinical trials with novel drugs for preventing CLAD are an unmet need, and the Task Force recommends conducting well-designed RCTs (ideally multicenter RCTs and the use of CLAD adjudication) for CLAD prevention, in accordance with the recent ISHLT consensus statement on Lung Transplant Clinical Trials [105] ⁃ Further studies on long-term outcomes of organ preservation strategies are needed to inform about the effects on CLAD incidence ⁃ The use of specific HLA antibody/DSA-directed treatments and whether they reduce the risk of later CLAD development ⁃ In patients with AMR, more data are needed on the efficacy of AMR treatments as a prevention of CLAD ⁃ The role of inhaled immunosuppressive drugs, such as inhaled cyclosporine and mTOR inhibitors, in the prevention of CLAD ⁃ Whether the addition of other immunomodulating or immunosuppressive drugs, such as belumosudil, ECP or tocilizumab, to standard triple immunosuppression has an impact on CLAD incidence

AMR, antibody-mediated rejection; AZA, azathioprine; CLAD, chronic lung allograft dysfunction; CNI, calcineurin inhibitor; DSA, donor-specific antibody; ECP, extracorporeal photopheresis; HLA, human leukocyte antibody; MMF, mycophenolate mofetil; mTOR, mammalian target of rapamycin.

**TABLE 5:** Research recommendations regarding treatment.

Future research recommendations: treatment	
ECP	⁃ Definition and prediction of treatment response ⁃ Consolidation of subgroup analyses to better define responder profiles ⁃ Harmonization of practices concerning the frequency and duration of ECP, including RCTs on continuation vs. discontinuation in stable patients under treatment ⁃ Evaluation of novel treatment targets, including the use of biomarkers, patient-reported outcome measures (e.g., quality of life and patient burden), acceptable and unacceptable side effects/adverse events, and central line requirement ⁃ Development of comparative cost-effectiveness studies ⁃ Beyond its role in CLAD management, more data is needed on the role of ECP in CLAD prevention
ATG	⁃ RCTs are warranted to better define efficacy and safety ⁃ Subgroup analyses to better define responder profiles, including different CLAD phenotypes ⁃ Determination of optimal dosing, treatment duration, and appropriate monitoring strategies ⁃ Data on high-risk groups, such as EBV-mismatched recipients at increased PTLD risk
Alemtuzumab	⁃ RCTs are necessary to better define efficacy and safety ⁃ Subgroup analyses to better define responder profiles, including different CLAD phenotypes and patients with DSAs ⁃ Determination of optimal dosing and scheduling of alemtuzumab ⁃ Better risk assessment for adverse events (*e.g*., PTLD risk in patients with EBV mismatch)
Antifibrotics	⁃ For the RAS phenotype, further investigation should be decided based on the results from the PIRCLAD study ⁃ Studies with other antifibrotics, such as nerandomilast
Other	⁃ Clinical trials with novel drugs in CLAD are an unmet need, and the Task Force recommends conducting well-designed RCTs (ideally multicenter RCTs) for CLAD treatment, in accordance with the recent ISHLT consensus statement on Lung Transplant Clinical Trials [105] ⁃ More data is needed on the efficacy and safety of JAK inhibitors in CLAD ⁃ More data is needed on the efficacy and safety of ROCK inhibitors in CLAD ⁃ More data is needed on the efficacy and safety of mesenchymal stem cells in CLAD ⁃ Data are required on other novel potential disease-modifying drugs

ATG, anti-thymocyte globulin; DSAs, donor-specific antibodies, ECP, extracorporeal photopheresis; JAK, Janus kinase; PTLD, post-transplant lymphoproliferative disease; RCT, randomized controlled trials; ROCK, Rho kinase.

CLAD remains the primary obstacle to long-term success after lung transplantation. It imposes a heavy disease burden, leading to substantial morbidity, diminished quality of life, and premature graft loss in affected recipients. CLAD prevention and treatment are challenging, and guidelines on the management of CLAD are lacking. This ESOT clinical practice guideline on the prevention and treatment of CLAD—developed by a multidisciplinary Task Force including patient and allied healthcare representation, using systematic literature reviews and GRADE methodology—offers evidence-based recommendations to guide contemporary clinical practice.

For prevention, we recommend tacrolimus over cyclosporine as the preferred CNI and conditionally suggest prophylactic azithromycin to delay or reduce the onset of CLAD. MMF and AZA are regarded as comparable cell cycle inhibitors; the choice between them and, potentially, other agents (*e.g*., mTOR inhibitors) should be individualized according to gastrointestinal tolerance, risk of myelotoxicity, fertility considerations, and other patient-specific factors.

For established (progressive) CLAD, no standardized or highly effective treatment currently exists. Clinical studies are largely limited to surrogate endpoints, such as preservation of graft function measured by FEV1, while data on hard clinical outcomes, including patient survival and patient-relevant outcomes such as quality of life, remain limited. Most interventions, including ECP, ATG, alemtuzumab, and montelukast, received only conditional recommendations owing to very low to low certainty of evidence. Antifibrotic treatment with pirfenidone is not suggested in patients with CLAD-BOS phenotype, but antifibrotics could be considered in patients with the RAS phenotype, pending more robust data. No therapy has been conclusively shown to halt or reverse disease progression. Retransplantation, while potentially curative, is unfortunately available to only a small minority of patients due to organ scarcity, procedural complexity and patient-specific comorbidities limiting candidacy.

These largely conditional recommendations highlight major evidence gaps. High-quality RCTs, conducted in accordance with the recent International Society for Heart and Lung Transplantation clinical trials consensus statement [113], standardized management pathways, responder identification, and inclusion of patient-reported outcomes are urgently required to better inform future recommendations on the management of CLAD. In the interim, individualized risk–benefit assessment, shared decision-making, prevention and treatment of modifiable risk factors (*e.g*., non-adherence, infections, gastroesophageal reflux), and close monitoring remain essential to preserve the best possible graft function after lung transplantation.

## Data Availability

The original contributions presented in the study are included in the article/Supplementary Material, further inquiries can be directed to the corresponding author.
