# Supplementary material for: European society for organ transplantation clinical practice guideline on prevention and treatment of chronic lung allograft dysfunction
Source: Transpl Int. 2026 Sep 18;39:16959. doi: 10.3389/ti.2026.16959 (PMC13630708; doi:10.3389/ti.2026.16959)
Supplement: Supplementary file 1 [file DataSheet1.pdf]

European Society for Organ Transplantation Clinical Practice Guideline on Prevention and Treatment of Chronic Lung Allograft Dysfunction: Online Supplement

Supplement 1: Methods, search strategies and results

Table of contents

Composition of the Task Force 2
Disclosure of potential conflicts of interest 3
Detailed methodology 4
Decision-making on PICO questions 4
Literature search and screening 4
Inclusion and exclusion criteria 4
Selection of outcomes 5
Data extraction 5
Assessment of the level of evidence and degree of recommendations 5
PRISMA flow diagrams 6
References 14

Composition of the Task Force

Members primarily from the European Society for Organ Transplantation and European Reference Networks - LUNG Core Network on Chronic Lung Allograft Dysfunction were approached to participate in this Clinical Practice Guideline to form a geographically and gender balanced, collaborative, international working group, including highly experienced physicians, an allied-healthcare professional and patient representation.

Name	Specialty
Robin Vos	Consultant in respiratory medicine and lung transplantation<br>Chair
Jens Gottlieb	Consultant in respiratory medicine and lung transplantation<br>Chair
Saskia Bos	Consultant in respiratory medicine and lung transplantation<br>Junior Chair<br>Methodology
Merel Hellemons	Consultant in respiratory medicine and lung transplantation<br>Junior Chair<br>Methodology
Kavita Dave	Consultant in respiratory medicine and lung transplantation
Maarten Engel	Information specialist
Are Holm	Consultant in respiratory medicine and lung transplantation
Letizia Morlacchi	Consultant in respiratory medicine and lung transplantation
Veronika Müller	Consultant in respiratory medicine and lung transplantation
Stefano Pavanello	Patient representative<br>Director of the European Pulmonary Fibrosis Federation
Clément Picard	Consultant in respiratory medicine and lung transplantation
Veronica Rossi	Respiratory physiotherapist
Berta Saez-Gimenez	Consultant in respiratory medicine and lung transplantation

Disclosure of potential conflicts of interest

All potential conflicts of interest related to this guideline were declared at commencement of this Task Force; compliance was monitored by the Chairs. Members with potential conflicts of interest refrained from screening related PICO questions and voting on these specific recommendations. Furthermore, the Task Force members involved in the methodological steps (SB, MH) were not responsible for PICO questions with potential conflicts of interest. The information specialist and medical writer were non-voting members of the panel.

PICO question	Members with possible conflicts of interest
PICO 1	Robin Vos
PICO 2	/
PICO 3	/
PICO 4	Saskia Bos, Kavita Dave
PICO 5	/
PICO 6	/
PICO 7	Clément Picard
PICO 8	Robin Vos

Detailed methodology

Decision-making on PICO questions

Due to the limited number of PICO questions that could be included, it was not possible to address all therapeutic options considered clinically relevant to the prevention and treatment of CLAD. Following initial discussions, the Task Force members voted on the following potential PICO questions to reach a consensus on the eight final PICO questions to be included. Members could vote from -1 if they were against, to +2 if they were really in favour of including the PICO question. The total voting score is provided after each PICO question.

- In adult lung transplant recipients, should azithromycin be used to prevent the onset of CLAD? 22
- In adults with (progressive) CLAD, should extracorporeal photopheresis be used? 22
- In adult lung transplant recipients, should tacrolimus be used over cyclosporine to prevent the onset of CLAD? 21
- In adult lung transplant recipients, should mycophenolate mofetil be used over azathioprine to prevent the onset of CLAD? 18
- In adults with (progressive) CLAD, should antifibrotics be used? 15
- In adults with (progressive) CLAD, should anti-thymocyte globulin be used? 14
- In adults with (progressive) CLAD, should montelukast be used? 13
- In adults with (progressive) CLAD, should alemtuzumab be used? 12
- In adults with CLAD-RAS, should antifibrotics be used? 9
- In adults with (progressive) CLAD, should JAK-inhibitors be used? 7
- In adults with (progressive) CLAD, should total lymphoid irradiation be used? 6
- In adults with (progressive) CLAD, should fundoplication be considered in patients with gastro-oesophageal reflux disease? 6
- In adults with (progressive) CLAD, should mTOR inhibitors be used? 5
- In adults with (progressive) CLAD, should steroids dosage be increased? 5
- In adults with end-stage CLAD, should re-transplantation be performed? 3
- In adult lung transplant recipients, should PPI be used to prevent the onset of CLAD? -2

Literature search and screening

The literature search for all PICO questions was based on a priori defined inclusion and exclusion criteria outlined below and was performed by an experienced information specialist (ME). The PubMed platform was used to search MEDLINE, and the databases of EMBASE and Cochrane Library were also searched. Initial searches were performed in October 2024 and updated in December 2025; three additional relevant references were included.

Covidence systematic review software (Veritas Health Innovation, Melbourne, Australia) was used for screening the studies identified by the literature searches. Literature was screened in two phases: title/abstract and full text screening. All studies were independently screened by Task Force members (PICO 1: KD, AH, VR; PICO 2: JG, AH; PICO 3: LM, RV; PICO 4: CP, RV; PICO 5: CP, BSG; PICO 6: VM, VR, BSG; PICO 7: KD, LM; PICO 8: JG, VM), with resolution of conflicts by a third independent reviewer (SB/MH). For each question, a Preferred Reporting Items for Systematic Reviews and Meta-Analyses (PRISMA) diagram documented the study selection process, see below.[1]

Inclusion and exclusion criteria

Our generic inclusion criterion was adults aged ≥ 18 years (or if mixed pediatric/adult population: if median age or majority of patients were 18 or older) with CLAD (all phenotypes). Our hierarchy of evidence was systematic reviews and randomized controlled trials, followed by prospective and retrospective observational studies and case series. Case reports were excluded. For the main body of evidence, we prioritized randomized controlled trials. When these were unavailable, we included comparative observational studies, and in their absence, non-comparative observational studies,

based on availability, addressing each of the PICO questions. If available and relevant, other studies were tabulated as additional evidence.
The search was restricted to studies published in English. No restriction regarding time of publication was used.

Selection of outcomes
Following GRADE guidance, the panel selected outcomes of interest for each PICO question a priori, based on their relative importance to adult patients with CLAD and to clinical decision-making.[2] Following the GRADE approach, outcomes were rated on a 1-9 scale as "critical" (7-9), "important" (4-6) or "of limited importance" (1-3) for clinical decision-making through an online vote using Google Docs.[3] All votes were pooled together and the mean value for each outcome was calculated. Based on further discussions, each reported outcome was then designated to one of the three levels of importance. Only outcomes that were considered critical or important were included in data extraction and further analysis and used to formulate the recommendations. An overview of included outcomes per PICO question is provided in Table 1.

Table 1. Critical and important outcomes per PICO question

**PICO 1-3: prevention**		**PICO 4-8: Treatment**	
Critical	Graft loss<br>Overall survival<br>CLAD onset<br>Serious adverse events	Critical	Graft loss<br>Overall survival<br>Serious adverse events
Important	Quality of life<br>Infections<br>Kidney function<br>Malignancies	Important	Quality of life<br>FEV1<br>Malignancies<br>Kidney function

FEV1: forced expiratory volume in one second.

Data extraction
Data extraction was performed by all subgroups and checked by the Junior Chairs (SB, MH). Risk of bias of the main body of evidence for each PICO question was performed by one of the Junior Chairs and checked by the other Junior Chair. Randomized controlled trials were assessed using the revised Cochrane risk-of-bias tool.[4] The Newcastle Ottawa Scale was used to assess the risk of bias of non-randomised studies.[5]

Assessment of the level of evidence and degree of recommendations
We followed the GRADE approach to assess the certainty of evidence and degree of recommendations.[3] This approach specifies four categories of certainty (high, moderate, low and very low) that are applied to a body of evidence and not to individual studies. The body of evidence was evaluated primarily on the basis of risk of bias, consistency, precision, directness of evidence, and risk of publication bias.
Recommendations were graded as strong or conditional after considering the certainty of evidence, the balance of desirable and undesirable outcomes, the assumptions about the relative importance of outcomes, the implications for resource use, and the acceptability and feasibility of implementation, as well as patient values and preferences.
Evidence profiles and Evidence to Decision (EtD) frameworks were generated for each clinical question by the methodology team and each subgroup, respectively. Based on these formats, the panel formulated the clinical recommendations and decided on their strength through consensus and, if required, voting. Following the GRADE approach, strong recommendations were phrased as "we recommend", while conditional recommendations were phrased as "we suggest".[3,6,7]

PRISMA flow diagrams

PICO question 1

In adult lung transplant recipients, should azithromycin be used to prevent the onset of CLAD?

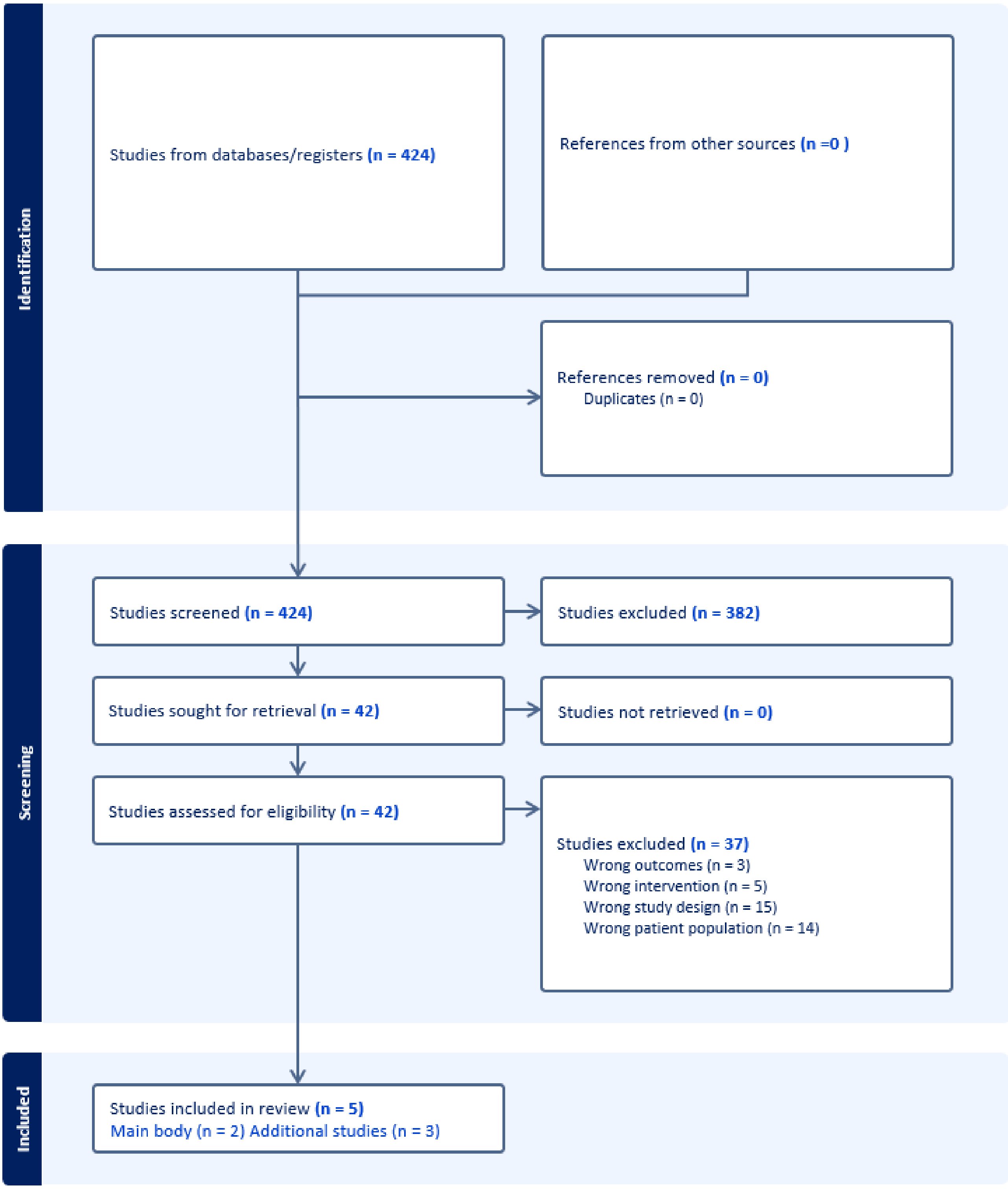

PICO question 2

In adult lung transplant recipients, should tacrolimus be used over cyclosporine to prevent the onset of CLAD?

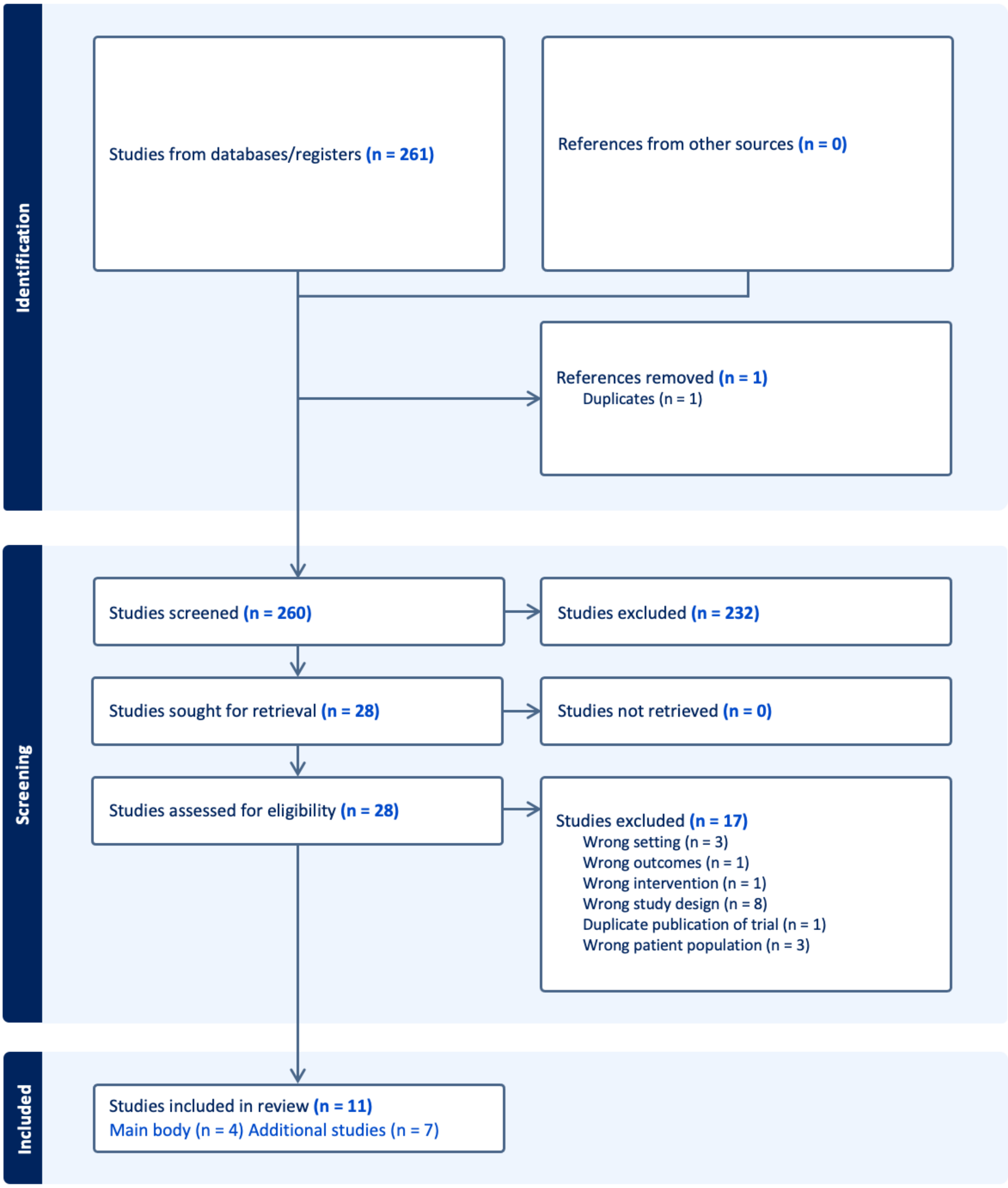

PICO question 3
In adult lung transplant recipients, should mycophenolate mofetil be used over azathioprine to prevent the onset of CLAD?

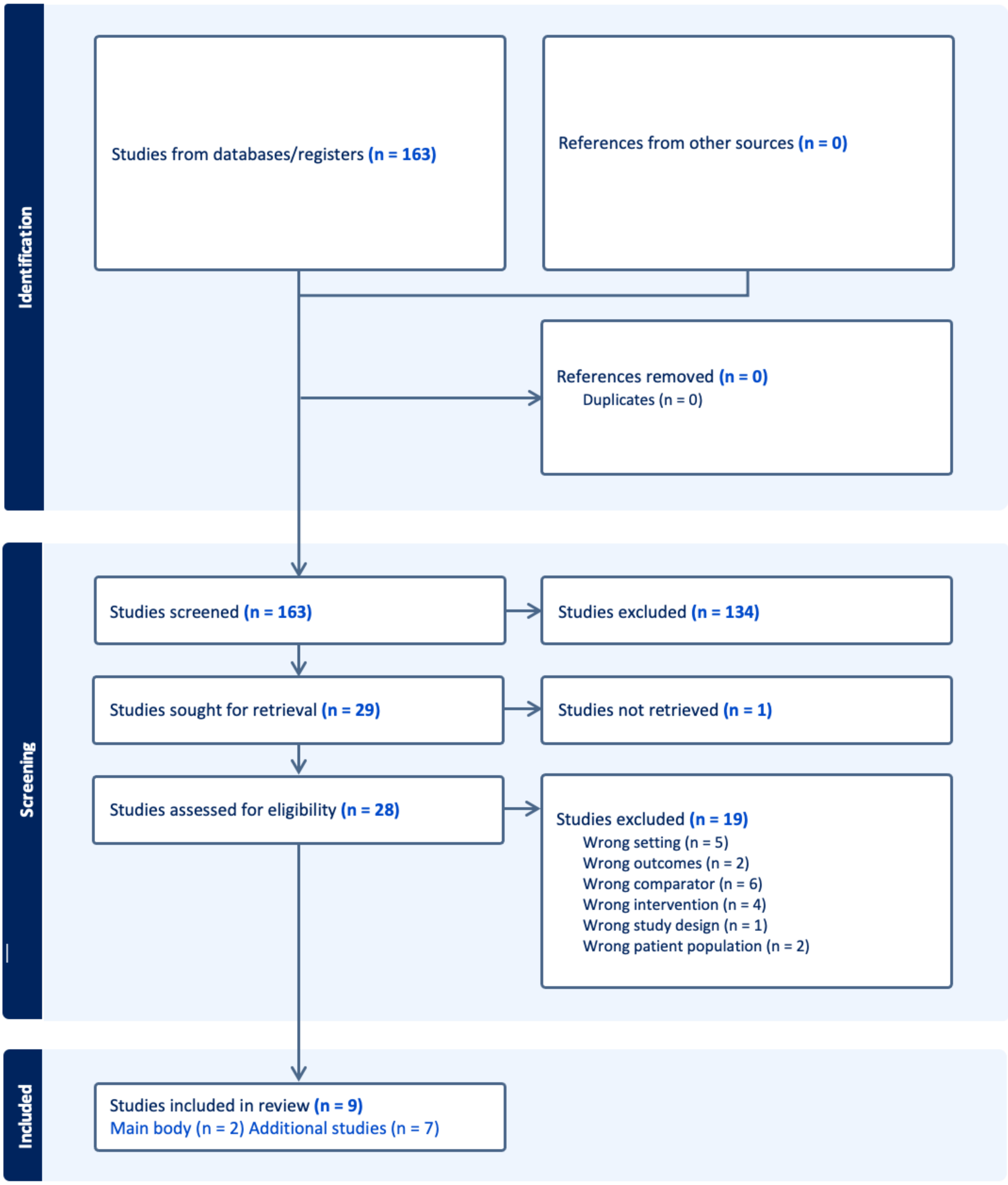

PICO question 4
In adults with (progressive) CLAD, should extracorporeal photopheresis be used?

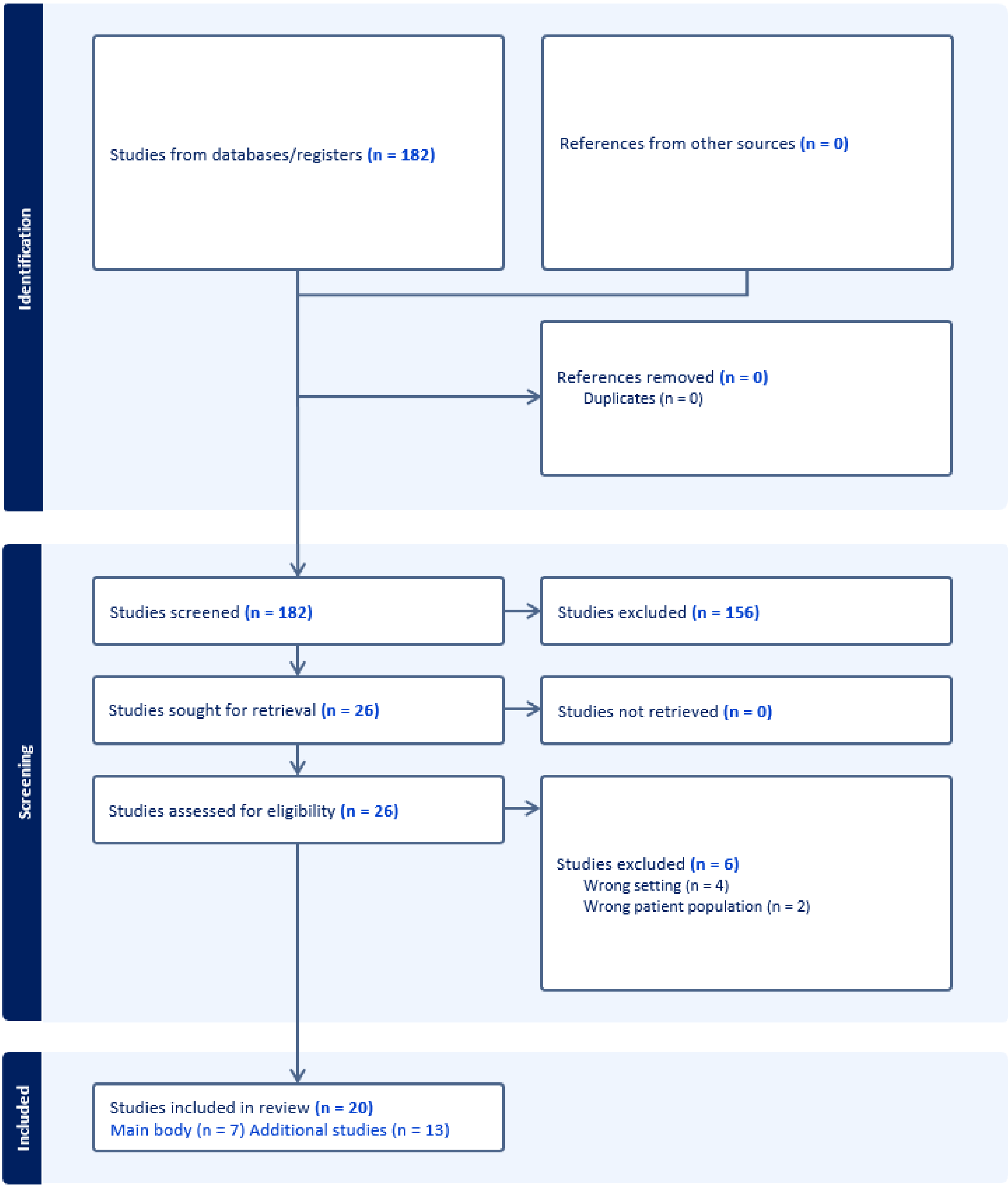

PICO question 5
In adults with (progressive) CLAD, should anti-thymocyte globulin be used?

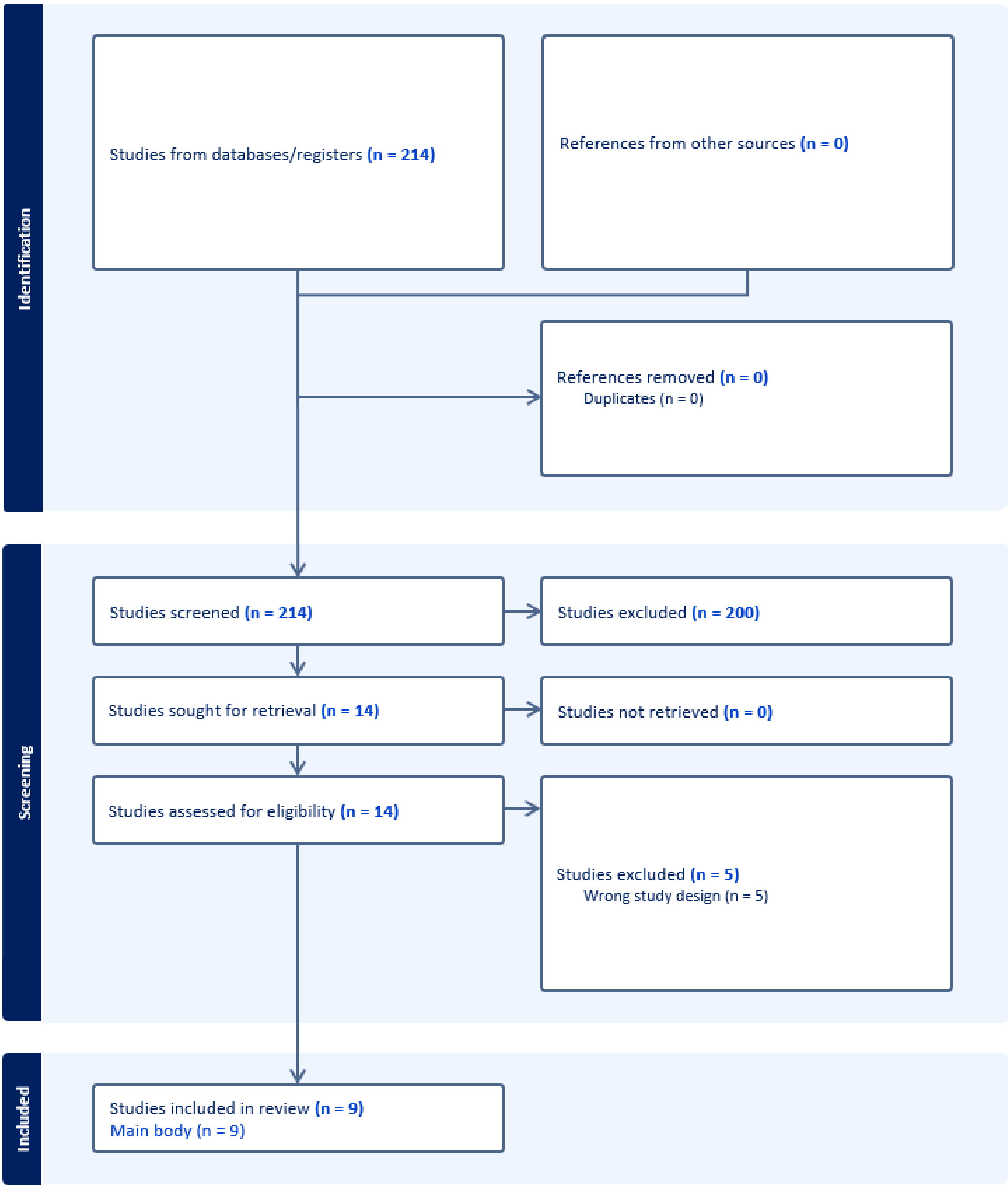

PICO question 6
In adults with (progressive) CLAD, should alemtuzumab be used?

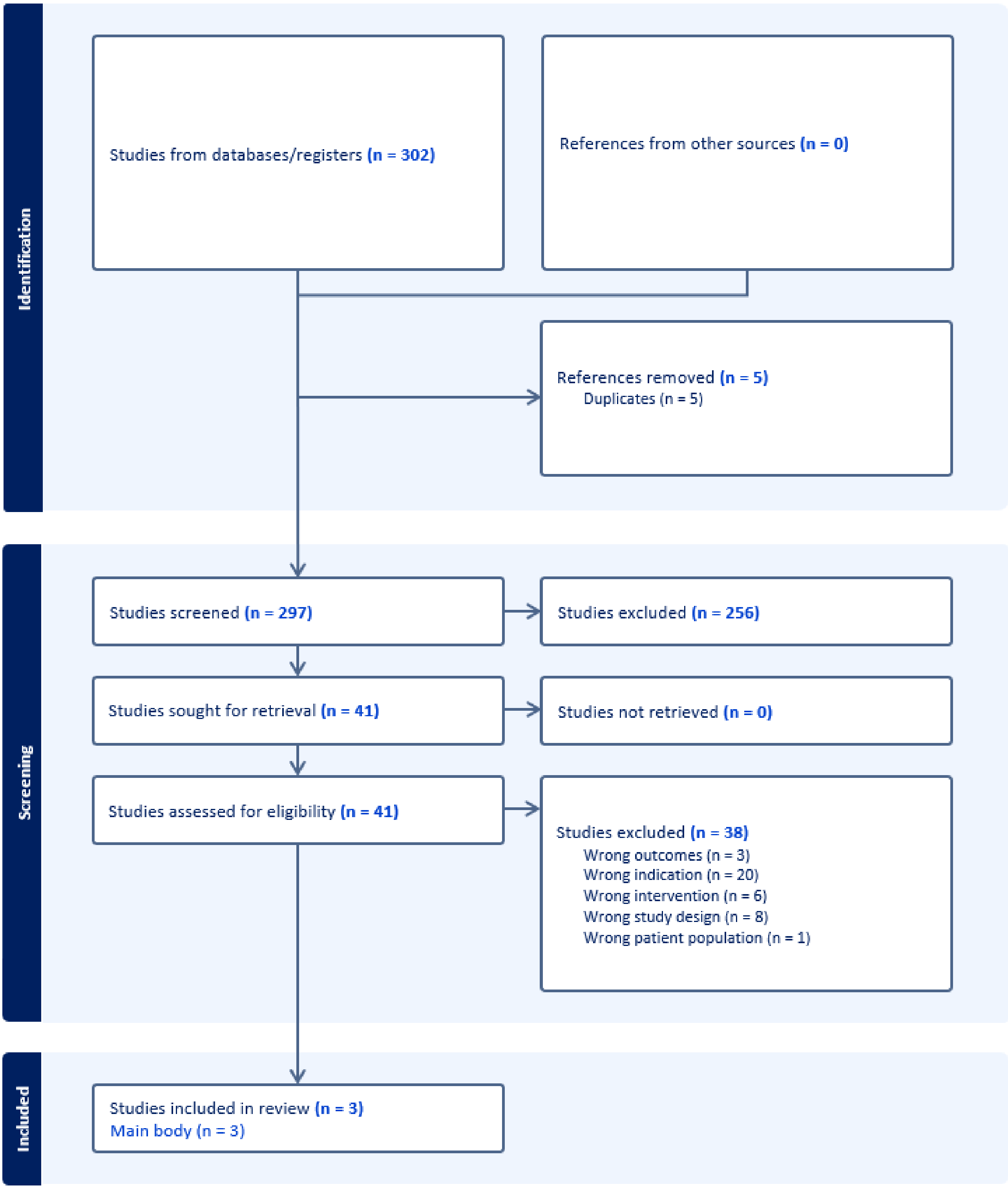

PICO question 7
In adults with CLAD, should antifibrotics be used?

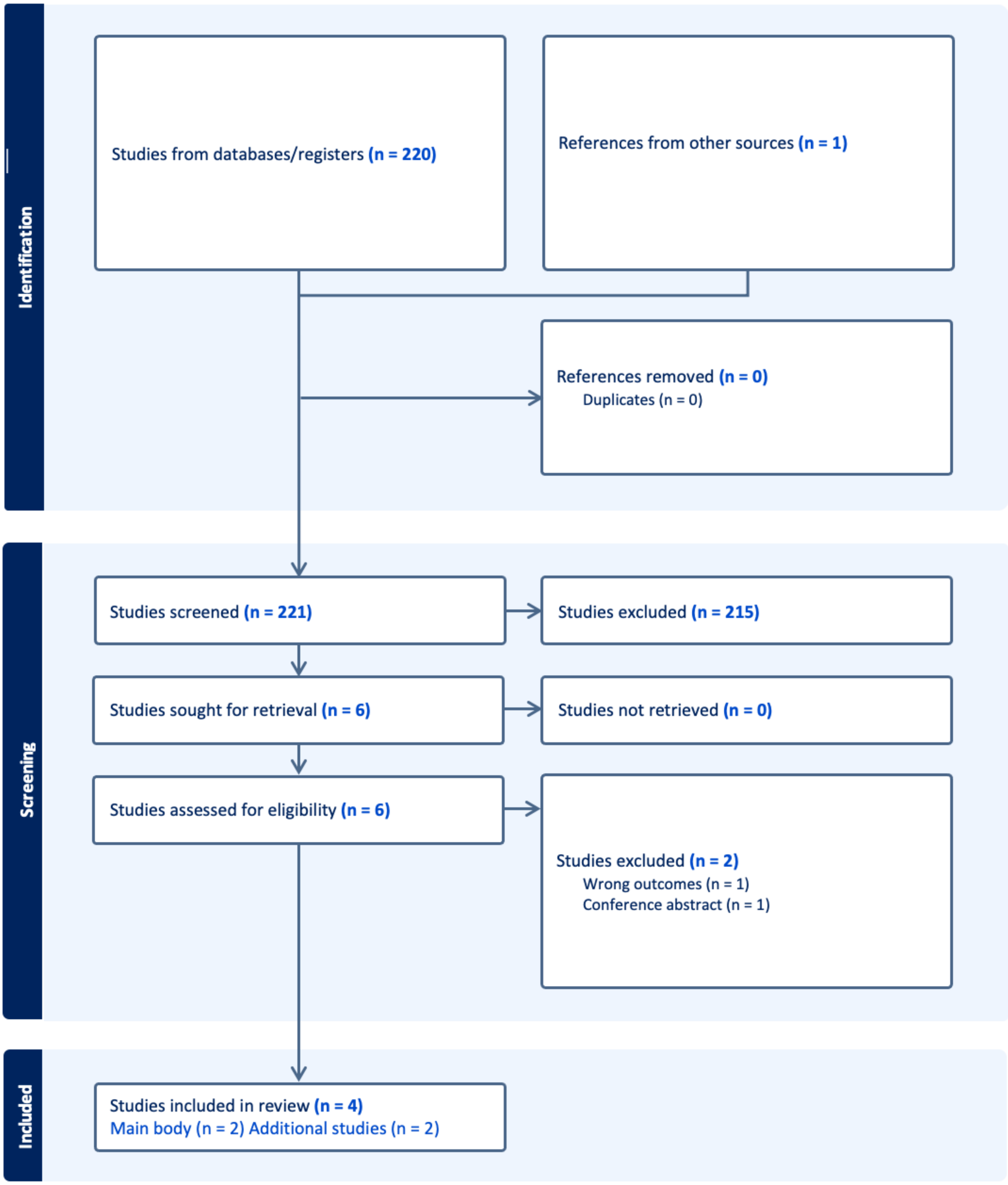

PICO question 8
In adults with (progressive) CLAD, should montelukast be used?

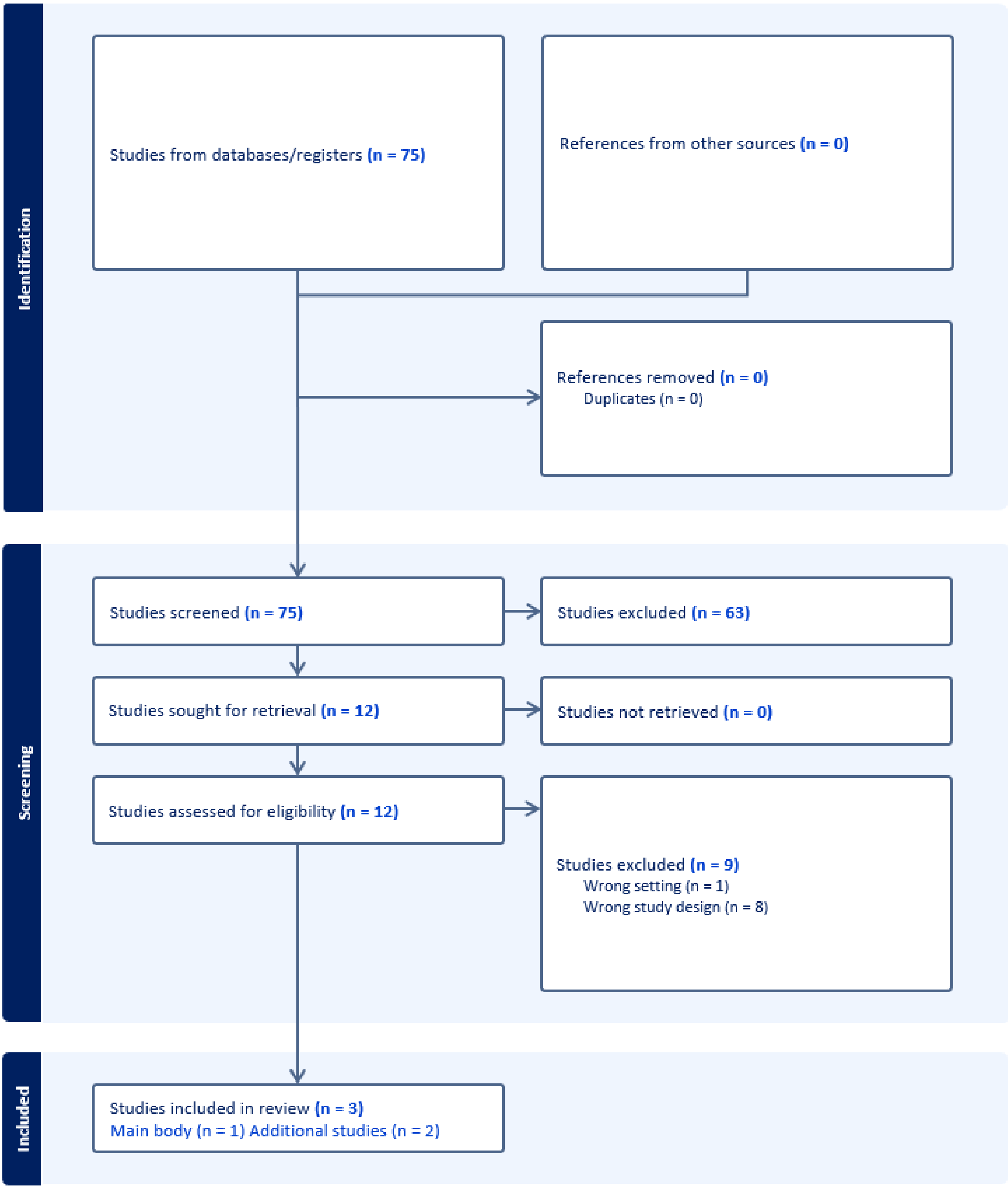

References

1. Page MJ, McKenzie JE, Bossuyt PM, et al. The PRISMA 2020 statement: an updated guideline for reporting systematic reviews. Syst Rev. 2021;10(1):89. https://doi.org/10.1186/s13643-021-01626-4.
2. Guyatt GH, Oxman AD, Kunz R, et al. GRADE guidelines: 2. Framing the question and deciding on important outcomes. J Clin Epidemiol. 2011;64(4):395-400. https://doi.org/10.1016/j.jclinepi.2010.09.012.
3. Brożek JL, Akl EA, Compalati E, et al. Grading quality of evidence and strength of recommendations in clinical practice guidelines part 3 of 3. The GRADE approach to developing recommendations. Allergy. 2011;66(5):588-95. https://doi.org/10.1111/j.1398-9995.2010.02530.x.
4. Sterne JAC, Savović J, Page MJ, et al. RoB 2: a revised tool for assessing risk of bias in randomised trials. BMJ. 2019;366:l4898. https://doi.org/10.1136/bmj.l4898.
5. Wells G, Shea B, O'connell D, et al. The Newcastle-Ottawa Scale (NOS) for assessing the quality of nonrandomised studies in meta-analyses. https://www.ohri.ca/programs/clinical_epidemiology/oxford.asp.
6. Andrews J, Guyatt G, Oxman AD, Alderson P, Dahm P, Falck-Ytter Y, et al. GRADE guidelines: 14. Going from evidence to recommendations: the significance and presentation of recommendations. J Clin Epidemiol. 2013 Jul;66(7):719-25. doi: 10.1016/j.jclinepi.2012.03.013.
7. Santesso N, Glenton C, Dahm P, Garner P, Akl EA, Alper B, et al. GRADE guidelines 26: informative statements to communicate the findings of systematic reviews of interventions. J Clin Epidemiol. 2020 Mar;119:126-135. doi: 10.1016/j.jclinepi.2019.10.014.
