# Supplementary material for: European society for organ transplantation clinical practice guideline on prevention and treatment of chronic lung allograft dysfunction
Source: Transpl Int. 2026 Sep 18;39:16959. doi: 10.3389/ti.2026.16959 (PMC13630708; doi:10.3389/ti.2026.16959)
Supplement: Supplementary file 2 [file DataSheet2.pdf]

European Society for Organ Transplantation Clinical Practice Guideline on Prevention and Treatment of Chronic Lung Allograft Dysfunction: Online Supplement

Supplement 2: Evidence profiles and additional studies

Table of contents

PICO 1 2
PICO 2 6
PICO 3 15
PICO 4 19
PICO 5 27
PICO 6 30
PICO 7 32
PICO 8 35

Evidence profiles and additional studies

PICO 1

Question: In adult lung transplant recipients, should azithromycin be used to prevent the onset of CLAD?

Setting: In- and outpatient, post lung-transplantation

Bibliography:

1. Vos, R.; Vanaudenaerde, B. M.; Verleden, S. E.; et al. A randomised controlled trial of azithromycin to prevent chronic rejection after lung transplantation. Eur Respir J 2011;37(1):164-72.
2. Van Herck, A.; Frick, A. E.; Schaevers, V.; et al. Azithromycin and early allograft function after lung transplantation: A randomized, controlled trial. J Heart Lung Transplant 2019;38(3):252-9.

Certainty assessment							№ of patients		Effect		Certainty	Importance
№ of studies	Study design	Risk of bias	Inconsistency	Indirectness	Imprecision	Other considerations	Azitromycin		Relative (95% CI)	Absolute (95% CI)		
**Graft loss due to CLAD (follow-up: range 2 years to 9 years)**												
1[1]	randomised trials	serious[a]	not serious	not serious	very serious[b]	none	7/40 (17.5%)	14/43 (32.6%)	**RR 0.54** (0.24 to 1.19)	**150 fewer per 1.000** (from 247 fewer to 62 more)	⊕◯◯◯ Very low[a,b]	CRITICAL
**Overall survival (follow-up: median 2 years)**												
2[1,2]	randomised trials	serious[a]	not serious	not serious	very serious[b]	none	10/74 (13.5%)	14/77 (18.2%)	**RR 0.75** (0.35 to 1.57)	**45 fewer per 1.000** (from 118 fewer to 104 more)	⊕◯◯◯ Very low[a,b]	CRITICAL
**CLAD (follow-up: range 2 years to 9 years)**												
2[1,2]	randomised trials	not serious	not serious	not serious	serious[c]	none	22/43 (51%) patients of the placebo group and 11/40 (28%) patients of the long-term azithromycin group developed CLAD (RR 0.54 [0.30-0.96]) during 9 years follow-up. CLAD-free survival was significantly longer in the azithromycin group (p = 0.024). Short term early (once before LTx and until 31 days post-transplantation) azithromycin did not result in difference in CLAD onset at 2 years after transplantation.				⊕⊕⊕◯ Moderate[c]	CRITICAL
**Serious adverse events**												
-	-	-	-	-	-	-	-				-	CRITICAL

Certainty assessment							№ of patients		Effect		Certainty	Importance
№ of studies	Study design	Risk of bias	Inconsistency	Indirectness	Imprecision	Other considerations	Azitromycin		Relative (95% CI)	Absolute (95% CI)		
**Quality of life**												
-	-	-	-	-	-	-	-				-	IMPORTANT
**Infections**												
-	-	-	-	-	-	-	-				-	IMPORTANT
**Kidney function**												
-	-	-	-	-	-	-	-				-	IMPORTANT
**Malignancies**												
-	-	-	-	-	-	-	-				-	IMPORTANT

GRADE Working Group grades of evidence
High certainty: We are very confident that the true effect lies close to that of the estimate of the effect.
Moderate certainty: We are moderately confident in the effect estimate: The true effect is likely to be close to the estimate of the effect, but there is a possibility that it is substantially different.
Low certainty: Our confidence in the effect estimate is limited: The true effect may be substantially different from the estimate of the effect.
Very low certainty: We have very little confidence in the effect estimate: The true effect is likely to be substantially different from the estimate of effect.

Explanations
CI: confidence interval; RR: risk ratio.
a. Downgraded due to overall risk of bias for this outcome, related to deviation from the intended intervention upon development of CLAD.
b. Lack of statistical power to detect a clinically meaningful effect (studies not powered for this outcome).
c. Downgraded one level due to single study with relatively small number of events.

Overall survival meta-analysis

Study or Subgroup	Azithromycin Events	Azithromycin Total	Placebo Events	Placebo Total	Weight	Risk ratio M-H, Fixed, 95% CI	A	B	C	D	E	F	G
Van Herck 2018	4	34	6	34	43.8%	0.67 [0.21 , 2.15]	+	+	+	+	+	+	−
Vos 2011	6	40	8	43	56.2%	0.81 [0.31 , 2.12]	+	+	+	+	+	+	−
**Total**		**74**		**77**	**100.0%**	**0.75 [0.35 , 1.57]**							
Total events:	10		14										

Risk ratio M-H, Fixed, 95% CI

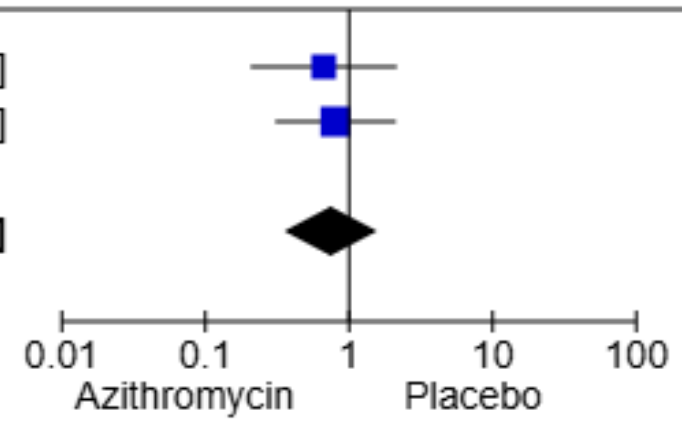

Risk of Bias

Test for overall effect: Z = 0.77 (P = 0.44)
Test for subgroup differences: Not applicable
Heterogeneity: $Chi^2 = 0.06$, df = 1 (P = 0.81); $I^2 = 0\%$

Risk of bias legend
(A) Random sequence generation (selection bias)
(B) Allocation concealment (selection bias)
(C) Blinding of participants and personnel (performance bias)
(D) Blinding of outcome assessment (detection bias)
(E) Incomplete outcome data (attrition bias)
(F) Selective reporting (reporting bias)
(G) Other bias

Additional studies

Study	Study Design	Number of patients	Study Summary	Summary of Relevant Outcomes
Li et al, 2020[1]	Retrospective, single-center	445 BLTR 2004-2016	- Azithromycin prophylaxis 250mg every other day upon medical stability and medication tolerance, initiated prior to CLAD onset (n=344 (77%) after median 51 [IQR] 20-211 days).<br>- Difference between prophylaxis and non-prophylaxis group existed; the prophylaxis group more often had LTR after 2010, IL-2RA induction therapy (versus anti-lymphocyte antibody), shorter duration of ventilation and hospital stay, without difference in PGD. All factors were adjusted for in multivariate cox proportional hazards models.	- Azithromycin prophylaxis was associated with improved survival (HR: 0.59; 95% CI: 0.42-0.82; p=0.0020).<br>- Azithromycin was not associated with clear reduction in the adjusted risk of CLAD (HR: 0.69; 95% CI: 0.47-1.03; p=0.0697).<br>- Causes of death were not different between groups, specifically not related to cardiovascular events.
Porras et al, 2022[2]	Retrospective, single-center	358 LTR 2011-2020	- Azithromycin prophylaxis 250mg 3x per week<br>- Group 1 (n=139) azithromycin prophylaxis started week 3 post-LT, group 2 (n=94) azithromycin prophylaxis started later than 3 weeks post-LT, group 3 (n=91) no azithromycin prophylaxis and group 4 (n=34) started azithromycin treatment (prior formal CLAD onset) due to a decline in FEV1.<br>- SLT was more prevalent in group 3, group 1 more often received induction and group 1 more often had PGD.	- Azithromycin prophylaxis from week 3 post-LT was associated with decreased CLAD incidence in multivariate Cox proportional hazards analysis (HR: 0.24; CI: 0.11-0.55; p=0.001)<br>- Azithromycin prophylaxis started later than week 3 post-LT, but prior FEV1 decline, was associated with decreased CLAD incidence in multivariate Cox proportional hazards analysis (HR: 0.35; CI: 0.17-0.69; p=0.002).

[1] Li, D.; Duan, Q.; Weinkauf, J.; et al. Azithromycin prophylaxis after lung transplantation is associated with improved overall survival. J Heart Lung Transplant 2020;39(12):1426-34.
[2] Cristeto Porras, M.; Mora Cuesta, V. M.; Iturbe Fernandez, D.; et al. Early onset of azithromycin to prevent CLAD in lung transplantation: Promising results of a retrospective single centre experience. Clin Transplant 2023;37(1):e1483 -2023.

PICO 2

Question: In adult lung transplant recipients, should tacrolimus be used over cyclosporine to prevent the onset of CLAD?

Setting: In- and outpatient, post lung-transplantation

Bibliography:

1. Zuckermann A, Reichenspurner H, Birsan T, et al. Cyclosporine A versus tacrolimus in combination with mycophenolate mofetil and steroids as primary immunosuppression after lung transplantation: one-year results of a 2-center prospective randomized trial. J Thorac Cardiovasc Surg. 2003;125(4):891-900.
2. Hachem RR, Yusen RD, Chakinala MM, et al. A randomized controlled trial of tacrolimus versus cyclosporine after lung transplantation. J Heart Lung Transplant. 2007;26(10):1012-8.
3. Treede H, Glanville AR, Klepetko W, et al. Tacrolimus and cyclosporine have differential effects on the risk of development of bronchiolitis obliterans syndrome: results of a prospective, randomized international trial in lung transplantation. J Heart Lung Transplant. 2012;31(8):797-804.
4. Dellgren G, Lund TK, Raivio P, et al. Effect of once-per-day tacrolimus versus twice-per-day ciclosporin on 3-year incidence of chronic lung allograft dysfunction after lung transplantation in Scandinavia (ScanCLAD): a multicentre randomised controlled trial. Lancet Respir Med. 2024;12(1):34-44.

Certainty assessment							№ of patients		Effect		Certainty	Importance
№ of studies	Study design	Risk of bias	Inconsistency	Indirectness	Imprecision	Other considerations	Tacrolimus	Cyclosporin	Relative (95% CI)	Absolute (95% CI)		
**Graft loss due to CLAD (follow-up: range 507 days to 3 years)**												
3[1,3,4]	randomised trials	serious[a]	not serious	not serious	serious[b]	none	29/286 (10.1%)	42/287 (14.6%)	**RR 0.70** (0.47 to 1.06)	**44 fewer per 1.000** (from 78 fewer to 9 more)	⨁⨁◯◯ Low[a,b]	CRITICAL
**Overall survival (follow-up: range 507 days to 3 years)**												
4[1-4]	randomised trials	serious[a]	not serious	not serious	serious[b]	none	69/333 (20.7%)	75/329 (22.8%)	**RR 0.91** (0.68 to 1.21)	**21 fewer per 1.000** (from 73 fewer to 48 more)	⨁⨁◯◯ Low[a,b]	CRITICAL
**CLAD (follow-up: range 507 days to 3 years)**												
4[1-4]	randomised trials	serious[a]	not serious	not serious	not serious	none	38/329 (11.6%)	96/334 (28.7%)	**RR 0.40** (0.28 to 0.57)	**172 fewer per 1.000** (from 207 fewer to 124 fewer)	⨁⨁⨁◯ Moderate[a]	CRITICAL

Certainty assessment							№ of patients		Effect		Certainty	Importance
№ of studies	Study design	Risk of bias	Inconsistency	Indirectness	Imprecision	Other considerations	Tacrolimus	Cyclosporin	Relative (95% CI)	Absolute (95% CI)		
**Serious adverse events (follow-up: mean 3 years)**												
1[4]	randomised trials	not serious	not serious	not serious	not serious	none	No difference in serious adverse events was detected (494 SAE in the TAC group, versus 434 in the CYC group during 3 years, P=0.40). There were also no differences in subcategories of SAE. The majority of SAE comprised hospital admissions or prolongation of hospital stay.				⊕⊕⊕⊕ High	CRITICAL
**Quality of life**												
-	-	-	-	-	-	-	-				-	IMPORTANT
**Infections (follow-up: range 507 days to 3 years)**												
4[1-4]	randomised trials	serious[a]	serious[c]	not serious	serious[d]	none	In pooled data from 3 studies the number of infections/100 patients days was 87/205 in the TAC group, versus 94/208 in the CYC group, RR 0.94 [0.76-1.16]. In 1 large study there were no differences in number of treated infections (111/124 (90%) in the TAC group, versus 113/125 (90%) in the CYC group. There were also no differences with regards to subtypes of first treated infection.				⊕◯◯◯ Very low[a,c,d]	IMPORTANT
**Kidney dysfunction (follow-up: range 507 days to 3 years)**												
4[1-4]	randomised trials	serious[a]	serious[e]	not serious	serious[f]	none	In pooled data from 4 studies the incidence of kidney dysfunction (serum creatinine > 2mg/mL) was 132/329 in the TAC group, versus 103/333 in the CYC group, RR 1.29 [1.08-1.55]. In one other study (N=50) there was no notable difference in extent of renal impairment between groups.				⊕◯◯◯ Very low[a,e,f]	IMPORTANT
**Malignancies (follow-up: range 507 days to 3 years)**												
3[1,2,4]	randomised trials	serious[a]	serious[g]	not serious	very serious[f]	none	21/207 (10.1%)	19/206 (9.2%)	**RR 1.10** (0.62 to 1.97)	**9 more per 1.000** (from 35 fewer to 89 more)	⊕◯◯◯ Very low[a,f,g]	IMPORTANT

GRADE Working Group grades of evidence
High certainty: We are very confident that the true effect lies close to that of the estimate of the effect.
Moderate certainty: We are moderately confident in the effect estimate: The true effect is likely to be close to the estimate of the effect, but there is a possibility that it is substantially different.
Low certainty: Our confidence in the effect estimate is limited: The true effect may be substantially different from the estimate of the effect.
Very low certainty: We have very little confidence in the effect estimate: The true effect is likely to be substantially different from the estimate of effect.

Explanations
CI: confidence interval; RR: risk ratio.

a. Downgraded due to overall risk of bias, mostly related to unclear randomization policies and crossover in treatment allocation.
b. Lack of statistical power to detect a clinically meaningful effect (studies not powered for this outcome).
c. Variation in methodologies across studies contributing to inconsistency (considerable heterogeneity).
d. Results vary significantly across studies without a clear explanation.
e. Not able to pool all outcome due to different kidny dysfunction definitions, heterogeneity in results.
f. Number of events insufficient to detect a meaningful effect.
g. Moderate heterogeneity.

Figure 1. Meta-analysis graft loss

Study or Subgroup	Tacrolimus Events	Tacrolimus Total	Cyclosporin Events	Cyclosporin Total	Weight	Risk ratio IV, Fixed, 95% CI	A	B	C	D	E	F	G
Zuckermann	0	37	2	37	1.9%	0.20 [0.01 , 4.03]	?	?	+	+	+	+	?
Treede	2	124	2	125	4.5%	1.01 [0.14 , 7.04]	?	?	+	+	+	+	−
Dellgren	27	125	38	125	93.6%	0.71 [0.46 , 1.09]	+	+	+	+	+	+	+
**Total**		**286**		**287**	**100.0%**	**0.70 [0.47 , 1.06]**							
Total events:	29		42										

Test for overall effect: Z = 1.66 (P = 0.10)

Heterogeneity: Chi² = 0.81, df = 2 (P = 0.67); I² = 0%

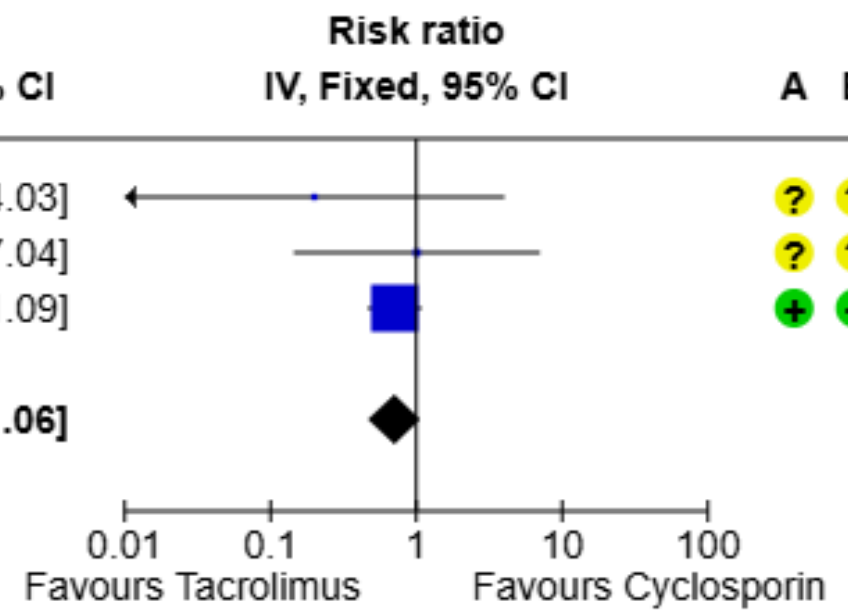

Risk of bias legend

(A) Random sequence generation (selection bias)
(B) Allocation concealment (selection bias)
(C) Blinding of outcome assessment (detection bias)
(D) Blinding of participants and personnel (performance bias)
(E) Incomplete outcome data (attrition bias)
(F) Selective reporting (reporting bias)
(G) Other bias

Figure 2. Meta-analysis survival

Study or Subgroup	Tacrolimus Events	Tacrolimus Total	Cyclosporin Events	Cyclosporin Total	Weight	Risk ratio M-H, Fixed, 95% CI	A	B	C	D	E	F	G
Zuckermann	8	37	11	37	14.6%	0.73 [0.33 , 1.60]	?	?	+	+	+	+	?
Hachem	9	46	11	44	14.9%	0.78 [0.36 , 1.70]	?	?	+	+	+	+	−
Treede	21	125	26	124	34.6%	0.80 [0.48 , 1.35]	?	?	+	+	+	+	−
Dellgren	31	125	27	124	35.9%	1.14 [0.72 , 1.79]	+	+	+	+	+	+	+
**Total**		**333**		**329**	**100.0%**	**0.91 [0.68 , 1.21]**							
Total events:	69		75										

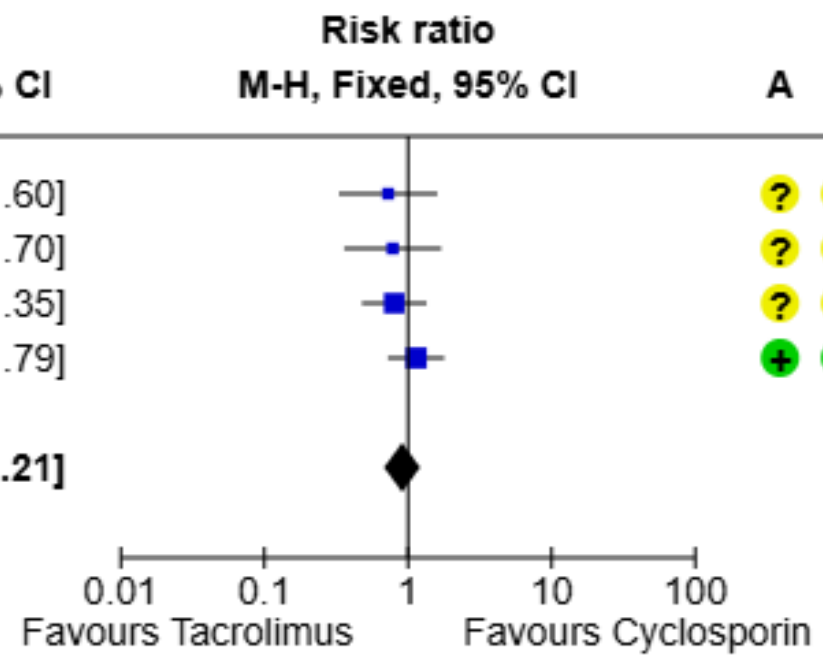

Test for overall effect: Z = 0.65 (P = 0.52)

Heterogeneity: $Chi^2 = 1.63$, df = 3 (P = 0.65); $I^2 = 0\%$

Risk of bias legend

(A) Random sequence generation (selection bias)
(B) Allocation concealment (selection bias)
(C) Blinding of outcome assessment (detection bias)
(D) Blinding of participants and personnel (performance bias)
(E) Incomplete outcome data (attrition bias)
(F) Selective reporting (reporting bias)
(G) Other bias

Figure 3. Meta-analysis CLAD

Study or Subgroup	Tacrolimus Events	Tacrolimus Total	Cyclosporin Events	Cyclosporin Total	Weight	Risk ratio M-H, Fixed, 95% CI	Risk of Bias A	B	C	D	E	F	G
Zuckermann	4	37	15	37	15.7%	0.27 [0.10 , 0.73]	?	?	+	+	+	+	?
Hachem	4	44	6	46	6.2%	0.70 [0.21 , 2.30]	?	?	+	+	+	+	−
Treede	14	124	27	125	28.2%	0.52 [0.29 , 0.95]	?	?	+	+	+	+	−
Dellgren	16	124	48	126	49.9%	0.34 [0.20 , 0.56]	+	+	+	+	+	+	+
**Total**		**329**		**334**	**100.0%**	**0.40 [0.28 , 0.57]**							
Total events:	38		96										

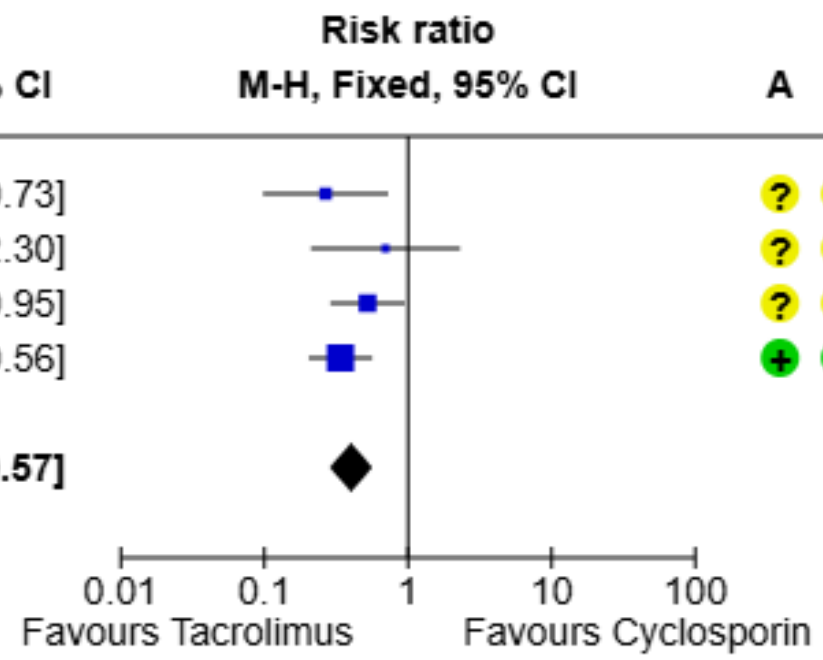

Test for overall effect: Z = 5.23 (P < 0.00001)

Heterogeneity: Chi² = 2.64, df = 3 (P = 0.45); I² = 0%

Risk of bias legend

(A) Random sequence generation (selection bias)
(B) Allocation concealment (selection bias)
(C) Blinding of outcome assessment (detection bias)
(D) Blinding of participants and personnel (performance bias)
(E) Incomplete outcome data (attrition bias)
(F) Selective reporting (reporting bias)
(G) Other bias

Figure 4. Meta-analysis infections/100 patient days

Study or Subgroup	Tacrolimus Events	Tacrolimus Total	Cyclosporin Events	Cyclosporin Total	Weight	Risk ratio M-H, Fixed, 95% CI	Risk of Bias A	B	C	D	E	F	G
Hachem	25	44	26	46	27.3%	1.01 [0.70 , 1.44]	?	?	+	+	+	+	−
Treede	42	124	42	125	44.9%	1.01 [0.71 , 1.43]	?	?	+	+	+	+	−
Zuckermann	20	37	26	37	27.9%	0.77 [0.53 , 1.11]	?	?	+	+	+	+	?
**Total**		**205**		**208**	**100.0%**	**0.94 [0.76 , 1.16]**							
Total events:	87		94										

Test for overall effect: Z = 0.57 (P = 0.57)

Heterogeneity: Chi² = 1.46, df = 2 (P = 0.48); I² = 0%

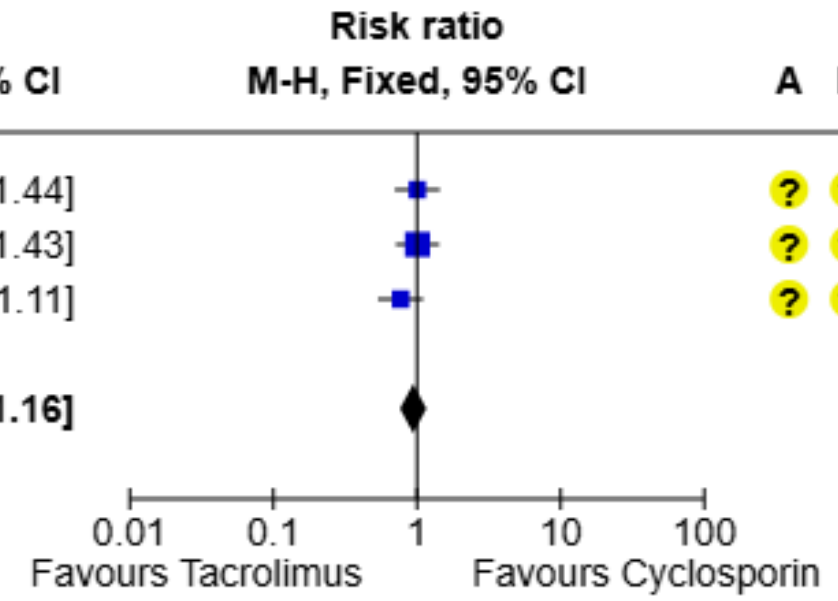

Risk of bias legend

(A) Random sequence generation (selection bias)
(B) Allocation concealment (selection bias)
(C) Blinding of outcome assessment (detection bias)
(D) Blinding of participants and personnel (performance bias)
(E) Incomplete outcome data (attrition bias)
(F) Selective reporting (reporting bias)
(G) Other bias

Figure 5. Meta-analysis kidney dysfunction

Study or Subgroup	Tacrolimus Events	Tacrolimus Total	Cyclosporin Events	Cyclosporin Total	Weight	Risk ratio M-H, Fixed, 95% CI	Risk of Bias A	B	C	D	E	F	G
Dellgren	89	124	72	125	70.0%	1.25 [1.03 , 1.50]	+	+	+	+	+	+	+
Hachem	9	44	6	46	5.7%	1.57 [0.61 , 4.04]	?	?	+	+	+	+	−
Treede	28	124	21	125	20.4%	1.34 [0.81 , 2.23]	?	?	+	+	+	+	−
Zuckermann	6	37	4	37	3.9%	1.50 [0.46 , 4.88]	?	?	+	+	+	+	?
**Total**		**329**		**333**	**100.0%**	**1.29 [1.08 , 1.55]**							
Total events:	132		103										

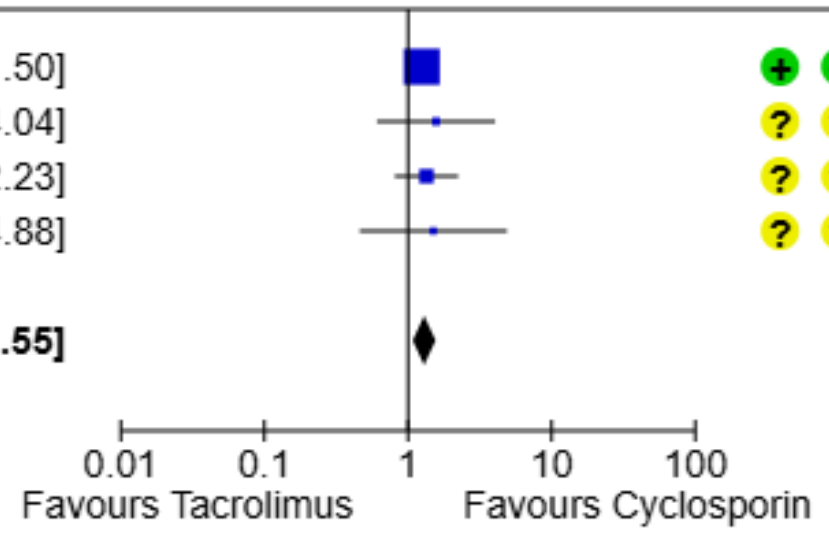

Test for overall effect: Z = 2.76 (P = 0.006)

Heterogeneity: Chi² = 0.40, df = 3 (P = 0.94); I² = 0%

Risk of bias legend

(A) Random sequence generation (selection bias)
(B) Allocation concealment (selection bias)
(C) Blinding of outcome assessment (detection bias)
(D) Blinding of participants and personnel (performance bias)
(E) Incomplete outcome data (attrition bias)
(F) Selective reporting (reporting bias)
(G) Other bias

Figure 6. Meta-analysis malignancies

Study or Subgroup	Tacrolimus Events	Tacrolimus Total	Cyclosporin Events	Cyclosporin Total	Weight	Risk ratio M-H, Fixed, 95% CI	Risk of Bias A	B	C	D	E	F	G
Zuckermann	2	37	0	37	2.6%	5.00 [0.25 , 100.72]	?	?	+	+	+	+	?
Hachem	5	46	1	44	5.3%	4.78 [0.58 , 39.33]	?	?	+	+	+	+	−
Dellgren	14	124	18	125	92.2%	0.78 [0.41 , 1.51]	+	+	+	+	+	+	+
**Total**		**207**		**206**	**100.0%**	**1.10 [0.62 , 1.97]**							
Total events:	21		19										

Test for overall effect: Z = 0.33 (P = 0.74)

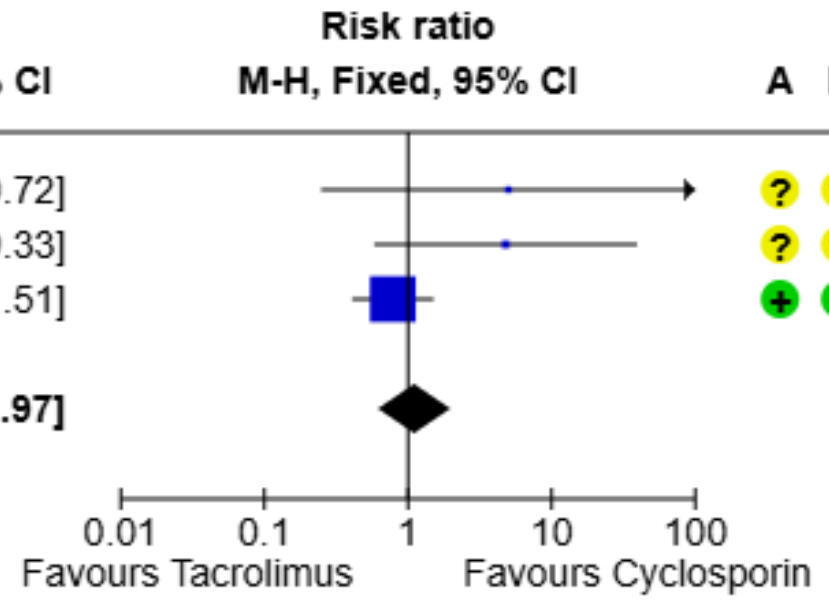

Heterogeneity: $Chi^2 = 3.88$, df = 2 (P = 0.14); $I^2 = 49\%$

Risk of bias legend

(A) Random sequence generation (selection bias)
(B) Allocation concealment (selection bias)
(C) Blinding of outcome assessment (detection bias)
(D) Blinding of participants and personnel (performance bias)
(E) Incomplete outcome data (attrition bias)
(F) Selective reporting (reporting bias)
(G) Other bias

Additional studies

Study	Study Design	Number of patients	Study Summary	Summary of Relevant Outcomes
**Griffith et al, 1994[1]**	Prospective quasi randomised, single-centre	74 LTR<br>1991-1993	- Prospective pseudorandomized clinical trial comparing Tac vs.CsA as primary immunosuppression after isolated lung transplantation, both combined with low-dose steroids and AZA.<br>- Single-lung (n=35) and bilateral lung transplants (n=39).<br>- Groups comparable in age, sex, NYHA class, and underlying disease.	- 1-year survival similar between groups (80% Tac vs 71% CYC; p=NS).<br>- ACR episodes per 100 patient days significantly lower in Tac group (1.2 vs 2.0; p<0.05).<br>- 13% (5/38) of Tac group remained free from ACR within 120 days vs 1/36 (3%) in CYC group (p<0.05).<br>- CYC recipients required more courses of methylprednisolone for ACR (p=0.05).<br>- 34% of Tac recipients were steroid-free vs 6% of CYC recipients (p<0.0001).<br>- Bacterial infection prevalence 1.5 vs 0.6 episodes/100 patient days (CYC vs Tac).<br>- CMV infection symptomatic in 1 Tac vs 5 CYC recipients <120 days.<br>- No gingival hyperplasia or hirsutism in FK group.
**Reichenspurner et al, 1999[3]**	Retrospective, single-center	78 LTR<br>1991-1998	- Group 1 (n=34) rATG induction-pred-aza-CYC, group 2 (n=30) pred-aza-TAC, group 3 (n=12) pred-MMF-TAC.<br>- Groups comparable, apart from follow-up.	- Unadjusted 1-year survival 71% versus 93%, 3-year survival 51% versus 71% in group 1 versus group 2, respectively (p=0.044).<br>- 3 deaths related to CLAD in group 1, 1 in group 2.<br>- There was no difference in the incidence of infections between groups.<br>- At 1-year after the transplant the mean creatinine levels were comparable between group 1 and 2. The incidence of renal dysfunction was 18% (n=6) in group 1 and 13% (n=4) in group 2.<br>- The incidence of new-onset diabetes after transplantation was 27% (n=8) in group 2 and 15% (n=5) in group 1, p=0.04.
**Keenan et al, 1995[2]**	Prospective quasi randomized study, single-center	133 LTR<br>1991-1994	- Recipients assigned in alternate fashion to group 1 or 2.<br>- Group 1 (n=67) pred-aza-CYC, Group 2 nN=66) pred-aza-TAC<br>- Follow-up between 254-1155 days<br>- Cross-over from group 1 to group 2 based on medical grounds was possible (i.e., upon development of CLAD).	- Overall survival was 83% at 1 year and 76% at 2 years in group 2 and 71% and 66% in group 1.<br>- The number of rejection episodes/100 patient days was borderline lower in group 2 (p=0.07).<br>- Of patients surviving at least 2 years, 13/60 (22%) patients developed CLAD in group 2 versus 19/50 (38%) in group 1, p=0.025.<br>- The overall rate of infection was similar in both groups.<br>- Recipient acceptance of TAC was better than CYC based on side effects.
**Treede et al, 2001[4]**	Prospective quasi randomized study	50 LTR<br>1997-1999	- Prospective quasi randomized trial comparing tacrolimus + MMF + steroids vs. cyclosporine + MMF + steroids after lung transplantation.<br>- All patients received induction with rabbit ATG for 3 days.<br>- Single (46%) and double (54%) lung TX.<br>- Median follow-up: 525 days (Tac) vs 511 days (CYC).<br>- Groups comparable in age, sex, diagnosis, and transplant procedure.	- 6-month and 1-year survival similar (Tac: 84.6%/73.1% vs CYC: 83.3%/79.2%; p=NS).<br>- Freedom from acute rejection at 6 months and 1 year slightly higher in Tac group (57.7%/50% vs 45.8%/33.3%; p=NS).<br>- AR episodes per 100 patient days significantly lower in Tac group (0.225 vs 0.426; p<0.05).4 patients in CYC group required switch to Tac for ongoing rejection; no switches in opposite direction.<br>- Trend toward more fungal infections in Tac group (n=7 vs n=1; p=NS).<br>- New-onset diabetes mellitus in 3 Tac patients (11.5%) vs none in CYC group. Greater need for antihypertensives and statins in CYC group.

**Slebos et al, 2005[5]**	Retrospective, single-center	51 LTR<br>1990 onwards	- Group 1 (n=30) rATG induction-pred-aza-CYC and group 2 (n=17) IL-2RA induction-pred-MMF-TAC, consecutive in time.<br>- Groups comparable, apart from follow-up.	- Survival not different, trend favoring group 2.<br>- CLAD-free survival not different (p=0.07), trend favoring group 2.
**Erdman et al, 2022[6]**	Retrospective, national multicentre registry	25355 LTR<br>1999-2017	- Group 1 (n=15478) TAC+MMF, group 2 (n=4263) TAC+aza, group 3 (n=1219) CYC+MMF, group 4 (n=1959) CYC+aza, with important temporal between-group differences.	- Group 3 had significantly increased risk of death or graft failure compared to group 1 in multivariate Cox proportional hazards analysis (HR: 1.30; CI: 1.09-1.56; p=0.004).
**Combs et al, 2025[7]**	Retrospective cohort, international multicentre registry (ISHLT TTX Registry)	22,222 LTR with CNI + CLAD data (19,698 Tac immediate release (IR); 2,477 CsA; 47 Tac extended release (XR))<br>2000-2018	- Retrospective cohort/ registry study evaluating the impact of maintenance calcineurin inhibitor (CNI) regimen (captured at post-transplant discharge) on CLAD incidence and allograft survival.<br>- Primary outcomes: time to CLAD development (with death/retransplantation as competing risk) and time to death/retransplant.<br>- CYC group had proportionally more COPD diagnoses, single lung transplants, transplants prior to 2010, and azathioprine use, reflecting temporal trends.	- CYC vs TAC IR associated with increased risk of CLAD development (HR 1.16, 95% CI 1.08–1.23; p<0.001).<br>- 3-year CLAD incidence: 28.1% (Tac IR) vs 33.0% (CYC).<br>- Cyclosporine vs tacrolimus IR associated with increased risk of death/retransplantation (HR 1.16, 95% CI 1.09–1.23; p<0.001).<br>- 5-year allograft survival: 62.3% (Tac IR) vs 55.5% (CYC).<br>- TAC XR not significantly different from TAC IR for either outcome (limited by small n=47).<br>- CYC-associated increased CLAD risk consistent across multiple subgroups; not superior in any subgroup.<br>- Sensitivity analysis in patients with stable CNI regimens showed larger absolute differences (CLAD HR 1.31; death/retransplant HR 1.36).

[1] Griffith BP, Bando K, Hardesty RL, et al. A prospective randomized trial of FK506 versus cyclosporine after human pulmonary transplantation. Transplantation. 1994;57(6):848-51.

[2] Keenan RJ, Konishi H, Kawai A, et al. Clinical trial of tacrolimus versus cyclosporine in lung transplantation. Ann Thorac Surg. 1995;60(3):580-5.

[3] Reichenspurner H, Kur F, Treede H, et al. Optimization of the immunosuppressive protocol after lung transplantation. Transplantation. 1999;68(1):67-71.

[4] Treede H, Klepetko W, Reichenspurner H, et al. Tacrolimus versus cyclosporine after lung transplantation: a prospective, open, randomized two-center trial comparing two different immunosuppressive protocols. J Heart Lung Transplant. 2001;20(5):511-7.

[5] Slebos DJ, Kauffman HF, Koëter GH, et al. Airway cellular response to two different immunosuppressive regimens in lung transplant recipients. Clin Transplant. 2005;19(2):243-9.

[6] Erdman J, Wolfram J, Nimke D, et al. Lung Transplant Outcomes in Adults in the United States: Retrospective Cohort Study Using Real-world Evidence from the SRTR. Transplantation. 2022;106(6):1233-42.

[7] Combs MP, Walter K, Hixson H, et al. Impact of tacrolimus vs cyclosporine on chronic lung allograft dysfunction incidence and allograft survival in the International Society of Heart and Lung Transplantation registry. J Heart Lung Transplant. 2025;44(3):307-17.

PICO 3

Question: In adult lung transplant recipients, should mycophenolate mofetil be used over azathioprine to prevent the onset of CLAD?

Setting: In- and outpatient, post lung-transplantation

Bibliography:

1. Palmer SM, Baz MA, Sanders L, et al. Results of a randomized, prospective, multicenter trial of mycophenolate mofetil versus azathioprine in the prevention of acute lung allograft rejection. Transplantation. 2001;71(12):1772-6.
2. McNeil K, Glanville AR, Wahlers T, et al. Comparison of mycophenolate mofetil and azathioprine for prevention of bronchiolitis obliterans syndrome in de novo lung transplant recipients. Transplantation. 2006;81(7):998-1003.

Certainty assessment							№ of patients		Effect		Certainty	Importance
№ of studies	Study design	Risk of bias	Inconsistency	Indirectness	Imprecision	Other considerations	MMF	AZA	Relative (95% CI)	Absolute (95% CI)		
**Graft Loss**												
-	-	-	-	-	-	-	-				-	CRITICAL
**Overall survival (follow-up: range 6 months to 3 years)**												
2[1,2]	randomised trials	serious[a]	not serious	not serious	serious[b]	none	45/204 (22.1%)	38/195 (19.5%)	**RR 0.79** (0.57 to 1.11)	**41 fewer per 1.000** (from 84 fewer to 21 more)	⊕⊕◯◯ Low[a,b]	CRITICAL
**CLAD onset (follow-up: median 3 years)**												
1[2]	randomised trials	serious[a]	not serious	not serious	serious[c]	none	43/161 (26.7%)	39/157 (24.8%)	**RR 1.08** (0.74 to 1.56)	**20 more per 1.000** (from 65 fewer to 139 more)	⊕⊕◯◯ Low[a,c]	CRITICAL
**Serious adverse events**												
-	-	-	-	-	-	-	-				-	CRITICAL
**Quality of life**												
-	-	-	-	-	-	-	-				-	IMPORTANT

Certainty assessment							№ of patients		Effect		Certainty	Importance
№ of studies	Study design	Risk of bias	Inconsistency	Indirectness	Imprecision	Other considerations	MMF	AZA	Relative (95% CI)	Absolute (95% CI)		
**Infections (follow-up: range 6 months to 3 years)**												
2[1,2]	randomised trials	serious[a]	not serious	not serious	very serious[b]	none	- Opportunistic infection rates were not different between groups (61% in MMF, versus 67% in AZA, P=NS). Sepsis rates were not different between groups (15% in MMF versus 6% in AZA, P=NS). - Rate of CMV infection and disease were not significantly different between the groups. RR toevoegen				⊕◯◯◯ Very low[a,b]	IMPORTANT
**Kidney function (follow-up: median 3 years)**												
1[2]	randomised trials	serious[a]	not serious	not serious	very serious[b]	none	Kidney dysfunction rates were not different between groups (11% in MMF, versus 16% in AZA, P=NS). RR toevoegen				⊕◯◯◯ Very low[a,b]	IMPORTANT
**Malignancies (follow-up: median 3 years)**												
1[2]	randomised trials	serious[a]	not serious	not serious	very serious[b]	none	Incidence rates of malignancies were not different between groups (12% for MMF, versus 13% for AZA), including post-transplantation lymphoproliferative disorders (MMF 3%, versus 6% for AZA).				⊕◯◯◯ Very low[a,b]	IMPORTANT

GRADE Working Group grades of evidence
High certainty: We are very confident that the true effect lies close to that of the estimate of the effect.
Moderate certainty: We are moderately confident in the effect estimate: The true effect is likely to be close to the estimate of the effect, but there is a possibility that it is substantially different.
Low certainty: Our confidence in the effect estimate is limited: The true effect may be substantially different from the estimate of the effect.
Very low certainty: We have very little confidence in the effect estimate: The true effect is likely to be substantially different from the estimate of effect.

Explanations
CI: confidence interval; RR: risk ratio
a. Downgraded due to overall risk of bias, mostly related to unclear randomization policies and crossover in treatment allocation.
b. Lack of statistical power to detect a clinically meaningful effect (studies not powered for this outcome).
c. Downgraded due to wide confidence intervals crossing the null effect.

Figure 1. Meta- analysis overall survival

Study or Subgroup	MMF Events	MMF Total	AZA Events	AZA Total	Weight	Risk ratio M-H, Fixed, 95% CI	A	B	C	D	E	F	G
Palmer 2001	6	43	7	38	13.0%	0.76 [0.28 , 2.06]	?	?	+	+	+	+	−
McNeil 2006	40	161	49	157	87.0%	0.80 [0.56 , 1.14]	+	+	+	+	+	+	−
**Total**		**204**		**195**	**100.0%**	**0.79 [0.57 , 1.11]**							
Total events:	46		56										

Test for overall effect: Z = 1.37 (P = 0.17)
Test for subgroup differences: Not applicable
Heterogeneity: $Chi^2 = 0.01$, df = 1 (P = 0.93); $I^2 = 0\%$

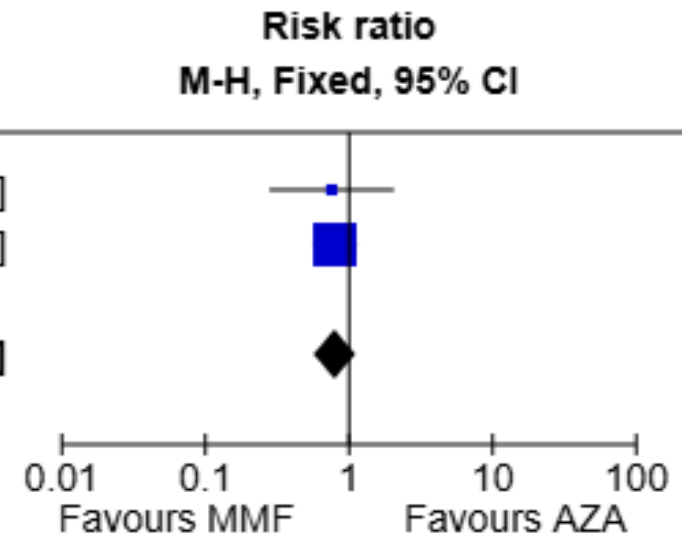

Risk of bias legend

(A) Random sequence generation (selection bias)
(B) Allocation concealment (selection bias)
(C) Blinding of participants and personnel (performance bias)
(D) Blinding of outcome assessment (detection bias)
(E) Incomplete outcome data (attrition bias)
(F) Selective reporting (reporting bias)
(G) Other bias

Additional studies

Study	Study Design	Number of patients	Study Summary	Summary of Relevant Outcomes
**Reichenspurner et al, 1999[1]**	Retrospective, single-center	78 LTR<br>1991-1998	- Group 1 (n=34) rATG induction-pred-aza-CYC, group 2 (n=30) pred-aza-TAC, group 3 (n=12) pred-MMF-TAC.<br>- Groups comparable, apart from follow-up.	- 1 death related to CLAD in group 2, none of the patients group 3 developed CLAD-BOS at mean 5.4 months follow-up.<br>- Follow-up in group 3 minimal, several outcomes not reported for this group, limiting relevant outcome comparisons of group 2 and 3.
**Zuckermann et al, 1999[2]**	Retrospective, single-center	38 LTR<br>1995-1997	- Group 1 (n=21) AZA (ATG induction, other immunosuppression not described), group 2 (n=21) MMF (ATG induction, other immunosuppression not described).<br>- 6 months follow-up.	- Similar numbers of infections per group
**Bhorade et al, 2003[3]**	Retrospective, single-center	109 LTR<br>1997-2000	- Group 1 (n=32) pred-aza-TAC and group 2 (n=49) daclizumab induction-pred-aza-TAC, and group 3 (n=38) daclizumab induction-pred-MMF-TAC.<br>- Groups comparable, apart from follow-up.<br>- Median follow-up for the groups was 66 months for Group 1, 42 months for Group 2 and 36 months for Group 3.	- Three-year survival rates did not differ among the three groups.<br>- Freedom from CLAD-BOS at 3 years did not differ among the three groups, although there appeared to be a trend towards a decreased incidence of CLAD-BOS in Group 3.<br>- The overall incidence of infection up to 3 years after transplantation was comparable among groups.
**Speich et al, 2010[4]**	Retrospective, single-center	156 LTR surviving >60 days<br>1997-2005	- Group 1 (n=48) AZA (other immunosuppression not described), group 2 (n=108) MMF (other immunosuppression not described).<br>- Group 1 more often SLTR and younger donors.<br>- Median follow-up not reported.	- Graft loss was lower in group 2 (p=0.049)<br>- There was a trend towards better survival in group 2 (p=0.062)<br>- Cumulative incidence of CLAD at 5 years was 35% in group 1 versus 41% in group 2, p=NS. Importantly, more than one-third of patients (42%) initially treated with AZA had to be converted to MMF due to either recurrent AR or CLAD-BOS.<br>- CMV infection and disease lower in group 2 compared to group 1 (p<0.001).
**Erdman et al, 2022[5]**	Retrospective, national multicentre registry	25355<br>1999-2017	- Group 1 (n=15478) TAC+MMF, group 2 (n=4263) TAC+aza, group 3 (n=1219) CYC+MMF, group 4 (n=1959) CYC+aza, with important temporal between group-differences.	- Rates of graft loss did not differ between group 1 and 2.<br>- One- and three-year survival rates did not differ between group 1 and 2.<br>- One- and three-year CLAD-BOS incidence did not differ between group 1 and 2.<br>- One- and three-year incidence of hospitalization for infection did not differ between group 1 and 2.<br>- One- and three-year incidence malignancy did not differ between group 1 and 2.

[1] Reichenspurner H, Kur F, Treede H, et al. Optimization of the immunosuppressive protocol after lung transplantation. Transplantation. 1999 Jul 15;68(1):67-71.
[2] Zuckermann A, Klepetko W, Birsan T, et al. Comparison between mycophenolate mofetil- and azathioprine-based immunosuppressions in clinical lung transplantation. J Heart Lung Transplant. 1999;18(5):432-40.
[3] Bhorade SM, Jordan A, Villanueva J, et al. Comparison of three tacrolimus-based immunosuppressive regimens in lung transplantation. Am J Transplant. 2003;3(12):1570-5.
[4] Speich R, Schneider S, Hofer M, et al. Mycophenolate mofetil reduces alveolar inflammation, acute rejection and graft loss due to bronchiolitis obliterans syndrome after lung transplantation. Pulm Pharmacol Ther. 2010;23(5):445-9.
[5] Erdman J, Wolfram J, Nimke D, et al. Lung Transplant Outcomes in Adults in the United States: Retrospective Cohort Study Using Real-world Evidence from the SRTR. Transplantation. 2022;106(6):1233-42.

PICO 4

Question: In adults with (progressive) CLAD, should extracorporeal photopheresis be used?

Setting: In- and outpatient, post lung-transplantation

Bibliography:

1. Salerno CT, Park SJ, Kreykes NS, et al. Adjuvant treatment of refractory lung transplant rejection with extracorporeal photopheresis. J Thorac Cardiovasc Surg. 1999;117(6):1063-9.
2. Jaksch P, Scheed A, Keplinger M, et al. A prospective interventional study on the use of extracorporeal photopheresis in patients with bronchiolitis obliterans syndrome after lung transplantation. J Heart Lung Transplant. 2012;31(9):950-7.
3. Del Fante C, Scudeller L, Oggionni T, et al. Long-Term Off-Line Extracorporeal Photochemotherapy in Patients with Chronic Lung Allograft Rejection Not Responsive to Conventional Treatment: A 10-Year Single-Centre Analysis. Respiration. 2015;90(2):118-28.
4. Pecoraro Y, Carillo C, Diso D, et al. Efficacy of Extracorporeal Photopheresis in Patients With Bronchiolitis Obliterans Syndrome After Lung Transplantation. Transplant Proc. 2017;49(4):695-8.
5. Moniodis A, Townsend K, Rabin A, et al. Comparison of extracorporeal photopheresis and alemtuzumab for the treatment of chronic lung allograft dysfunction. J Heart Lung Transplant. 2018;37(3):340-8.
6. EPI Study Group; Hage CA, Klesney-Tait J, et al. Extracorporeal photopheresis to attenuate decline in lung function due to refractory obstructive allograft dysfunction. Transfus Med. 2021;31(4):292-302.
7. Leroux J, Hirschi S, Essaydi A, et al. Initiation of extracorporeal photopheresis in lung transplant patients with mild to moderate refractory BOS: A single-center real-life experience. Respir Med Res. 2022;81:100913.

Certainty assessment							№ of patients		Effect		Certainty	Importance
№ of studies	Study design	Risk of bias	Inconsistency	Indirectness	Imprecision	Other considerations	ECP	no ECP	Relative (95% CI)	Absolute (95% CI)		
**Graft loss due to CLAD**												
3[2,4,5]	non-randomised studies	very serious[a]	serious[b]	not serious	serious[c]	none	One cohort found higher retransplantation rates in the ECP group than the control group (18/51 (35%) versus 21/143 (15%), p=0.04). Another cohort describes death related to respiratory insufficiency at 1 year of 13/31 (42%) in the ECP group, versus 4/13 (31%) in the observation group (despite 50% cross-over to ECP). Another cohort describes only 1 case of graft loss during followup.				⨁◯◯◯ Very low[a,b,c]	CRITICAL
**Overall survival**												
4[2,4,5,6]	non-randomised studies	very serious[a]	serious[b]	not serious	serious[b,c]	none	No consistent effect was found on survival. One study found that ECP responders (n=31/51) had better survival than no ECP, one study found better survival in ECP compared to no ECP, one study found no difference, one study found worse 1 year survival in ECP group compared to observation.				⨁◯◯◯ Very low[a,b,c]	CRITICAL

Certainty assessment							№ of patients		Effect		Certainty	Importance
№ of studies	Study design	Risk of bias	Inconsistency	Indirectness	Imprecision	Other considerations	ECP	no ECP	Relative (95% CI)	Absolute (95% CI)		
**FEV1**												
5[1-5]	non-randomised studies	very serious[a]	serious[b]	not serious	serious[c]	none	No consistent effect was found on FEV1. Depending on the criteria and time of assessment 61%-90% response rates regarding slope pre- and after ECP are reported. No significant difference in FEV1 slope over time was found in one study compared with no ECP and alemtuzumab. One study found a significantly better FEV1 at 1 year in ECP, and one study found no difference. One study found a comparable >20% drop in FEV1 in ECP and no-ECP.				⊕◯◯◯ Very low[a,b,c]	CRITICAL
**Serious adverse events**												
1[2]	non-randomised studies	very serious[a]	not serious	not serious	serious[c]	none	No serious adverse events reported.				⊕◯◯◯ Very low[a,c]	CRITICAL
**Quality of life**												
-	-	-	-	-	-	-	-	-	-	-	-	IMPORTANT
**Infections**												
3[2,4,5]	non-randomised studies	very serious[a]	serious[b]	not serious	serious[c]	none	82% infection rate was reported in ECP, without comparison to not treated patients. Another study reports 6% sepsis in ECP compared to 0% in no ECP. A third study shows no difference between bacterial/fungal/viral infections between ECP and no-ECP treatment, but describes a significant lower incidence of CMV viremia and CMV disease in the ECP group.				⊕◯◯◯ Very low[a,b,c]	IMPORTANT
**Kidney dysfunction**												
-	-	-	-	-	-	-	-	-	-	-	-	IMPORTANT
**Malignancies**												
-	-	-	-	-	-	-	-	-	-	-	-	IMPORTANT

GRADE Working Group grades of evidence
High certainty: We are very confident that the true effect lies close to that of the estimate of the effect.
Moderate certainty: We are moderately confident in the effect estimate: The true effect is likely to be close to the estimate of the effect, but there is a possibility that it is substantially different.
Low certainty: Our confidence in the effect estimate is limited: The true effect may be substantially different from the estimate of the effect.
Very low certainty: We have very little confidence in the effect estimate: The true effect is likely to be substantially different from the estimate of effect.

Explanations
a. Downgraded two levels due to significant (allocation by indication) bias.
b. Inconsistent results across studies.

c. Sample size insufficient for precise estimates.

Additional studies

Study	Study Design	Number of patients	Study Summary	Summary of Relevant Outcomes
O'Hagan et al, 1999[1]	Retrospective, single-centre	5 LTR 1991-1996	- CLAD stage: not reported.<br>- Time to CLAD post-LTx: mean (+/-SD) 26.8 6 +/- 13.4 months.	- 60% (3/5) FEV1 improved, 20% (1/5) stable, 0% decreased, 20% (1/5) not evaluable.<br>- 60% (3/5) experienced problems with infection while receiving photopheresis: cytomegalovirus in two patients (pneumonia, viremia), P carinii pneumonia, and Aspergillus/Burkholderia sepsis.
Villaneuva et al, 2000[2]	Retrospective, single-centre	14 LTR	- BOS stage: BOS 0'b'/BOS 1/ BOS 2/BOS 3, n= 3/5/3/3.<br>- Time to BOS post-LTx: mean 24 months.<br>- Co-interventions: n=13 were converted to TAC, n=11 were converted to MMF. N=5 underwent TLI. N=5 received MTX. N=3 were treated with ATG. N=4 received plasmapheresis. N=3 received their first ECP treatment + MPS during ACR.	- Mean survival time in months after ECP therapy was 43+/-12 months for patients with early BOS (0b) and BOS 1, and 14+/-10 months for patients with BOS 2 and BOS 3.<br>- Data given for patients with early BOS (0b) and BOS 1 only (n=8): BOS grade worse post-ECP 38% (3/8), BOS grade similar post-ECP 50% (4/8), BOS grade better post-ECP 13% (1/8).<br>- 14% (2/14) developed line-related sepsis.
Meloni et al, 2007[3]	Retrospective, single-centre	8 LTR	- CLAD stage 1/2/3, n=1/3/4.<br>- Time to CLAD post-LTx: mean 20 months post-transplant.	- 75% (6/8) patients provided a minimum follow-up of 4 months, of which 50% (3/6) died following ECP.<br>- Functional stabilization was observed in 60% (3/5) patients with BOS.
Benden et al, 2008[4]	Retrospective, single-centre	12 LTR 1997-2007	- CLAD stage 1/2/3, n= 5/2/5.<br>- Time to CLAD post-LTx: median 29 (range 5–83) months.	- Decline in FEV1 was 112 mL/month before ECP and 12 mL/ month after 12 cycles of ECP, p=0.011, mean (95%CI) change in rate of decline, 100 (range, 28–171).<br>- 14% (2/12) patients were retransplanted because of BOS progression. 33% (4/12) died during the 10-year study related to BOS.<br>- No adverse effects of ECP were recorded.
Isenring et al, 2017[5]		9 LTR +5 years follow-up		- 5-year survival rate 66%, 33% (3/9) death in the CLAD group at year 5 due to CLAD progression (n=1) and cancer (n=2). All patients with CLAD stage 1 survived. 22% (2/9) underwent re-LTx.
Morrell et al, 2010[6]	Retrospective, single-centre	60 LTR 2000-2007	- CLAD stage 1/2/3, n=5/20/35.<br>- Time to CLAD post-LTx: median 615 days.<br>- Co-interventions: 58/60 (97%) patients were maintained on triple-drug immunosuppression, 58 patients (97%) were treated with ATG. Azithromycin was used to treat 54 patients (90%).	- Median survival was 7.5 years after transplantation, 4.7 years after BOS onset, and 2.6 years after ECP initiation.<br>-The rate of decline in lung function was reduced after ECP in 79% (44/56) with FEV1 improved in 25% (14/56).<br>-The 6M average rate of FEV1 decline was −116.0 ml/month, but the slope decreased to −28.9 ml/month.<br>- 13% (8/60) with an indwelling pheresis catheter were hospitalized for line-related sepsis.
Karnes et al, 2019[7]			- To validate the use of FEV1 as a primary outcome and identify factors that are associated with response to ECP.	- Of all covariates analyzed, only baseline FEV1 was linearly related to time to mortality (p=0.007) after ECP initiation, such that 7.7 × [FEV1 in L] + 8.5 = time to death in months. FEV1 just before ECP was associated with mortality (p=0.007) at 16 months after ECP initiation. FEV1 of ≤ 1.50L had a sensitivity of 87% and specificity of 60% to identify patients who died within 16 months after ECP initiation. Patients whose FEV1 decline > 40 mL/month were 12 times more likely to have a response to ECP

				(p=0.0001). Patients whose decline in FEV1 before ECP was statistically significant (p<0.05) were nearly 10 times (p=0.008) more likely to respond to ECP.
**Greer et al, 2013**[8]	Retrospective, single-centre	65 LTR 2007-2011	- CLAD stage 0P/1/2/3, n=3/6/21/35<br>- Time to CLAD post-LTx: median 1114 days (IQR 601–1,994).<br>- Median number of ECP cycles completed was 11 (IQR8–15). N=8 died within the first year of ECP treatment. N=13 in the progressive group terminated ECP due to clinical frailty.	- 12% (8/65) improved, 42% (27/65) stabilised, 46% (30/65) progressed despite ECP.<br>- Time to CLAD diagnosis and initiation of ECP treatment did not influence response. Neither the degree of CLAD, based on BOS stage (p=0.80), nor evident colonization (p=0.48) predicted efficacy. Rapid-decliners were more likely to exhibit a refractory course (n=6 vs. n=15; p=0.005; HR 6.41, 95%CI: 2.77-14.81). Similarly, patients with RAS were less likely to stabilize (n=6 vs. n=16, p=0.002; HR 4.06, 95%CI: 1.84-9.02). Non- neutrophilic patients (n=44, p=0.01) also exhibited worse prognosis.
**Greer et al, 2023**[9]		373 LTR 2007-2022	- Patients were divided in 5 clusters (fulminant, mild, moderate, advanced progressive and advanced chronic) based on FEV1 decline rhythm.<br>- CLAD stage: 35 stage 1 (9%) =mild group; 123 stage 2 (33%) = moderate + fulminant; 215 stage 3 (58%) = advanced groups (chronic + progressive).<br>- Time to CLAD post-LTx: 31 months [18; 60]<br>- Time to intervention post-CLAD: median 9 months [2; 32].<br>- Co-interventions: montelukast was used in 58%, frequently concomitant with ECP (or very near before/after).	- Survival non-responders median 133 [IQR 80-190] days versus 401 [301-576] days in responders.<br><br>- During follow-up n=203 (54%) deaths (CLAD was the cause of death in 57% of cases) and n=19 (5%) re-LTx. Rates of CLAD-associated graft loss were highest in the fulminant group (70%) and lowest in the mild group (42%).
**Chionis et al, 2018**[10]	Retrospective, multicenter	26 LTR<br>2015-2016<br><br>*100% of patients included overlap with Hage et al. 2021*	- CLAD stage 1/2/3, n=13/8/5.<br>- Time to CLAD post-LTx: not reported.<br>- Comparison with n=57 included in Morell et al, 2010.<br>- Intervention: ECP with CELLEX 24 ECP treatment over 6 months, historic control UVAR 24 ECP treatment over 6 months.	- Non-survivors had a significantly higher pre-intervention FEV1 slope -232 vs -126 ml/month. Mortality after ECP was significantly associated with a higher pre-ECP FEV1 slope > 100 ml/month and absolute FEV1 pre-ECP < 1200 mL. Multivariate Cox analysis showed a significant association between survival at M12 under ECP and previous use of ATG (OR 0.61), pre-ECP FEV1 (OR 0.44) and pre-ECP FEV1 slope (OR 0.997).
**Vazirani et al, 2021**[11]	Retrospective, single-centre	12 LTR<br>2013-2018	- CLAD stage 1/2/3, n=0/2/10.<br>- Time to CLAD post-LTx: mean 718 days.<br>- Co-interventions: most patients had TAC+Aza/MMF+pred +azithromycine. All had MPS course. N=8 had ATG.	- 67% (8/12) responded to ECP. Among responders, mean decline in FEV1 pre-ECP was 9.0 mL/day (5-12 mL/day), compared to 1.4 mL/day (0-4 mL/day) post-ECP (p=0.01). Among non-responders, mean decline in FEV1 was 7.2 mL/day (4-10 mL/day) pre-ECP and 5.0 mL/day (3-7 mL/day) post ECP (p=0.2).<br>- All responders had a normal neutrophil count, and 25% of non-responders had a normal neutrophil count prior to commencement of ECP (n=1; p=0.005). Both patients with RAS phenotype who underwent ECP responded to therapy, and both patients with early stage CLAD (stage 2) responded to ECP. Mean difference in FEV1 decline among patients who did not have ATG prior to ECP was 10.9 mL/day (6.6-14 mL/day) vs. 4.9 mL/day (3.4-5.7 mL/day) among those who were exposed to ATG (p=0.02).
**Benazzo et al, 2024**[12]	Retrospective, multicenter	631 LTR<br>1989-2021	- CLAD: BOS/RAS, n=513/78.<br>- Time to CLAD post-LTx: median 34 months (IQR 18–64).<br>- Median number of ECP cycles completed 15 (IQR 11–25).	- 52% (294/568) stable, 10% (43/568) responder, 24% (138/568) non-responder, 14% (79/568) death at 6 months.<br>- 42% (252/605) stable, 9% (55/605) responder, 26% (160/605) non-responder, 23% (138/605) death at 12 months.

		>50% of described patients overlap with Greer et al.	- To investigate the long-term results of the largest cohort of LTR treated with ECP, to identify potential predictors of favourable outcomes.	- Better results of ECP in early CLAD with preserved FEV1, poor results in rapid decliners. RAS was more often associated with non-response.<br>- Observed is a frequent ongoing decrease of FEV1 in the first 6-12 month after ECP before further stabilization (in survivors).
**Gautschi 2024**[13]	Retrospective, single-centre	93 LTR<br>2010-2020	- CLAD stage 1/2/3/4, n=*37(40%)/28(30%)/15(16%)/11(11%).*<br>- CLAD phenotype: *61 BOS (65%), 4 RAS (4%), 13 mixed (14%).*<br>- Time to CLAD post-LTx: Mean 1248±1514 days = 42 months±50 months.<br>- Time to intervention post-CLAD: 584±841 days = 19.4±28 months.<br>- Median follow-up: 25 months after ECP (maximum 5 years after ECP start).<br>- FEV1 response (n=72) was defined as an increase or <10% variation of initial pre-ECP FEV1 ; Non-Response: Death due to CLAD or >10% FEV1 decrease.	- After ECP initiation, patients were followed for 25 months. 61% (57/93) died within the study period at a mean of 15 months after ECP initiation. CLAD was the cause of death in 82% (47/57).<br>- Median decline in FEV1 was 32.7 mL/month before ECP and 4.7 mL/month after 12 cycles of ECP (p<0.001).<br>- Responders: 19% (14/72). Responders were statistically significantly younger, had more stable FEV1 values and better five-year survival compared to non-responders.<br>- Response was lower in patients with CLAD-RAS and mixed subtype and patients with ECP onset in CLAD stages 3 and 4, although these values did not reach statistical significance.<br>- Trend towards better chance of progression free survival in patient with stage 1 CLAD.

[1] O'Hagan AR, Stillwell PC, Arroliga A, et al. Photopheresis in the treatment of refractory bronchiolitis obliterans complicating lung transplantation. Chest. 1999;115(5):1459-62.

[2] Villanueva J, Bhorade SM, Robinson JA, et al. Extracorporeal photopheresis for the treatment of lung allograft rejection. Ann Transplant. 2000;5(3):44-7.

[3] Meloni F, Cascina A, Miserere S, et al. Peripheral CD4(+)CD25(+) TREG cell counts and the response to extracorporeal photopheresis in lung transplant recipients. Transplant Proc. 2007;39(1):213-7.

[4] Benden C, Speich R, Hofbauer GF, et al. Extracorporeal photopheresis after lung transplantation: a 10-year single-center experience. Transplantation. 2008;86(11):1625-7.

[5] Isenring B, Robinson C, Buergi U, et al. Lung transplant recipients on long-term extracorporeal photopheresis. Clin Transplant. 2017;31(10).

[6] Morrell MR, Despotis GJ, Lublin DM, et al. The efficacy of photopheresis for bronchiolitis obliterans syndrome after lung transplantation. J Heart Lung Transplant. 2010;29(4):424-31.

[7] Karnes HE, Schindler EI, Morrell M, et al. Factors Associated With Mortality and Response to Extracorporeal Photopheresis in Lung Allograft Recipients With Bronchiolitis Obliterans Syndrome. Transplantation. 2019;103(5):1036-42.

[8] Greer M, Dierich M, De Wall C, et al. Phenotyping established chronic lung allograft dysfunction predicts extracorporeal photopheresis response in lung transplant patients. Am J Transplant. 2013;13(4):911-8.

[9] Greer M, Liu B, Magnusson JM, et al. Assessing treatment outcomes in CLAD: The Hannover-extracorporeal photopheresis model. J Heart Lung Transplant. 2023;42(2):209-17.

[10] Chionis L, Grossman BJ, Hachem R, et al. The efficacy of extracorporeal photopheresis to arrest bronchiolitis obliterans in lung allograft recipients was compared between two automated photopheresis instruments. Transfusion. 2018;58(12):2933-41.

[11] Vazirani J, Routledge D, Snell GI, et al. Outcomes Following Extracorporeal Photopheresis for Chronic Lung Allograft Dysfunction Following Lung Transplantation: A Single-Center Experience. Transplant Proc. 2021;53(1):296-302.

[12] Benazzo A, Bagnera C, Ius F, et al. A European Multi-Center Analysis of Extracorporeal Photopheresis as Therapy for Chronic Lung Allograft Dysfunction. Transpl Int. 2024;36:11551.

[13] Gautschi F, Vogelmann T, Ortmanns G, et al. Early extracorporeal photopheresis treatment is associated with better survival in patients with chronic or recurrent acute lung allograft dysfunction. J Clin Apher. 2024;39(3):e22128.

PICO 5

Question: In adults with (progressive) CLAD, should anti-thymocyte globulin be used?

Setting: In- and outpatient, post lung-transplantation

Bibliography:

1. Kesten S, Rajagopalan N, Maurer J. Cytolytic therapy for the treatment of bronchiolitis obliterans syndrome following lung transplantation. Transplantation. 1996;61(3):427-30.
2. Snell GI, Esmore DS, Williams TJ. Cytolytic therapy for the bronchiolitis obliterans syndrome complicating lung transplantation. Chest. 1996;109(4):874-8.
3. Date H, Lynch JP, Sundaresan S, Patterson GA, Trulock EP. The impact of cytolytic therapy on bronchiolitis obliterans syndrome. J Heart Lung Transplant. 1998;17(9):869-75.
4. Izhakian S, Wasser WG, Fox BD, et al. Effectiveness of Rabbit Antithymocyte Globulin in Chronic Lung Allograft Dysfunction. Transplant Proc. 2016;48(6):2152-6.
5. January SE, Fester KA, Bain KB, Kulkarni HS, Witt CA, Byers DE, Alexander-Brett J, Trulock EP, Hachem RR. Rabbit antithymocyte globulin for the treatment of chronic lung allograft dysfunction. Clin Transplant. 2019;33(10):e13708.
6. Kotecha S, Paul E, Ivulich S, et al. Outcomes Following ATG Therapy for Chronic Lung Allograft Dysfunction. Transplant Direct. 2021;7(4):e681.
7. Margallo Iribarnegaray J, De Pablo Gafas A, Alonso Moralejo R, et al. Antithymocyte Globulin Treatment for Chronic Lung Allograft Dysfunction in Lung Transplant Recipients: Experience From a National Reference Transplant Center. Transplant Proc. 2021;53(9):2710-2717.
8. Dunn CT, Brandon W, Jacob A, et al. Anti-Thymocyte Globulin as a Therapy for Chronic Lung Allograft Dysfunction: A Single-Center Experience. Clin Transplant. 2024;38(10):e15461.
9. Padhye A, Guffey D, Leon-Pena A, et al. Antithymocyte globulin therapy in chronic lung allograft dysfunction. Front. Transplant. 2025;4:1607678.

Certainty assessment							№ of patients		Effect		Certainty	Importance
№ of studies	Study design	Risk of bias	Inconsistency	Indirectness	Imprecision	Other considerations			Relative (95% CI)	Absolute (95% CI)		
**Graft loss**												
3[2,6,8]	Retrospective observational	Serious[a]	Serious[b]	Not serious	Serious[d]	None	153		Two studies[2,6] reported graft loss of 20% (2/10) at a median follow-up of 310 days and 23% (16/71) with a median follow-up of 63 months. Another study[8] reported a median graft survival of 32 months after CLAD onset (54/72 graft loss). In a propensity-matched subanalysis, median graft survival was 26 months in 50 ATG-treated patients vs. 49 months in 50 non-treated patients (p=0.021).		⊕◯◯◯ Very low	CRITICAL

Overall survival

Certainty assessment							№ of patients		Effect		Certainty	Importance
№ of studies	Study design	Risk of bias	Inconsistency	Indirectness	Imprecision	Other considerations			Relative (95% CI)	Absolute (95% CI)		
5[2,4,5,7,9]	Retrospective observational	Serious[a]	Serious[c]	Not serious	Serious[d]	None	211	69	One study reported a median OS of 27±15 months after ATG administration. Other studies reported OS of 95% (103/108) at 6 months, 90% (9/10) at a median of 310 days and 56% (14/25) with 100% of stabilizers (8/8) being alive (930±385 days after ATG, OS 2318±881) vs. 35% (6/17) decliners (414±277 after ATG, OS 1798±126). One study with a control group reported numerically but not statistically lower 1-year post-CLAD mortality in the ATG-treated group (SHR 0.66, 95%CI 0.39-1.14, p=0.134).[9]		⊕◯◯◯ Very low	CRITICAL
**Serious adverse events**												
-	-	-	-	-	-	-	-		-		-	CRITICAL
**Quality of life**												
-	-	-	-	-	-	-	-		-		-	CRITICAL
**FEV1**												
9[1-9]	Retrospective observational	Not serious	Serious[b]	Not serious	Serious[e]	None	390	68	Between 32% and 52%[1,3-8] of patients responded based on FEV1 improvement (in 13-40%) and stabilization[1,3,4,6,7] or based on FEV1 improvement (in 13% and 40%) and slowing of FEV1 decline > 20% (in 65% and 44%)[5,8]. Further FEV1 decline occurred in 10-68% of patients[1-8]. One study reported no difference in rate of FEV1 decline after vs before ATG (-0,09 L/year, 95%CI -0,21-0,03) and compared with non-treated patients [−0.1481 L/year, 95% CI (−0.32-0.021).[9]		⊕◯◯◯ Very low	CRITICAL
**Malignancies**												
4[1,2,5,8]	Retrospective observational	Serious[a]	Serious[c]	Not serious	Serious[d]	None	205		Two studies[5,8] reported 9% (10/108) and 19% (14/72) of patients developed skin cancer and 8% (9/108) and 7% (5/72) other types of malignancy. Two other studies[1,2] reported on PTLD with an incidence of 0% (0/15) and 10% (1/10).		⊕◯◯◯ Very low	IMPORTANT
**Kidney function**												
-	-	-	-	-	-	-	-		-		-	IMPORTANT

GRADE Working Group grades of evidence
High certainty: We are very confident that the true effect lies close to that of the estimate of the effect.
Moderate certainty: We are moderately confident in the effect estimate: The true effect is likely to be close to the estimate of the effect, but there is a possibility that it is substantially different.
Low certainty: Our confidence in the effect estimate is limited: The true effect may be substantially different from the estimate of the effect.
Very low certainty: We have very little confidence in the effect estimate: The true effect is likely to be substantially different from the estimate of effect.

Explanations

Retrospective single-center studies with 1) 15 BOS patients, 2) 10 BOS patients, 3) 30 BOS patients, 4) 25 CLAD patients, 5) 108 CLAD patients (94% BOS, 6% RAS), 6) 71 CLAD patients (83% BOS, 11% RAS, 6% mixed/undefined), 7) 13 CLAD patients (77% BOS, 23% RAS), 8) 72 CLAD patients (83% BOS, 8% RAS, 9%) with FEV1 data for 63 patients.

a. Downgraded one level due to overall risk of bias due to the selection of participants into the study and too short follow-up time for specific outcomes.

b. Downgraded one level due to clinical and methodological heterogeneity across studies and variability in results.

c. Downgraded one level due to clinical and methodological heterogeneity across studies.

d. Downgraded one level for imprecision due to few studies, participants and/or events.

e. Downgraded one level for imprecision due to uncertainty regarding the estimated effect (wide variation).

PICO 6

Question: In adults with (progressive) CLAD, should alemtuzumab be used?

Setting: In- and outpatient, post lung-transplantation

Bibliography:

1. Reams BD, Musselwhite LW, Zaas DW, Steele MP, Garantziotis S, Eu PC, Snyder LD, Curl J, Lin SS, Davis RD, Palmer SM. Alemtuzumab in the treatment of refractory acute rejection and bronchiolitis obliterans syndrome after human lung transplantation. Am J Transplant. 2007;7(12):2802-8.
2. Ensor CR, Rihtarchik LC, Morrell MR, Hayanga JW, Lichvar AB, Pilewski JM, Wisniewski S, Johnson BA, D'Cunha J, Zeevi A, McDyer JF. Rescue alemtuzumab for refractory acute cellular rejection and bronchiolitis obliterans syndrome after lung transplantation. Clin Transplant. 2017;31(4).
3. Moniodis A, Townsend K, Rabin A, Aloum O, Stempel J, Burkett P, Camp P, Divo M, El-Chemaly S, Mallidi H, Rosas I, Fuhlbrigge A, Koo S, Goldberg HJ. Comparison of extracorporeal photopheresis and alemtuzumab for the treatment of chronic lung allograft dysfunction. J Heart Lung Transplant. 2018;37(3):340-348.

Certainty assessment							№ of patients		Effect		Certainty	Importance
№ of studies	Study design	Risk of bias	Inconsistency	Indirectness	Imprecision	Other considerations			Relative (95% CI)	Absolute (95% CI)		
**Graft loss**												
1[1]	Retrospective observational	Serious[a]	Not serious	Not serious	Serious[b]	Not serious	10		One study[1] reported 31% graft loss at 1 and 2 years.		⊕◯◯◯ Very low	CRITICAL
**Overall survival**												
3[1,2,3]	Retrospective observational	Serious[a]	Serious[c]	Not serious	Serious[b]	Not serious	41	78	One study[3] with a control group reported no difference in 1-year OS between alemtuzumab (0.92 (95%CI 0.57-0.99)) and the non-treated group (0.81 (95%CI 0.70-0.88), p=0.32). The other studies reported an overall survival of 69% at 1 and 2 years[1] and 76% at 5 years[2].		⊕◯◯◯ Very low	CRITICAL
**Serious adverse events**												
-	-	-	-	-	-	-	-		-		-	CRITICAL
**Quality of life**												
-	-	-	-	-	-	-	-		-		-	CRITICAL
**FEV1**												

Certainty assessment							№ of patients		Effect		Certainty	Importance
№ of studies	Study design	Risk of bias	Inconsistency	Indirectness	Imprecision	Other considerations			Relative (95% CI)	Absolute (95% CI)		
3[1,2,3]	Retrospective observational	Serious[d]	Not serious	Not serious	Serious[b]	Not serious	41	78	One study[3] showed a significantly slower FEV1 decline post-treatment at 1 month, 3 months and 6 months vs. pre-treatment in CLAD patients. At 1 month, patients treated with alemtuzumab had a significantly better FEV1 slope compared with non-treated patients, this was no longer the case at 3 and 6 months. CLAD patients had a significantly steeper decline in FEV1 pre-treatment compared with non-treated group (-131 mL/month vs. -6 mL/month). Another study[1] showed 40% improvement, 30% stabilization and 30% decline of FEV1, with no significant change in FEV1 overall. The last study[2] showed 53% freedom from BOS progression at 180 days.		⨁◯◯◯ Very low	CRITICAL
**Malignancies**												
-	-	-	-	-	-	-	-		-		-	IMPORTANT
**Kidney function**												
-	-	-	-	-	-	-	-		-		-	IMPORTANT

GRADE Working Group grades of evidence
High certainty: We are very confident that the true effect lies close to that of the estimate of the effect.
Moderate certainty: We are moderately confident in the effect estimate: The true effect is likely to be close to the estimate of the effect, but there is a possibility that it is substantially different.
Low certainty: Our confidence in the effect estimate is limited: The true effect may be substantially different from the estimate of the effect.
Very low certainty: We have very little confidence in the effect estimate: The true effect is likely to be substantially different from the estimate of effect.

Explanations

1) Retrospective single-center study with 10 BOS patients, 2) retrospective single-center study with 17 BOS patients, 3) retrospective single-center study with 14 CLAD patients (9 BOS, 5 RAS) and 78 non-treated patients.

a. Downgraded one level due to overall risk of bias due to the selection of participants into the study and too short follow-up time.
b. Downgraded one level for imprecision due to few studies and participants.
c. Downgraded one level for inconsistency due to inconsistency in the results with different effect estimates.
d. Downgraded one level due to overall risk of bias due to the selection of participants into the study.

PICO 7

Question: In adults with CLAD, should antifibrotics be used?

Setting: In- and outpatient, post lung-transplantation

Bibliography:

1. Combs MP, Belloli EA, Gargurevich N, et al. Results from randomized trial of pirfenidone in patients with chronic rejection (STOP-CLAD study). J Heart Lung Transplant. 2024;43(9):1468-77.
2. Perch M, Corris P, Lordan J, et al. A European multi-center, randomized controlled trial of Pirfenidone in bronchiolitis obliterans syndrome after bilateral lung transplantation. European Respiratory Journal. 2025;2402154.

Certainty assessment							№ of patients		Effect		Certainty	Importance
№ of studies	Study design	Risk of bias	Inconsistency	Indirectness	Imprecision	Other considerations			Relative (95% CI)	Absolute (95% CI)		
**Graft loss**												
1[2]	RCT	Serious[a]	Not serious	Not serious	Serious[b]	None	48	42	4 deaths/retransplants in the treatment group vs. 2 in the placebo group.		⊕⊕◯◯ Low	CRITICAL
**Overall survival**												
1[2]	RCT	Serious[a]	Not serious	Not serious	Serious[b]	None	48	42	3 deaths in the treatment group vs. 1 in the placebo group.		⊕⊕◯◯ Low	CRITICAL
**Serious adverse events**												
2[1,2]	RCT	Serious[a]	Not serious	Not serious	Serious[b]	None	60	53	In the placebo group were 5 events, in the treatment group 0 in the study by Combs et al. In the other study, there was no significant difference between groups (220 adverse events in 45 (93.8%) patients in the pirfenidone group vs. 152 events in 37 (88.1%) controls, p=0.4654).		⊕⊕◯◯ Low	CRITICAL
**Quality of life**												
-	-	-	-	-	-	-	-		-		-	CRITICAL
**FEV1**												
2[1,2]	RCT	Serious[a]	Not serious	Not serious	Serious[c]	None	42	39	No significant differences in rate of FEV1 decline in the treatment group vs. the placebo group.		⊕⊕◯◯ Low	CRITICAL
**Malignancies**												
-	-	-	-	-	-	-	-		-		-	IMPORTANT

Certainty assessment							№ of patients		Effect		Certainty	Importance
№ of studies	Study design	Risk of bias	Inconsistency	Indirectness	Imprecision	Other considerations			Relative (95% CI)	Absolute (95% CI)		
**Kidney function**												
-	-	-	-	-	-	-	-		-		-	IMPORTANT

GRADE Working Group grades of evidence
High certainty: We are very confident that the true effect lies close to that of the estimate of the effect.
Moderate certainty: We are moderately confident in the effect estimate: The true effect is likely to be close to the estimate of the effect, but there is a possibility that it is substantially different.
Low certainty: Our confidence in the effect estimate is limited: The true effect may be substantially different from the estimate of the effect.
Very low certainty: We have very little confidence in the effect estimate: The true effect is likely to be substantially different from the estimate of effect.

Explanations
1) Single-center randomized controlled trial (pirfenidone vs. placebo in CLAD: BOS 7/7, RAS 5/4), stopped prematurely due to COVID-19 pandemic with enrollment of 23 out of planned 60 patients.
a. Downgraded one level due to overall risk of bias arising from the randomization process and deviations from the intended intervention.
b. Downgraded one level for imprecision due to few studies and participants and low number of events.
c. Downgraded one level for imprecision due to few studies and participants.

Additional studies

Study	Study Design	Number of patients	Study Summary	Summary of Relevant Outcomes
Vos et al., 2018[1]	Prospective, single-center cohort study	n=11	- Pirfenidone 801 mg TID, treated for at least 3 months<br>- RAS<br>- Median treatment duration 12 (5-19) months<br>- Median time of follow-up post-transplant 84 (56-128) months	- 73% (8/11) graft loss, median graft loss-free survival 1.8 years after RAS diagnosis.<br>- 45% (5/11) died at a median of 14 (10-21) months post-pirfenidone initiation.<br>- 27% (3/11) had a re-transplant at 11 (5-12) months post-pirfenidone initiation.<br>- Median 3-year OS 55% after RAS diagnosis.<br>- FEV1 decline reduced from 50 (28-101) 12 months pre-pirfenidone to 10 (-11-47) mL/month 12 months after pirfenidone initiation (p<0.05) in 6/11 patients with lung function data, no significant difference at 3 and 6 months.
Bennett et al., 2020[2]	Retrospective, single-center cohort study	n=9	- Pirfenidone 267 mg 3 capsules TID<br>- CLAD: 5 BOS, 2 RAS, 2 mixed<br>- Mean treatment duration 409±535 days<br>- FEV1 6 months before and after treatment (only data for 3 patients; 3 died before month 6, 2 insufficient data, 1 discontinued therapy and 1 had not reached month 6 yet)	- 33% (3/9) died during first 6 months, 67% (6/9) died during follow-up.<br>- Median OS of 686 days after CLAD diagnosis, 221 days from start of treatment.<br>- FEV1 decline reduced from -45±41 to -13±34 mL/month in 3/9 patients with lung function data at 6 months (p=0.874). (2 stable FEV1 (1 BOS, 1 RAS), 1 decline (RAS)), no difference in FEV1 at 3 months (-50±42 in 6/9 patients with data).

[1] Vos R, Wuyts WA, Gheysens O, et al. Pirfenidone in restrictive allograft syndrome after lung transplantation: A case series. Am J Transplant. 2018;18(12):3045-59.

[2] Bennett D, Lanzarone N, Fossi A, et al. Pirfenidone in chronic lung allograft dysfunction: a single cohort study. Panminerva Med. 2020;62(3):143-9.

PICO 8

Question: In adults with (progressive) CLAD, should montelukast be used?

Setting: In- and outpatient, post lung-transplantation

Bibliography:

1. Ruttens D, Verleden SE, Demeyer H, et al. Montelukast for bronchiolitis obliterans syndrome after lung transplantation: A randomized controlled trial. PLoS One. 2018;13(4):e0193564.

Certainty assessment							№ of patients		Effect		Certainty	Importance
№ of studies	Study design	Risk of bias	Inconsistency	Indirectness	Imprecision	Other considerations	Treatment	Placebo	Relative risk (95% CI)	Anticipated absolute risk (95% CI)*		
**Graft loss**												
- **Graft loss at 1 year**												
1[1]	RCT	Not serious	Not serious	Not serious	Very serious[a]	None	1/15 (7%)	1/15 (7%)	RR 1.00 (0.07-14.55) (p=1.00)	0 fewer per 1.000 (from 62 fewer to 903 more)	⊕⊕◯◯ Low	CRITICAL
									Graft loss was similar between both groups at 1 year (log-rank p=0.981).			
- **Graft loss at 2 years**												
1[1]	RCT	Not serious	Not serious	Not serious	Very serious[a]	None	2/15 (13%)	10/15 (33%)	RR 0.40 (0.09-1.75) (p=0.22)	200 fewer per 1.000 (from 303 fewer to 250 more)	⊕⊕◯◯ Low	CRITICAL
									Graft loss was similar between both groups at 2 years (log-rank p=0.230).			
**Overall survival****												
- **Overall survival at 1 year**												
1[1]	RCT	Not serious	Not serious	Not serious	Very serious[a]	None	1/15 (7%)	1/15 (7%)	RR 1.0 (0.07-14.6) (p=1.00)	0 fewer per 1.000 (from 62 fewer to 903 more)	⊕⊕◯◯ Low	CRITICAL
									Overall survival was similar between both groups at 1 year (log-rank p=0.981).			
- **Overall survival at 2 years**												
1[1]	RCT	Not serious	Not serious	Not serious	Very serious[a]	None	2/15 (13%)	10/15 (33%)	RR 0.40 (0.09-1.75) (p=0.22)	200 fewer per 1.000 (from 303 fewer to 250 more)	⊕⊕◯◯ Low	CRITICAL
									Overall survival was similar between both groups at 2 years (log-rank p=0.230).			
**Serious adverse events*****												
-	-	-	-	-	-	-	-		-		-	CRITICAL

Certainty assessment							№ of patients		Effect		Certainty	Importance
№ of studies	Study design	Risk of bias	Inconsistency	Indirectness	Imprecision	Other considerations	Treatment	Placebo	Relative risk (95% CI)	Anticipated absolute risk (95% CI)*		
**Quality of life**												
-	-	-	-	-	-	-	-		-		-	IMPORTANT
**FEV1 (relative and absolute risk at 6 months for FEV1 decline)**												
1[1]	RCT	Not serious	Not serious	Not serious	Very serious[a]	None	5/15 (33%)	6/15 (40%)	RR 0.83 (0.32-2.15) (p=0.71)	68 fewer per 1.000 (from 272 fewer to 460 more)	⊕⊕◯◯ Low	IMPORTANT
									No effect on FEV1 decline: p=0.962 and p=0.828 for absolute and % predictive values. No significant difference in FEV1 evolution in montelukast (9±14 mL/month) vs. placebo (-24±14 mL/month) (p=0.198).<br>Subgroup analysis for BOS stage 1 (n=11/8): FEV1 decline reduced in absolute (p=0.008) and % predictive (p=0.0180) values in montelukast vs. placebo.			
**Malignancies**												
-	-	-	-	-	-	-	-		-		-	IMPORTANT
**Kidney function**												
-	-	-	-	-	-	-	-		-		-	IMPORTANT

* The risk in the intervention group (and its 95% CI) is based on the assumed risk in the comparison group and the relative effect of the intervention (and its 95% CI).
** No retransplants were performed so graft survival corresponded with overall survival.
*** The authors reported most likely on serious side effects instead of serious adverse events.

CI: confidence interval, **RR:** risk ratio.

GRADE Working Group grades of evidence
High certainty: We are very confident that the true effect lies close to that of the estimate of the effect.
Moderate certainty: We are moderately confident in the effect estimate: The true effect is likely to be close to the estimate of the effect, but there is a possibility that it is substantially different.
Low certainty: Our confidence in the effect estimate is limited: The true effect may be substantially different from the estimate of the effect.
Very low certainty: We have very little confidence in the effect estimate: The true effect is likely to be substantially different from the estimate of effect.

Explanations
1) Single-center randomized controlled trial in patients with late-onset (> 2 years post-transplant) BOS. Rapid decliners > 150 mL/month during 3 months before inclusion were excluded.
a. Downgraded two levels for imprecision due to few studies, participants and events and due to uncertainty regarding the estimated effect (wide variation).

Graft survival at 1 year

Study or Subgroup	Montelukast Events	Montelukast Total	Placebo Events	Placebo Total	Weight	Risk Ratio M-H, Fixed, 95% CI
Ruttens et al., 2018	1	15	1	15	100.0%	1.00 [0.07, 14.55]
**Total (95% CI)**		**15**		**15**	**100.0%**	**1.00 [0.07, 14.55]**
Total events	1		1			
Heterogeneity: Not applicable						
Test for overall effect: Z = 0.00 (P = 1.00)						

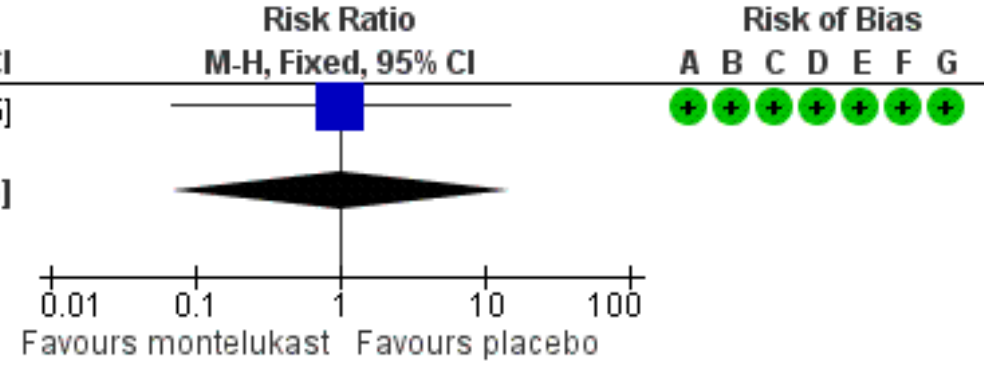

Graft survival at 2 years

Study or Subgroup	Montelukast Events	Montelukast Total	Placebo Events	Placebo Total	Weight	Risk Ratio M-H, Fixed, 95% CI
Ruttens et al., 2018	2	15	5	15	100.0%	0.40 [0.09, 1.75]
**Total (95% CI)**		**15**		**15**	**100.0%**	**0.40 [0.09, 1.75]**
Total events	2		5			
Heterogeneity: Not applicable						
Test for overall effect: Z = 1.22 (P = 0.22)						

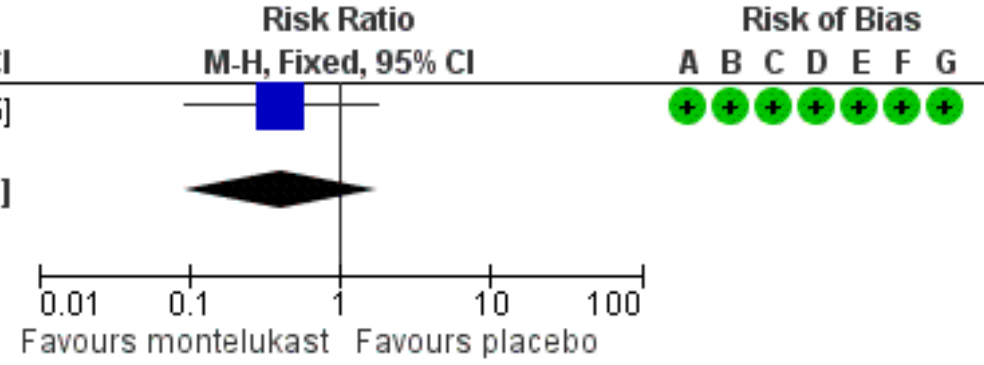

FEV1 decline at 6 months

Study or Subgroup	Montelukast Events	Montelukast Total	Placebo Events	Placebo Total	Weight	Risk Ratio M-H, Fixed, 95% CI
Ruttens et al., 2018	5	15	6	15	100.0%	0.83 [0.32, 2.15]
**Total (95% CI)**		**15**		**15**	**100.0%**	**0.83 [0.32, 2.15]**
Total events	5		6			
Heterogeneity: Not applicable						
Test for overall effect: Z = 0.38 (P = 0.71)						

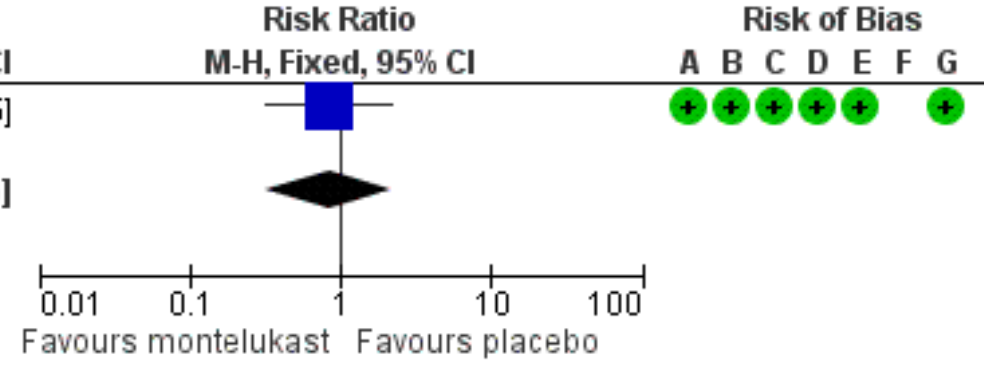

Risk of bias legend
(**A**) Random sequence generation (selection bias)
(**B**) Allocation concealment (selection bias)
(**C**) Blinding of participants and personnel (performance bias)
(**D**) Blinding of outcome assessment (detection bias)
(**E**) Incomplete outcome data (attrition bias)
(**F**) Selective reporting (reporting bias)
(**G**) Other bias

Additional studies

Study	Study Design	Number of patients	Study Summary	Summary of Relevant Outcomes
**Verleden et al., 2011[1]**	Open label, single-center cohort study	n=22: 11 intervention, 11 controls	- BOS stage 1 or 2 with BAL neutrophilia < 15%<br>- Montelukast 10 mg OD, initiated < 6 weeks after BOS diagnosis<br>- Follow-up time of at least 6 months<br>- Azithromycin initiated at BOS diagnosis if not already on it<br>- Most patients also received oral steroid taper<br>- 2/11 patients in treatment group were switched from cyclosporine to tacrolimus, 1/11 patients in control group also received TLI<br>- Controls were selected retrospectively	- FEV1 decline reduced from 112±25 mL/month before BOS diagnosis to 13±13 mL/month 6 months after initiation of montelukast (p=0.001).<br>- In the control group, FEV1 decline remained unchanged from 103±20 mL/month before BOS diagnosis to 114±27 mL/month (p=0.55).<br>- FEV1 decline in treatment group differed from non-treated group during 6 months of treatment (p=0.0025).
**Vos et al., 2019[2]**	Retrospective, single-center cohort study	n=153	- CLAD: 75% BOS, 25% RAS<br>- At least 3 months of treatment, with montelukast initiated at least > 3 months after starting azithromycin<br>- Patients were excluded if montelukast was started prior to or within 3 months after initiation of azithromycin	- Median graft loss was 7 years after CLAD diagnosis.<br>- Median OS was 13 years post-transplant and 7 years after CLAD diagnosis.<br>- 81% (122/151) of evaluable patients responded with improvement > 10% in FEV1 or stabilization (FEV1 ±10%), 66% (93/141) at 6 months and 60% at 12 months.<br>- Reduction in FEV1 rate of decline at 3 months (from 47 to 3.0 mL/month, p<0.0001) and 6 months (from 43 to 7 mL/month, p<0.0001).<br>- 39% (59/153) CLAD progression during 12 months after treatment initiation.

[1] Verleden GM, Verleden SE, Vos R, et al. Montelukast for bronchiolitis obliterans syndrome after lung transplantation: a pilot study. Transpl Int. 2011;24(7):651-6.

[2] Vos R, Eynde RV, Ruttens D, et al. Montelukast in chronic lung allograft dysfunction after lung transplantation. J Heart Lung Transplant. 2019;38(5):516-7.
