# Supplementary material for: European society for organ transplantation clinical practice guideline on prevention and treatment of chronic lung allograft dysfunction
Source: Transpl Int. 2026 Sep 18;39:16959. doi: 10.3389/ti.2026.16959 (PMC13630708; doi:10.3389/ti.2026.16959)
Supplement: Supplementary file 3 [file DataSheet3.pdf]

European Society for Organ Transplantation Clinical Practice Guideline on Prevention and Treatment of Chronic Lung Allograft Dysfunction: Online Supplement

Supplement 3: Evidence-to-Decision frameworks

Table of contents

Evidence to Decision (EtD) frameworks for PICO questions 2
PICO question 1 2
PICO question 2 8
PICO question 3 15
PICO question 4 24
PICO question 5 31
PICO question 6 37
PICO question 7 43
PICO question 8 49

Evidence to Decision (EtD) frameworks for PICO questions

PICO question 1

In adult lung transplant recipients, should azithromycin be used to prevent the onset of CLAD?

PROBLEM<br>Is the problem a priority?		
Judgement	Research evidence	Additional considerations
o No<br>o Probably no<br>o Probably yes<br>**X Yes**<br>o Varies<br>o Don't know	Chronic lung allograft dysfunction (CLAD) is thought to be a correlate of chronic alloimmune rejection after lung transplantation, severely limiting survival. There are no effective treatments except for re-transplantation in strictly selected cases. CLAD affects about one third of lung transplant recipients within five years and presents with different phenotypes, including bronchiolitis obliterans syndrome (BOS), with an obstructive spirometric pattern, or restrictive allograft syndrome (RAS), which is associated with a restrictive pulmonary function test and radiological opacities. Mixed or undefined cases of CLAD also exist. CLAD leads to progressive, irreversible lung function decline, culminating in graft failure and increased mortality. Patients affected by the RAS phenotype have a worse prognosis. CLAD remains an important cause of death beyond the first year after transplant. The goal of immunosuppression is to prevent functional decline, graft loss, and mortality and to achieve a good quality of life.	Observational studies have reported a lower risk of CLAD and improved survival in patients receiving prophylactic azithromycin.[1,2]<br>This PICO question will analyze whether oral azithromycin taken before the onset of CLAD may reduce the risk of developing CLAD.

DESIRABLE EFFECTS<br>How substantial are the desirable anticipated effects?		
Judgement	Research evidence	Additional considerations
o Trivial<br>o Small<br>**X Moderate**<br>o Large<br>o Varies<br>o Don't know	Two randomized controlled trials[3,4] have investigated azithromycin for CLAD prevention. Both studies are single-center studies from the same center. In the first study, azithromycin was initiated at discharge and given for two years. In the second study, azithromycin was given preoperatively, then continued until 31 days after transplant. In both studies, the patients were followed for two years. In a follow-up study (retrospective, post-hoc analysis), the patients from the first study[4] were re-examined to assess long-term results.[5]<br><br>Vos et al. reported a lower CLAD incidence at 2 years in the azithromycin group compared to placebo (13% vs. 44%, p=0.017) (RR 0.28, 95%CI 0.12-0.69, p=0.0052).[4] In the long-term follow-up, the lower occurrence of CLAD in the azithromycin group was confirmed, being 28% vs. 51% (p=0.048) at median follow-up of 5.5 years (RR 0.54, 0.30-0.96, p=0.0365) with	There are two additional retrospective single-center studies that provide further support for the effects of azithromycin prophylaxis.[1,2]<br><br>Li et al. reported an association between azithromycin use and improved overall survival (HR 0.59, 95%CI 0.42–0.82, p=0.002) and reduced risk of BOS, though the reduction in CLAD incidence was not statistically significant (HR 0.69, p=0.07).[1] Porras et al. demonstrated a significantly lower risk of CLAD when azithromycin was initiated within 3 weeks after transplant (HR 0.24, p=0.001). They assessed four time points and found that the earliest initiation of azithromycin post-transplant was associated with the lowest CLAD incidence, suggesting the importance of early treatment.[2] Although not a pre-defined outcome of interest, the study by Li et al. also found that azithromycin prophylaxis was associated with a reduced adjusted risk of BLAD (OR: 0.53; 95% CI: 0.33-0.85; p=0.0460).[1]

	significantly longer CLAD-free survival (p=0.024).[5] In the study of early intervention, where azithromycin was given until D31 post-transplant, Van Herck et al. found no effect on CLAD onset at 2 years (18% vs. 15%, p=0.74).[3]<br><br>Overall survival and graft loss outcomes, however, showed no significant differences; but since studies were not sufficiently powered for these outcomes and cross-over was allowed after development of CLAD, there is a risk of bias and imprecision. The notably high incidence of CLAD in the comparator group in the study by Vos et al. (44% at 2 years) may also indicate a risk of bias.<br><br>None of the studies analyzed quality of life.	These findings, although observational, reinforce the potential benefit of early prophylactic azithromycin in reducing CLAD incidence following lung transplantation. Although survival was reported to be better in the group who received azithromycin[1], these analyses are highly uncertain as they may have been subject to statistical immortal time bias.<br><br>In summary, there is evidence to suggest that azithromycin does reduce the fall of FEV1 after lung transplantation, thus delaying or possibly preventing the onset of CLAD. There is, however, no reliable evidence to suggest that prophylactic azithromycin reduces graft loss and improves survival after lung transplantation at the moment. There is also no study to guide the time for initiation of prophylactic azithromycin, although the study by Porres et al.[2] suggests benefit from early post-transplant commencement, and no subgroups that might have increased benefit have been identified.
**UNDESIRABLE EFFECTS**<br>How substantial are the undesirable anticipated effects?		
Judgement	Research evidence	Additional considerations
○ Large<br>○ Moderate<br>**X Small**<br>○ Trivial<br>○ Varies<br>○ Don't know	In the randomized controlled trials by Vos et al.[4] and Van Herck et al.[3], serious adverse events, impairment in kidney function, and infectious complications were not systematically assessed.	In the study by Vos et al., adverse events (defined as serious allergic reactions, angioneurotic oedema and anaphylaxis, cardiac arrhythmias, neurological disorders, gastrointestinal disorders) were not different between groups.[4] Van Herck et al. reported no clinically significant adverse effects, including cardiac toxicity.[3] The retrospective long-term study also reported no difference in QT-time on the ECG.[5] Ototoxicity was not recorded in any of the trials.<br><br>The two other retrospective single-center studies provide additional data on the safety of azithromycin prophylaxis in lung transplant recipients. Neither study identified an increased risk of adverse events, renal dysfunction, or malignancy attributable to azithromycin.[1,2] In Li et al., the distribution of causes of death, including cardiovascular events, did not differ between prophylaxis and non-prophylaxis groups.[1] Porras et al. similarly did not report safety concerns in any treatment group.[2]<br><br>From clinical experience, we know that gastrointestinal intolerance may be an issue, requiring dose reduction or treatment cessation. In clinical practice, combinations with other drugs (e.g., potential of QTc elongation) and ototoxicity should also be taken into consideration. A meta-analysis of randomized controlled trials and observational studies in patients with chronic lung diseases has shown that long-term azithromycin therapy was associated with hearing impairment

		(RR 1.17, 95%CI 1.03-1.33), but audiometry has not been assessed in lung transplant recipients.[6]<br><br>Of note, in another study from Leuven, azithromycin prophylaxis did not seem to affect bacterial microbiota in samples from the bronchi of lung transplanted patients.[7]<br><br>In general, azithromycin is relatively well tolerated. However, it is important to be mindful that several forms of adverse effects, such as ototoxicity, have not been systematically studied. Also, long-term effects of permanent use of azithromycin are not well known.

CERTAINTY OF EVIDENCE

What is the overall certainty of the evidence of effects?

Judgement	Research evidence	Additional considerations
**X Very low**<br>○ Low<br>○ Moderate<br>○ High<br>○ No included studies	The overall certainty of the evidence for prophylactic azithromycin in lung transplant recipients is rated as very low, based on the lowest certainty among critical outcomes. For graft loss and all-cause mortality, the certainty was downgraded by one level due to risk of bias, principally related to cross-over in treatment allocation following CLAD onset, and by two levels for imprecision, owing to small sample sizes and a limited number of events.<br><br>Evidence for CLAD prevention was rated moderate and downgraded one level for imprecision, reflecting reliance on a single RCT with limited events.	Lung transplantation is a low-incidence, highly specialized intervention, which limits the feasibility of large, high-quality randomized trials. The small number of eligible patients, combined with the need for long-term follow-up to capture outcomes such as CLAD, graft loss, and late mortality, increases the risk of attrition and resource demands. Heterogeneity across centers and across time periods in surgical techniques, immunosuppressive regimens, and clinical practices further complicates trial design. As a result, current evidence is largely derived from small, single-center studies with low event rates.

VALUES

Is there important uncertainty about or variability in how much people value the main outcomes?

Judgement	Research evidence	Additional considerations
○ Important uncertainty or variability<br>○ Possibly important uncertainty or variability<br>○ Probably no important uncertainty or variability<br>**X No important uncertainty or variability**	No included studies.	Qualitative studies and patient-reported outcomes consistently show that avoiding chronic rejection and preserving long-term graft function are highly valued among lung transplant recipients.[8,9] This is confirmed by the "Lung Transplant Foundation" initiative on BOS (https://lungtransplant-foundation.org/resources-topics/bos/).<br><br>It is therefore reasonable to assume that most patients would be very willing to prevent CLAD development and that they would be willing to accept mild side effects and additional follow-up if there is even a small chance of reducing the risk of CLAD.

BALANCE OF EFFECTS

Does the balance between desirable and undesirable effects favor the intervention or the comparison?

Judgement	Research evidence	Additional considerations

○ Favors the comparison<br>○ Probably favors the comparison<br>○ Does not favor either the intervention or the comparison<br>**X Probably favors the intervention**<br>○ Favors the intervention<br>○ Varies<br>○ Don't know	Based on the current evidence, the balance of effects appears to favor azithromycin for the prevention of CLAD in adult lung transplant recipients. Data demonstrated a clinically meaningful reduction in the incidence of CLAD. Adverse effects associated with azithromycin were reported to be mild and infrequent, with no significant increase in serious complications such as infections, nephrotoxicity, or malignancy. Given the high clinical impact of CLAD and the good safety profile of azithromycin, the overall balance of effects was judged to be in favor of its use in this population.	In clinical experience, azithromycin is often incorporated into standard post-transplant protocols for lung recipients at 72% of lung transplant centers who participated in a recent study[10], reflecting its established anti-inflammatory properties and favorable safety profile. Observational studies, such as those by Li et al.[1] and Porras et al.[2], have shown that early use of azithromycin may lower the risk of CLAD and improve survival. Many transplant centers now include azithromycin in their standard protocols. While high-certainty evidence from randomized controlled trials remains limited, the consistency of beneficial effects across study designs and settings, together with the low incidence of adverse events, supports its widespread use. These considerations reinforce the panel's judgment in favor of routine prophylactic use. On the other hand, it needs to be mentioned that potential undesirable effects (such as ototoxicity) and long-term effects are not regularly assessed.<br><br>Further studies addressing this would be useful, particularly considering that many centers already give universal prophylaxis using azithromycin. Also, studies to determine optimal time-point to start this prophylaxis, and studies defining possible subgroups with particular benefit or risk would be helpful.
**RESOURCES REQUIRED**<br>How large are the resource requirements (costs)?		
Judgement	Research evidence	Additional considerations
○ Large costs<br>○ Moderate costs<br>**X Negligible costs - unknown savings**<br>○ Moderate savings<br>○ Large savings<br>○ Varies<br>○ Don't know	No studies included.	The resource requirements for implementing azithromycin prophylaxis in lung transplant recipients are considered minimal. Azithromycin is available as a low-cost generic medication, and the cost per dose is substantially lower than that of standard immunosuppressive agents. When incorporated into existing post-transplant protocols, it does not necessitate additional personnel, infrastructure, or specialized monitoring beyond routine follow-up. While medication costs may vary across healthcare systems, the overall financial impact is expected to be modest. In healthcare settings where azithromycin is already included in institutional formularies or reimbursed by insurance, minimal additional resources would be needed for implementation. Also, the resources needed to monitor side effects are mostly within what would be reasonable to expect at a transplant center.<br><br>Avoiding CLAD would clearly be beneficial also in a resource perspective.[11]
**CERTAINTY OF EVIDENCE OF REQUIRED RESOURCES**<br>What is the certainty of the evidence of resource requirements (costs)?		
Judgement	Research evidence	Additional considerations

o Very low<br>o Low<br>o Moderate<br>o High<br>**X No included studies**	No studies included.	

COST-EFFECTIVENESS<br>Does the cost-effectiveness of the intervention favor the intervention or the comparison?		
Judgement	Research evidence	Additional considerations
o Favors the comparison<br>o Probably favors the comparison<br>o Does not favor either the intervention or the comparison<br>**X Probably favors the intervention**<br>o Favors the intervention<br>o Varies<br>o No included studies	No studies included.	No cost-effectiveness studies evaluating azithromycin specifically for the prevention of CLAD in lung transplant recipients are currently available. Existing economic evaluations in other populations, such as those with COPD or cystic fibrosis, are not generalizable to the lung transplant setting due to differing disease mechanisms, treatment goals, and outcome priorities.<br><br>Although the cost of using azithromycin is relatively low compared to other costs in lung transplantation, a cost-effectiveness analysis would have to take into account that the proven effect on survival is currently uncertain.

EQUITY<br>What would be the impact on health equity?		
Judgement	Research evidence	Additional considerations
o Reduced<br>o Probably reduced<br>**X Probably no impact**<br>o Probably increased<br>o Increased<br>o Varies<br>o Don't know	No studies included.	No impact on health equity is likely as azithromycin is relatively widely available, certainty in countries performing lung transplantation.

ACCEPTABILITY<br>Is the intervention acceptable to key stakeholders?		
Judgement	Research evidence	Additional considerations
o No<br>o Probably no<br>o Probably yes<br>**X Yes**<br>o Varies<br>o Don't know	No studies included.	Azithromycin is widely used in other chronic respiratory diseases, such as COPD and cystic fibrosis and bronchiectasis, and is generally regarded as a safe and well-tolerated intervention. In the context of lung transplantation, both patients and healthcare professionals are familiar with its use, and concerns related to safety or adherence are minimal. Given the high clinical relevance of preventing CLAD and the absence of effective alternative prophylactic strategies, the intervention is considered acceptable. Its oral formulation, low cost, and favorable safety profile contribute to its feasibility and acceptability among key stakeholders involved in the management of lung transplant recipients.[10,12]

FEASIBILITY		

Is the intervention feasible to implement?		
Judgement	Research evidence	Additional considerations
○ No<br>○ Probably no<br>○ Probably yes<br>**X Yes**<br>○ Varies<br>○ Don't know	No studies included.	Initiation of azithromycin in adult lung transplant recipients is considered highly feasible. The drug is orally administered and can be initiated early post-transplant without requiring hospitalization or additional infrastructure. Standard dosing (e.g., 250 mg three times weekly or every other day) can be started during routine follow-up visits and integrated into existing immunosuppressive protocols. No specialized monitoring is required beyond standard post-transplant care. Given its widespread availability, low cost, and clinician familiarity, initiation of azithromycin does not pose logistical challenges and is readily implementable across transplant centers.

TYPE OF RECOMMENDATION				
Strong recommendation against the intervention	Conditional recommendation against the intervention	No recommendation against the intervention or in favor of the intervention	Conditional recommendation for the intervention	Strong recommendation for the intervention
☐	☐	☐	☒	☐

RECOMMENDATION

- In adult lung transplant recipients, we suggest using azithromycin in the prevention of CLAD. (Conditional recommendation in favor of the intervention, very low certainty of evidence)

REMARKS

JUSTIFICATION

The Task Force group was in favor of using azithromycin in the prevention of CLAD, as studies have shown a clinically meaningful reduction in the incidence of CLAD and azithromycin was generally well tolerated, with mild and infrequent adverse events.

SUBGROUP CONSIDERATIONS

IMPLEMENTATION CONSIDERATIONS

Azithromycin is orally administered and can easily be initiated during index admission or routine follow-up visits.
No specialized monitoring is required beyond standard post-transplant care.

FURTHER RESEARCH SUGGESTIONS

If further research on azithromycin were to be performed, it could focus on the optimal time-point to start azithromycin after lung transplantation and whether this should be universal or targeted to certain risk groups.
Since patient-reported outcome measures are lacking, it would also be important to include these.
Long-term effects of azithromycin should also be studied further, especially the impact on graft and overall survival.

PICO question 2

In adult lung transplant recipients, should tacrolimus be used over cyclosporine to prevent the onset of CLAD?

PROBLEM
Is the problem a priority?

Judgement	Research evidence	Additional considerations
o No<br>o Probably no<br>o Probably yes<br>**X Yes**<br>o Varies<br>o Don't know	Chronic lung allograft dysfunction (CLAD) is thought to be a correlate of chronic alloimmune rejection after lung transplantation, severely limiting survival. There are no effective treatments except for re-transplantation in strictly selected cases. CLAD affects about one third of lung transplant recipients within five years and presents with different phenotypes, including bronchiolitis obliterans syndrome (BOS), with an obstructive spirometric pattern, or restrictive allograft syndrome (RAS), which is associated with a restrictive pulmonary function test and radiological opacities. Mixed or undefined cases of CLAD also exist. CLAD leads to progressive, irreversible lung function decline, culminating in graft failure and increased mortality. Patients affected by the RAS phenotype have a worse prognosis. CLAD remains an important cause of death beyond the first year after transplant. The goal of immunosuppression is to prevent functional decline, graft loss, and mortality and to achieve a good quality of life.	Calcineurin inhibitors (CNIs) are a central part of the immunosuppressive regimen for all lung transplant patients and are in fact the drug class that made such transplants possible. Their immunosuppressive effect is thought to mainly be mediated through inhibition of T-cell function, but unfortunately, a wide array of undesired side effects is common. Currently, there are two used CNIs, cyclosporine and tacrolimus, and their effects are not identical. Determining which of the two is the preferable drug to prevent the onset of CLAD is of great importance.

DESIRABLE EFFECTS
How substantial are the desirable anticipated effects?

Judgement	Research evidence	Additional considerations
o Trivial<br>o Small<br>o Moderate<br>**X Large**<br>o Varies<br>o Don't know	The prespecified desirable effects are prevention of CLAD, graft loss and overall survival. No studies reported on quality of life outcomes.<br><br>Four randomized controlled trials published between 2003 and 2024 were included, comprising a total of 662 patients, 329 allocated to tacrolimus and 333 to cyclosporine with a follow-up between 507 days and 3 years.[13-16] The largest and most comprehensive trial was conducted by Dellgren et al., enrolling 249 patients.[13]<br><br>Graft loss was reported in three studies, with 71 events among 573 patients; the pooled risk ratio (RR) was 0.70 (95%CI: 0.46–1.06), with no statistically significant difference between groups (p=0.10).[13,14,16] However, these studies were not powered for this outcome to detect a clinically meaningful effect.<br><br>Overall survival was assessed in all four studies, with 144 events in 662 patients; the pooled RR was 0.91 (95%CI: 0.68–1.21, p=0.52), demonstrating no significant difference between groups.[13-16]<br><br>CLAD incidence was reported in four studies, with 134 events in 662 patients; the pooled RR was 0.40 (95%CI: 0.28–0.57, p<0.00001), showing a significantly lower incidence in the tacrolimus group.[13-16]	Three quasi-randomized studies were identified[17-19] in addition to three observational studies[20-22].<br><br>Regarding overall survival, one study showed no difference[22], whereas two studies showed higher overall survival in the tacrolimus group[20,21]. In the quasi-randomized trials by Griffith et al.[17] and Treede et al.[19], one-year survival did not differ significantly, whereas in the one by Keenan et al., survival was higher in the tacrolimus group.[18]<br><br>In the quasi-randomized study by Keenen et al., CLAD incidence was significantly lower in the tacrolimus group (22%) compared with the cyclosporine group (38%, p=0.025).[18] In the quasi-randomized trial by Griffith et al., CLAD occurred in two patients (5%) under tacrolimus and four patients (11%) under cyclosporine.[17]<br><br>In considering all these studies and results it should be noted that different through levels were used in various protocols, and that there is no consensus on best through level targets at different timepoints after transplantation.<br><br>Using the International Society of Heart and Lung Transplantation (ISHLT) registry, the use of CNI and the incidence of CLAD and graft survival was assessed. Data were available for more than 22.000 lung transplant recipients, 88.6% receiving tacrolimus. It was demonstrated that patients receiving cyclosporine vs. tacrolimus for maintenance CNI had an increased risk of

<table>
<tr><td></td><td>In summary, the CLAD endpoint is the main positive signal, and is in favour of tacrolimus. Mortality and re-transplantation appear neutral in this (short) time window, and were not sufficiently powered.</td><td>CLAD and decreased overall allograft survival in real world clinical practice.[23]

Other solid organ transplantation:
Across solid organs, tacrolimus generally outperforms cyclosporine on early efficacy, but convincing prevention of any chronic allograft dysfunction (CAD) is organ-specific and often indirect.

Kidney: In kidney transplantation, a large Cochrane/BMJ meta-analysis of 30 RCTs (n≈4,100) found tacrolimus reduced acute rejection and improved death-censored graft survival up to 3 years (RR ~0.71), at the cost of more insulin-requiring diabetes; trials seldom adjudicated “chronic allograft nephropathy” rigorously, so CAD prevention is inferred from better graft survival rather than direct histology/IFTA endpoints.[24]
Liver: In liver transplantation, a Cochrane review of 16 RCTs (n=3813) showed tacrolimus improved 1-year patient and graft survival and reduced acute and steroid-resistant rejection versus cyclosporine; chronic rejection events were uncommon and not consistently reported, so any CAD benefit is mediated through fewer early immune injuries and lower discontinuation.[25]
Heart: In heart transplantation, a systematic review and meta-analysis of randomized trials[26] (10 RCTs, n=952) reported less severe rejection and better lipid/hypertension profiles with tacrolimus, and a mortality advantage over microemulsion cyclosporine, but no clear between-drug difference in renal dysfunction, diabetes, infection or malignancy; critically, randomized evidence has not shown a consistent reduction in cardiac allograft vasculopathy, and small IVUS studies are mixed, while contemporary overviews emphasize mTOR-based regimens, not CNI choice as the strategy most associated with slower vasculopathy progression.

In summary, tacrolimus improves early rejection control and short-term graft survival over cyclosporine in kidney and liver transplants and is at least non-inferior in heart transplants, but robust, direct evidence that tacrolimus prevents late CAD beyond these surrogate gains is limited in SOT outside lung transplantation.</td></tr>
<tr><td colspan="3">UNDESIRABLE EFFECTS
How substantial are the undesirable anticipated effects?</td></tr>
<tr><td>Judgement</td><td>Research evidence</td><td>Additional considerations</td></tr>
<tr><td>○ Large
○ Moderate
X Small
○ Trivial
○ Varies
○ Don't know</td><td>The undesired effects profile is broadly comparable between tacrolimus and cyclosporine over 1–3 years. The relevant prespecified GRADE endpoints were serious adverse events, infections, kidney dysfunction, and malignancy.

Serious adverse events, one of our critical outcomes, were only reported in the ScanCLAD trial, with no difference between groups (449 vs 448 events, p=0.40).[13]

In pooled data from three studies, the infection rate per 100 patient-days was 87/205 in the tacrolimus group versus 94/208 in the cyclosporine group (RR</td><td>In the quasi-randomized and observational studies, Keenan et al.[18] and Reichenspurner et al.[21] reported no difference in the incidence of infections between both groups. Regarding kidney dysfunction, the latter study found no significant difference between tacrolimus and cyclosporine.

Other solid organ transplantation:
Overall, tacrolimus was associated with a higher risk of post-transplant diabetes mellitus and neurotoxicity, and lower incidence of hypertension compared with cyclosporine.[25,27] In kidney transplantation, tacrolimus increased the risk of diabetes and certain neurological and gastrointestinal side effects.[24] Regarding renal outcomes</td></tr>
</table>

	0.94; 95%CI: 0.76–1.16, p=0.57).[14-16] The study by Dellgren et al. also found no difference in treated infections, with 90% affected in both groups (111/124 in tacrolimus vs. 113/125 in cyclosporine).[13]<br><br>Regarding kidney dysfunction, defined as a serum creatinine > 2 mg/mL, in a pooled analysis of four studies, 132 events occurred with tacrolimus and 103 with cyclosporine (RR 1.29, 1.08-1.55, p=0.006).[13-16] Overall in the ScanCLAD trial, the risk of kidney dysfunction was modestly higher in tacrolimus. Following a protocol amendment, XR-tacrolimus was no longer used to determine initial dose, which was particularly relevant in the ICU patients and early toxic doses occurred. After this, kidney failure was no longer more frequent in patients treated with tacrolimus. Throughout the study, attained through levels were too high in 20% in both groups at all timepoints, too low was somewhat more frequent in the cyclosporine group, which may also have affected the renal endpoint.[13]<br><br>Finally, malignancy was reported in three studies with 21 vs. 19 events in the tacrolimus vs. cyclosporine groups (RR 1.10, 95%CI 0.62-1.97, p=0.74).[13-15]	after kidney transplantation, tacrolimus provided better outcomes.[28,29]<br><br>The differential clinical side effects/adverse events between tacrolimus and cyclosporine may be different in clinical perspective, but this is not extensively studied given large amount of side effects experienced.<br><br>Conversion to once daily (extended release) vs. twice daily dosing (immediate release) tacrolimus was safe with respect to renal function, metabolic parameters and allograft function, and improved patient adherence.[30] Whether there is a benefit of once daily (extended release) dosing over twice daily dosing regarding side effects (nefrotoxicity, neurotoxicity, etc.) is not well known.<br><br>The Revolution Trial (NCT05001074) will compare the outcomes of extended-release tacrolimus to immediate-release tacrolimus in lung transplant recipients, with the hypothesis that the extended formulation has less adverse effects. The primary endpoint is the absolute difference in eGFR slope at the end of the study. Secondary outcomes include incidence of infection, acute cellular rejection, post-transplant diabetes mellitus and new-onset hypertension. Potential harms, other than those already mentioned, are any adverse events needing treatment. Study results are expected in 2026, and may provide further guidance on the tacrolimus formulation of choice.[31]

CERTAINTY OF EVIDENCE<br>What is the overall certainty of the evidence of effects?		
Judgement	Research evidence	Additional considerations
o Very low<br>**X Low**<br>o Moderate<br>o High<br>o No included studies	The overall certainty of evidence for our critical outcomes (graft survival, overall survival and CLAD incidence) is low. In more detail, the certainty of evidence in favour of tacrolimus to prevent CLAD is moderate, due to downgrading only for risk of bias. In contrast, certainty is lower for graft loss and overall survival, with a low certainty of evidence, due to downgrading for risk of bias and imprecision.	To create high-level evidence, it is important to choose outcomes upfront, and to power studies accordingly (e.g., to assess the effect on overall survival and graft survival). At the moment, there was unfortunately a lack of data for these outcomes, given these studies were short-term studies and underpowered. The panel considers it important that the effect on CLAD incidence was of moderate certainty and will most likely affect graft and overall survival in the end, although this has to be monitored in future studies.

VALUES<br>Is there important uncertainty about or variability in how much people value the main outcomes?		
Judgement	Research evidence	Additional considerations
o Important uncertainty or variability<br>o Possibly important uncertainty or variability<br>**X Probably no important uncertainty or variability**<br>o No important	No included studies.	Patient values and preferences likely vary. Many patients will prioritize avoiding CLAD and preserving lung function above all else, yet some will place a higher weight on avoiding nephrotoxicity or diabetes. Because both CLAD and several side effects often emerge beyond three to five years, shared decision-making should explicitly surface these trade-offs and plan for ongoing re-evaluation as longer-term ScanCLAD follow-up becomes available. The evidence dossier notes no direct studies of patient preferences comparing the value of CLAD avoidance to the burden of long-term adverse effects.

uncertainty or variability		Both CLAD and several of the side effects of immunosuppression may manifest long after transplantation, often meaning more than 3-5 years, such as chronic kidney failure and malignancies. For many patients, taking immunosuppressive drugs for life is a burden, and including patient-reported outcomes and patient's input on adverse events and side effects related to immunosuppression should be encouraged in all future trials. Some studies report on patient values, such as in Keenan et al., 1995.[18] Recipient acceptance of the immunosuppressive agent was notably greater in patients receiving tacrolimus because of the absence of gingival hyperplasia and hirsutism.<br><br>In heart transplantation, a prospective randomized study found that patients treated with tacrolimus reported statistically significant improvements in health-related quality of life, particularly in the psychological domains of vitality and mental health, compared to those treated with cyclosporine. This cautiously suggests there may be a patient preference for tacrolimus in terms of psychological well-being and overall health-related quality of life after transplantation.[32]
**BALANCE OF EFFECTS**<br>Does the balance between desirable and undesirable effects favour the intervention or the comparison?		
Judgement	Research evidence	Additional considerations
○ Favors the comparison<br>○ Probably favors the comparison<br>○ Does not favor either the intervention or the comparison<br>○ Probably favors the intervention<br>**X Favors the intervention**<br>○ Varies<br>○ Don't know	The desirable effects of tacrolimus on CLAD prevention appear to outweigh the undesirable effects in the first 1–3 years post-transplant, particularly given the absence of survival or serious adverse event penalties and only a modest trade-off in renal events.	The majority (~90%) of lung transplant centers worldwide[33] and in the US[34] use tacrolimus as the primary immunosuppressant.<br><br>This favorable balance of effects with tacrolimus mirrors findings in other solid organ transplant.
**RESOURCES REQUIRED**<br>How large are the resource requirements (costs)?		
Judgement	Research evidence	Additional considerations
○ Large costs<br>○ Moderate costs<br>○ Negligible costs and savings<br>**X Moderate savings**<br>○ Large savings<br>○Varies<br>○ Don't know	No studies included.	The cost of tacrolimus is similar to the cost of cyclosporine, in terms of drug pricing as well as the associated expenses for administration and therapeutic monitoring. Tacrolimus is associated with fewer CLAD events, and downstream resource use is therefore likely to be lower.
**CERTAINTY OF EVIDENCE OF REQUIRED RESOURCES**<br>What is the certainty of the evidence of resource requirements (costs)?		
Judgement	Research evidence	Additional considerations

o Very low<br>o Low Moderate<br>o High<br>**X No included studies**	No studies included.	

COST-EFFECTIVENESS
Does the cost-effectiveness of the intervention favour the intervention or the comparison?

Judgement	Research evidence	Additional considerations
o Favors the comparison<br>o Probably favors the comparison<br>o Does not favor either the intervention or the comparison<br>X Probably favors the intervention<br>o Favors the intervention<br>o Varies<br>**X No included studies**	No studies included.	Both drugs are widely available even as generics in transplant programs, with similar acquisition and monitoring costs. Because the main effect of tacrolimus is fewer CLAD events, downstream resource use is likely to be lower, but formal cost-effectiveness studies are lacking.<br><br>Tacrolimus appears cost-effective due to its comparable drug and monitoring costs to cyclosporine, combined with its significantly lower incidence of CLAD, which may reduce long-term healthcare costs.<br><br>In support of this: There are studies demonstrating that tacrolimus is more cost-effective than cyclosporine for immunosuppression in kidney transplantation, primarily due to improved graft survival, lower acute rejection rates, and a more favorable side effect profile, despite a higher risk of post-transplant diabetes.[29]<br><br>Potentially, additional cost-effectiveness data regarding the ScanCLAD study can be expected.

EQUITY
What would be the impact on health equity?

Judgement	Research evidence	Additional considerations
o Reduced<br>o Probably reduced<br>**X Probably no impact**<br>o Probably increased<br>o Increased<br>o Varies<br>o Don't know	No studies included.	Tacrolimus and cyclosporine are widely available in lung transplant centers and have comparable pricing. Both are standard of care worldwide with established monitoring infrastructures. Therefore, equity, acceptability, and feasibility are unlikely to differ.

ACCEPTABILITY
Is the intervention acceptable to key stakeholders?

Judgement	Research evidence	Additional considerations
o No<br>o Probably no<br>o Probably yes<br>**X Yes**<br>o Varies<br>o Don't know	No studies included.	Acceptability appears to be high for tacrolimus as the preferred immunosuppressant. The side effect profiles of tacrolimus and cyclosporine differ slightly, which may influence individual patient preferences.<br><br>In heart transplantation, a prospective randomized study found that patients treated with tacrolimus reported statistically significant improvements in health-related quality of life, particularly in the psychological domains of vitality and mental health, compared to those treated with cyclosporine. This cautiously suggests there may also be a patient preference for tacrolimus in terms of

		psychological well-being and overall health-related quality of life after transplantation.[32]<br><br>In a recent pan-European survey, representing 74% of the European lung transplant activity, it was found that 95% of centers use tacrolimus as the standard CNI in their protocol.[10]
**FEASIBILITY**<br>Is the intervention feasible to implement?		
Judgement	Research evidence	Additional considerations
o No<br>o Probably no<br>o Probably yes<br>**X Yes**<br>o Varies<br>o Don't know	No studies included.	Both drugs are widely available across all lung transplant centers, often in several administration formulas. They both require therapeutic drug monitoring. We see no feasibility concerns in preferring tacrolimus over cyclosporine.

**TYPE OF RECOMMENDATION**				
Strong recommendation against the intervention	Conditional recommendation against the intervention	No recommendation against the intervention or in favor of the intervention	Conditional recommendation for the intervention	Strong recommendation for the intervention
☐	☐	☐	☐	☒

RECOMMENDATION

- In adult lung transplant recipients, we recommend using tacrolimus over cyclosporine in the prevention of CLAD. (Strong recommendation in favor of the intervention, low certainty of evidence)

REMARKS

JUSTIFICATION

The systematic review yielded good and consistent evidence for the effect of tacrolimus on CLAD incidence, with tacrolimus being preferred over cyclosporine. Given the moderate certainty of evidence for this outcome, the panel issued a strong recommendation for its use, even though data on its effect on long-term graft and overall survival are currently insufficient. The registry study, which included a longer follow-up up to 14 years post-transplant, demonstrated an increased overall risk of death/retransplant in cyclosporin-treated patients. More long-term data regarding graft and overall survival are desirable.

SUBGROUP CONSIDERATIONS

IMPLEMENTATION CONSIDERATIONS

There are no specific implementation concerns, as both tacrolimus and cyclosporine are widely available and are on the WHO essential medicine list.

It is currently unclear if once daily dosing (extended-release formulation) may be beneficial over twice daily dosing (immediate-release formulation) regarding adverse events and side effects. Ongoing and future studies may provide further guidance on this.
Cyclosporine remains an essential drug for patients who experience unacceptable side effects from tacrolimus.

FURTHER RESEARCH SUGGESTIONS

- Long-term data (> 3 years) for overall survival and graft survival. (Updated results of Dellgren et al. (ScanCLAD) will be reported)
- Effects of once daily dosing vs. twice daily, and of different formulations on renal outcomes and other side effects.
- Management of unacceptable side effects (such as thrombotic microangiopathy, posterior reversible encephalopathy syndrome).
- Quality of life of tacrolimus vs. cyclosporine in lung transplantation. (Follow-up reports from the ScanCLAD study are expected)
- Ideal through levels for both CNI at various time points after transplantation, including feasibility and safety of CNI dose reduction.
- CNI target level adjustments related to kidney function, risk factors, immunological risk, drug interactions etc.
- Ideal management of therapeutic drug monitoring (frequency, method, dosage adjustments).
- Cost-effectiveness of tacrolimus vs. cyclosporine in lung transplantation.

PICO question 3

In adult lung transplant recipients, should mycophenolate mofetil be used over azathioprine to prevent the onset of CLAD?

PROBLEM<br>Is the problem a priority?		
Judgement	Research evidence	Additional considerations
○ No<br>○ Probably no<br>X Probably yes<br>**X Yes**<br>○ Varies<br>○ Don't know	Chronic lung allograft dysfunction (CLAD) is thought to be a correlate of chronic alloimmune rejection after lung transplantation, severely limiting survival. There are no effective treatments except for re-transplantation in strictly selected cases. CLAD affects about one third of lung transplant recipients within five years and presents with different phenotypes, including bronchiolitis obliterans syndrome (BOS), with an obstructive spirometric pattern, or restrictive allograft syndrome (RAS), which is associated with a restrictive pulmonary function test and radiological opacities. Mixed or undefined cases of CLAD also exist. CLAD leads to progressive, irreversible lung function decline, culminating in graft failure and increased mortality. Patients affected by the RAS phenotype have a worse prognosis. CLAD remains an important cause of death beyond the first year after transplant. The goal of immunosuppression is to prevent functional decline, graft loss, and mortality and to achieve a good quality of life.	Although data from the ISHLT transplant registry[33] indicate that mycophenolate mofetil (MMF) is the most commonly used antiproliferative agent following lung transplantation, there is currently no conclusive evidence demonstrating its superiority over azathioprine (AZA) in preventing chronic rejection. Determining which of these two drugs is more effective remains a key priority in both clinical practice and transplant research. This is particularly important because current preventive strategies against chronic rejection are significantly more effective than the available therapeutic options once chronic rejection has developed. Optimizing immunosuppressive regimens with the most effective agents could therefore have a major impact on long-term outcomes for lung transplant recipients. Drug conversion (from AZA to MMF and vice versa) may occur due to various reasons post-transplant, obscuring assessment of the treatment effects of either drug regarding CLAD prevention in these transplant recipients.
DESIRABLE EFFECTS<br>How substantial are the desirable anticipated effects?		
Judgement	Research evidence	Additional considerations
**X Trivial**<br>○ Small<br>○ Moderate<br>○ Large<br>○ Varies<br>○ Don't know	Two randomized prospective, open-label, multicenter studies with a total of 399 patients assessed outcomes after lung transplantation in patients treated with AZA vs. MMF.[35,36]<br><br>Mc Neil et al. found that overall patient survival up to 3 years was 75% in the MMF group vs. 69% in the AZA group (p=0.18). Their primary endpoint was BOS incidence at 3 years post-randomization; they found no differences between the two treatment groups in the incidence, severity or time to acquisition of BOS (incidence of BOS, MMF vs. AZA: OR 1.003, 95% CI 0.598, 1.682). BOS-free survival at 3 years was 73% in the MMF group vs. 75% in the AZA group. Alongside, BOS grades were similar in the two groups at 3 years. However, the composite endpoint of BOS, death and re-transplantation due to graft failure or withdrawal because of lack of therapeutic response showed a significant benefit for MMF over AZA (OR 0.582, 95% CI 0.363-0.933), but this significant difference was mainly driven by the withdrawal	Several retrospective studies provided data on the incidence of CLAD with different immuno-suppressive regimens.[14,20,21,37-39]<br><br>Reichenspurner et al. did not report any case of CLAD in MMF patients, but follow-up was limited.[21] Method of follow-up was unclear in the Tague's study, which reported a higher incidence of CLAD in MMF (58%) compared to AZA (49%), albeit not significant.[39] Bhorade's and later Erdmann's groups found no significant differences between AZA and MMF patients in terms of survival and CLAD incidence.[20,37] Speich et al, instead, reported a significantly lower graft loss rate and a trend towards better survival in the MMF-treated patients, despite the fact that the cumulative incidence of CLAD at 5 years was slightly higher in the AZA group (41% vs. 35%, not significant). Of note, in this study, 42% were initially treated with AZA and then converted to MMF for clinical reasons, prohibiting firm conclusions.[38]

	because of lack of therapeutic response component of this composite endpoint.[35]<br><br>The study by Palmer et al. provided no information on CLAD onset, but reported similar 6-month survival rates, 86% in the MMF and 82% in the AZA group, p=0.57.[36]<br><br>Given the limited sample size and follow-up duration, as well as substantial crossover in treatment allocation, the overall level of evidence was very low.	Most recently, a retrospective cohort study in 224 patients (88 on AZA, 136 on MMF) also demonstrated similar rates of CLAD, graft loss, and infection.[40]<br><br>**ACR**<br>Albeit not one of our predefined outcomes, some studies also provided data on the incidence of acute cellular rejection (ACR).<br>Both randomized controlled trials that were included, found no significant difference in the incidence of biopsy-proven acute rejection at 6 months between MMF and azathioprine (58% vs. 63%, p=0.82) and no difference in the incidence or timing of acute rejection at 1 and 3 years.[35,36]<br>However, several non-randomized and retrospective studies have suggested that MMF may be associated with reduced risk of ACR. The study by Sanborn et al. found a greater risk of clinically significant rejection rates in the AZA group compared to the MMF group (HR 1.76, 95% CI 1.12–2.79, p=0.02)[40], and three other studies[14,38,41] found similar results.<br><br>**Pharmacogenetics**<br>Pharmacogenetic studies have shown that polymorphisms in UGT enzymes, which regulate the bioavailability of mycophenolic acid—the active metabolite of MMF—may influence the drug's effectiveness and side effects in solid organ transplantation.[42] Additional testing may be beneficial.<br><br>**EVIDENCE FROM OTHER ORGANS**<br>**OTHER SOT EXPERIENCES/DATA**<br>In the Consensus recommendations for use of maintenance immunosuppression in solid organ transplantation guidelines, endorsed by the American College of Clinical Pharmacy, American Society of Transplantation, and the International Society for Heart and Lung Transplantation, it is concluded that there may be benefit to the use of MPA over AZA for the prevention of acute rejection (2B Kidney).[43] MPA was more effective than AZA in reducing acute rejection rates at 12 months (1C pancreas, 1B liver). MPA has demonstrated better patient and allograft survival over AZA with a decreased incidence and severity of acute rejection (1B heart). Comparative data have variable results although there are some observational and cohort data demonstrating less acute rejection with MPA as compared to AZA and potential benefit in switching to MPA in the setting of BOS (2C lung).[43]
**UNDESIRABLE EFFECTS**<br>How substantial are the undesirable anticipated effects?		
Judgement	Research evidence	Additional considerations

○ Large<br>○ Moderate<br>○ Small<br><br>○ Trivial<br>**X Varies**<br>○ Don't know	Two randomized prospective, open-label, multicenter studies investigated the incidence of side effects in patients treated with AZA vs. MMF.[35,36]<br><br>Palmer et al. reported no significant difference in terms of CMV infections and disease between both groups (p=0.24).[36] Similarly, McNeil's group observed no significant difference between the two groups in terms of opportunistic infections (61% in MMF and 67% in AZA) and sepsis (15% in MMF and 6% in AZA). They also found similar rates of abnormal kidney function (11% in MMF and 16% in AZA) and incidence of malignancy (12% in MMF and 13% in AZA).[35]<br><br>Given the limited sample size and follow-up duration (6-36 months), as well as substantial crossover in treatment allocation the overall level of evidence was very low.	Drug discontinuation occurred more commonly in the MMF group (not significant, p=0.19), mainly for leukopenia and gastrointestinal problems.[36] On the other hand, in a recently published retrospective study, medication interruption was higher in AZA versus MMF (HR 1.47; 95% CI 1.04–2.08; p=0.026).[40]<br><br>Several retrospective studies were included that provided data on side effects of different immunosuppressive regimens.<br><br>The study by Zuckermann et al. suggested a similar distribution of side effects, with a tendency for more frequent gastrointestinal complaints in MMF patients and more anaemia in the AZA group.[14] Bhorade's and Erdmann's studies found no differences in infection rate between the two groups.[20,37] Erdmann et al. described a similar rate of malignancy and diabetes.[20] On the contrary, Speich et al. described less CMV-related disease in the MMF group, where, however, gastrointestinal toxicity was more frequent.[38]<br><br>The choice between AZA and MMF is often based on the clinician's experience and the center's policy. Both drugs are associated with known side effects (e.g., infections, (skin) malignancy, gastrointestinal side effects, hepatitis…), some of which are more pronounced in one of the drugs. Patients on anti-proliferative agents may also have lower immune responses to vaccine antigens.<br><br>Patients ought to receive complete blood examinations routinely and should be monitored for gastrointestinal complaints, infections and malignancy, especially skin carcinomas. MMF requires additional caution regarding reproductive health, as it has been linked to birth defects.<br><br>**Pharmacogenetics**<br>The U.S. Food and Drug Administration advises TPMT genotyping or phenotyping before starting AZA. Individuals with reduced or absent TPMT activity are at high risk of severe, potentially life-threatening bone marrow suppression when treated with standard doses of azathioprine. [44,45]<br><br>MMF is a prodrug that is converted to mycophenolic acid (MPA), which is then metabolized primarily by UTG. Polymorphisms in UGT genes may affect MPA exposure, leading to variability in drug efficacy and toxicity. Individualized dosing based on pharmacogenetic profiling could be a future strategy to optimize MMF therapy, especially in transplant patients. Unlike TPMT testing for AZA, routine pharmacogenetic testing for MMF is not yet standard practice.[42]

		**CLINICAL EXPERIENCE**<br>Clinical experience shows that MMF is associated with more gastrointestinal side effects, while AZA is linked to higher rates of myelotoxicity. Most centers have transitioned to MMF as part of first-line triple immunosuppressive therapy, with the impression of generally favorable clinical outcomes.<br>Dosing may also considerably vary among patients and should probably be tailored to subjective tolerance, lymphoyte counts and/or AUC measurement. Trough level measurement is not recommended, and doses can be spread over the day (i.e., TID instead of BID administration) to allow better GI tolerance and higher daily doses. Enteric-coated mycophenolate sodium (EC-MPS), which delays the release of MPA until it reaches the small intestine, has been linked to significant improvements in gastrointestinal symptoms, health-related quality of life, and psychological well-being in case series involving various solid organ transplant recipients. While EC-MPS may reduce the need for dose reductions, interruptions, or discontinuation due to gastrointestinal side effects, it may not be locally available and has not demonstrated any proven advantage over MMF in preventing CLAD.<br><br>From clinical experience, we want to note that switching between drugs, for example from MMF to AZA due to fertility concerns, can be associated with a risk of developing de novo donor-specific antibodies and an increased risk of acute cellular rejection. A recent study assessing the effects of MMF dose modification (i.e., either dose reduction or discontinuation) within 36 months showed that 52% patients with MMF dose modifications experienced acute cellular rejection at any time within 36 months from transplant compared to 29% in patients without a MMF dose modification. There were 115 treated acute cellular rejection episodes among the 102 patients with a MMF dose modification compared to 5 episodes among 7 patients who underwent no MMF dose modification.[46]<br><br>**EVIDENCE FROM OTHER ORGANS**<br>**OTHER SOT EXPERIENCES/DATA**<br>(1B kidney, heart, lung; 2C pancreas; 1D intestine) . Azathioprine may be used in place of MPA in those intolerant to MPA products, such as gastrointestinal toxicity, that require an antimetabolite.[43]
CERTAINTY OF EVIDENCE<br>What is the overall certainty of the evidence of effects?		
Judgement	Research evidence	Additional considerations

o Very low<br>**X Low**<br>o Moderate<br>o High<br>o No included studies	The overall certainty of evidence is low due to downgrading for overall risk of bias and imprecision due to lack of statistical power and/or wide confidence intervals (crossing the null effect).	It is challenging to obtain strong scientific evidence on the preventive effect of AZA versus MMF in the development of CLAD. As already stated for PICO 1, lung transplantation is a rare entity and the availability of large randomized controlled trials is very limited. There might also be significant heterogeneity across centres in terms of immunosuppressive treatments and clinical practice for follow up.<br><br>Furthermore, even within the same centre, many patients may have their immunosuppressive regimen changed or modulated over time due to the onset of side effects (e.g., hepatotoxicity from AZA) or clinical reasons such as persistent acute rejection. Some patients may also experience temporary discontinuation of antiproliferative therapy due to difficult-to-control or severe infectious diathesis. These factors introduce significant variability in treatment exposure, making it harder to draw firm conclusions about the long-term impact of a specific antiproliferative agent on CLAD prevention.

VALUES
Is there important uncertainty about or variability in how much people value the main outcomes?

Judgement	Research evidence	Additional considerations
o Important uncertainty or variability<br>o Possibly important uncertainty or variability<br>**X Probably no important uncertainty or variability**<br>o No important uncertainty or variability	No included studies.	Since CLAD deeply affects patients' survival, both healthcare professionals and patients share the common goal of reducing its incidence and impact.<br><br>Qualitative studies and patient-reported outcomes consistently show that avoiding chronic rejection and preserving long-term graft function are top priorities.[8,9] Patients are generally willing to accept mild side effects and additional follow-up if there is even a small chance of reducing the risk of CLAD. Preferences appear consistent, with little variation across individuals.<br><br>Quality of life and other relevant patient-reported outcome measures are often not included in studies and we recommend taking this into account in future studies.

BALANCE OF EFFECTS
Does the balance between desirable and undesirable effects favor the intervention or the comparison?

Judgement	Research evidence	Additional considerations
o Favors the comparison<br>o Probably favors the comparison<br>**X Does not favor either the intervention or the comparison**<br>o Probably favors the intervention<br>o Favors the intervention	There seemed to be no significant difference in terms of effectiveness for these two immunosuppressive regimens and the safety profiles of these drugs also appeared to be acceptable for both.<br><br>Data from the ISHLT transplant registry indicated that MMF is the most commonly used antiproliferative agent following lung	In clinical practice, the choice of MMF versus AZA often depends on the patient's side effect profile, clinician's experience and preference and center's policy. For example, in patients with persistent gastrointestinal symptoms, AZA might be preferred. Whereas MMF might be preferred in patients with or prone to

○ Varies<br>○ Don't know	transplantation.[33] A survey on CLAD management showed that over 80% of lung transplant centers use MMF as the first-line antimetabolite.[10] Although MMF is currently more often used in clinical practice, there is no clear evidence demonstrating improved effectiveness of MMF over AZA.<br><br>Compared to MMF, AZA seemed associated with higher rejection rates in some studies, which may increase the long-term risk of CLAD, although no good long-term data or comparative studies were available to confirm this. Similarly, in other solid organ transplant experience, MMF appeared to be linked to lower rates of rejection. Therefore we were slightly in favor of MMF over AZA.	leukopenia as treatment-related leukopenia tends to be more frequently reported in AZA.
**RESOURCES REQUIRED**<br>How large are the resource requirements (costs)?		
Judgement	Research evidence	Additional considerations
○ Large costs<br>○ Moderate costs<br>**X Negligible costs and savings**<br>○ Moderate savings<br>○ Large savings<br>○ Varies<br>○ Don't know	No studies included.	Based on clinical experience, resource implications differ as the costs of medications vary between countries. The panel assumes the costs related to the use of both AZA and MMF are relatively feasible, but it depends on local availability, reimbursements/ insurance.<br><br>The administration of both AZA or MMF is normally part of standard immunosuppressive regimen in lung transplant centers, meaning no additional resources are required for their routine utilization.<br><br>Additional resources required for pharmacotyping can be considered negligible.
**CERTAINTY OF EVIDENCE OF REQUIRED RESOURCES**<br>What is the certainty of the evidence of resource requirements (costs)?		
Judgement	Research evidence	Additional considerations
○ Very low<br>○ Low<br>○ Moderate<br>○ High<br>**X No included studies**	No studies included.	
**COST-EFFECTIVENESS**<br>Does the cost-effectiveness of the intervention favor the intervention or the comparison?		
Judgement	Research evidence	Additional considerations
○ Favors the comparison<br>○ Probably favors the comparison<br>○ Does not favor either the intervention or the comparison<br>○ Probably favors the intervention<br>○ Favors the intervention<br>○ Varies<br>**X No included studies**	No studies included.	No cost-effectiveness studies either for AZA or MMF in the lung transplant population are available. Results from other studies (e.g., from connective tissue diseases patients) are not representative for our population because the underlying disease mechanism is different.<br><br>Given the high clinical and economic burden associated with CLAD, the use of these drugs in

		order to prevent the latter is likely to be cost-effective, but formal health economic analyses are needed to confirm this assumption.
EQUITY<br>What would be the impact on health equity?		
Judgement	Research evidence	Additional considerations
o Reduced<br>o Probably reduced<br>**X Probably no impact**<br>o Probably increased<br>o Increased<br>o Varies<br>o Don't know	No studies included.	Both AZA and MMF are widely available worldwide, also given the multitude of indications they are used for.
ACCEPTABILITY<br>Is the intervention acceptable to key stakeholders?		
Judgement	Research evidence	Additional considerations
o No<br>o Probably no<br>o Probably yes<br>**X Yes**<br>o Varies<br>o Don't know	No studies included.	Both AZA and MMF are widely used in other chronic diseases, both pulmonary and systemic, such as connective tissue disease. Both interventions are accepted and are generally considered safe and well-tolerated, although their safety profile and tolerability may vary among the different indications for use.<br><br>In the context of lung transplantation, both patients and healthcare professionals are familiar with their use, and concerns related to safety or adherence are limited, provided close clinical and instrumental follow-up is carried out and the correct course of action is taken in case side effects occur. Patients ought to receive complete blood examinations routinely and should be monitored for gastrointestinal complaints, infections and malignancy, especially skin carcinomas. MMF requires additional caution regarding reproductive health, as it has been linked to birth defects.<br><br>Given the high clinical relevance of preventing CLAD and the limited choice of alternative prophylactic strategies, the use of these drugs is considered acceptable for both healthcare providers and the patients.
FEASIBILITY<br>Is the intervention feasible to implement?		
Judgement	Research evidence	Additional considerations
o No<br>o Probably no<br>o Probably yes<br>**X Yes**<br>o Varies<br>o Don't know	No studies included.	Initiation of both AZA and MMF in adult lung transplant recipients is considered highly feasible, but might be subject to local availability and prescription regulations, which may differ across countries.<br><br>AZA and MMF can be given via intravenous route or orally, and can be initiated even before

		transplant (as per local protocol for induction); patients are generally hospitalized when they receive their first doses, and this allows closer monitoring. Some patients may also have received AZA and/or MMF before being transplanted as part of treatment for their primary pulmonary disease, e.g., connective tissue disease, hypersensitivity pneumonitis.<br><br>The standard dosage and modulation may vary based on the immunosuppressive protocol of the local transplant center.<br><br>No TDM is currently recommended for either of these drugs.[47]<br><br>No specialised monitoring is required beyond standard post-transplant care. Given its widespread availability, low cost, and clinician familiarity, the use of AZA or MMF does not pose logistical challenges and is readily implementable across transplant centres.

TYPE OF RECOMMENDATION				
Strong recommendation against the intervention	Conditional recommendation against the intervention	No recommendation against the intervention or in favor of the intervention	Conditional recommendation for the intervention	Strong recommendation for the intervention
☐	☐	☒	☐	☐

RECOMMENDATION

- In adult lung transplant recipients, we suggest either mycophenolate mofetil or azathioprine can be used to prevent the onset of CLAD. (Conditional recommendation for either the intervention or the comparison, low certainty of evidence)

REMARKS

There may be specific patient subgroups in which selecting one agent over the other is clinically justified. For example, in patients planning pregnancy, azathioprine (AZA) is preferred due to the teratogenic potential of mycophenolate mofetil (MMF). The choice of cell cycle inhibitor may also be influenced by individual susceptibility. AZA may be preferred in patients susceptible to or with persistent gastrointestinal side effects due to mycophenolic acid. Conversely, in patients with or susceptible to myelotoxicity, MMF might be preferred. Similarly, patients with gout requiring treatment with allopurinol (which may cause a serious drug interaction with AZA) may benefit from MMF. Finally, patients with genetic mutations in the thiopurine S-methyltransferase (TPMT) gene should avoid AZA (when homozygous deficient) or require reduced dosing (when heterozygous) as AZA treatment may induce severe, potentially fatal, bone marrow suppression by accumulation of toxic metabolites (6-thioguanine nucleotides) in these patiens, which does not occur when treated with MMF.

JUSTIFICATION

Based on the available evidence, effectiveness of these two immunosuppressive regimens did not differ significantly and the safety profiles of both drugs were acceptable. Therefore, our working group did not suggest favoring one immunosuppressive agent over the other. Overall, a change over time has been noted with more frequent use of MMF, and the Task Force also noted a slight clinical preference for mycophenolate, mainly because of the perceived lower risk of acute cellular rejection and antibody-mediated rejection.

SUBGROUP CONSIDERATIONS

There may be specific patient subgroups in which selecting one agent over the other is clinically justified. For example, in patients planning pregnancy, AZA is preferred due to the high teratogenic potential of MMF. The choice of antimetabolite may also be influenced by individual susceptibility. AZA might be preferred in patients susceptible to or with persistent gastrointestinal side effects due to MMF. Conversely, in patients with or susceptible to myelotoxicity, MMF might be preferred. Similarly, patients with gout requiring treatment with allopurinol (which has a dangerous interaction with AZA) may benefit from MMF.

IMPLEMENTATION CONSIDERATIONS

Azathioprine is included in the 2025 WHO Model List of Essential Medicines, whereas MMF is not. Nonetheless, MMF is generally considered more manageable in clinical practice. The U.S. Food and Drug Administration recommends TPMT analysis prior to initiating AZA therapy, due to the risk of severe myelotoxicity in individuals with TPMT deficiency. Prescribers should be aware of the interaction between cyclosporine and MMF; cyclosporine may decrease the blood levels of MMF substantially due to cyclosporine-induced inhibition of the enterohepatic recirculation of MMF, necessitating higher doses to achieve the same area under the curve dose level, by inhibiting its enterohepatic recirculation.

FURTHER RESEARCH SUGGESTIONS

Although randomized controlled trials in this area are unlikely to be organized, future research could still benefit from long-term real-life data regarding the use of different antiproliferative drugs in lung transplant recipients.
One relevant outcome for these studies could be the development of donor-specific antibodies in patients treated with AZA versus MMF. Additional areas of investigation may include:

- Identification of the optimal dosage, potentially influenced by pharmacogenetic factors beyond lymphopenia alone.
- Definition of patient subgroups (e.g., those with telomeropathies or other groups) in whom one agent may be preferred over the other.
- Long-term effects associated with each antimetabolite drug.
- Timing and management of MMF discontinuation in the context of infectious events and its potential impact on CLAD incidence.
- The possible need to interrupt antiproliferative agents around the time of vaccination.

PICO question 4

In adults with (progressive) CLAD, should extracorporeal photopheresis be used?

PROBLEM<br>Is the problem a priority?		
Judgement	Research evidence	Additional considerations
○ No<br>○ Probably no<br>○ Probably yes<br>**X Yes**<br>○ Varies<br>○ Don't know	Chronic lung allograft dysfunction (CLAD) is thought to be a correlate of chronic alloimmune rejection after lung transplantation, severely limiting survival. There are no effective treatments except for re-transplantation in strictly selected cases. CLAD affects about one third of lung transplant recipients within five years and presents with different phenotypes, including bronchiolitis obliterans syndrome (BOS), with an obstructive spirometric pattern, or restrictive allograft syndrome (RAS), which is associated with a restrictive pulmonary function test and radiological opacities. Mixed or undefined cases of CLAD also exist. CLAD leads to progressive, irreversible lung function decline, culminating in graft failure and increased mortality. Patients affected by the RAS phenotype have a worse prognosis. CLAD remains an important cause of death beyond the first year after transplant. The primary goal of treatment is to prevent further functional decline, graft loss, and mortality.<br>Despite its impact, treatment options for CLAD are limited and inconsistent, with no universally accepted strategy. This lack of consensus underscores the urgent need for clearer guidance.	Extracorporeal photopheresis (ECP) is an immunomodulatory therapy, involving leukapheresis, ex vivo exposure of leukocytes to 8-methoxypsoralen and UVA light, and reinfusion, that aims to induce immunological tolerance and reduce allograft injury—primarily in CLAD cases refractory to first-line treatments.<br>Following first-line therapies, ECP is a second-line option offered in select transplant centers with the necessary expertise, infrastructure and local reimbursement. Treatment typically involves multiple cycles over several months, followed by tapering based on clinical response. The total number of cycles can vary widely, with patients often receiving long-term maintenance therapy.<br>ECP is performed using either UVAR® or CELLEX® systems, which differ in processing speed and efficiency. Additionally, two procedural approaches exist: the Open system, where leukocytes are collected and treated in separate steps, and the Inline system, which integrates collection, treatment, and reinfusion in a closed loop.<br>There is no universal approach to ECP delivery, and protocols vary significantly between centers in terms of frequency, duration, and tapering strategies. Moreover, patient selection remains heterogeneous, often guided by clinical judgment rather than standardized criteria. The mechanisms underlying ECP's immunomodulatory effects in lung transplantation are still incompletely understood.
DESIRABLE EFFECTS<br>How substantial are the desirable anticipated effects?		
Judgement	Research evidence	Additional considerations
○ Trivial<br>**X Small**<br>○ Moderate<br>○ Large<br>○ Varies<br>○ Don't know	No randomized controlled trials on ECP in CLAD are currently available. Seven cohort studies evaluated 182 CLAD patients treated with ECP against 364 control patients.[48-54] None of the studies employed matched groups, and all were affected by substantial selection bias or bias by indication. In some cases[48,50], control groups had less advanced CLAD stages, while others[49,50,52,54] disproportionately included rapid decliners in the ECP arm. The presence of biased designs severely limits the strength of any conclusions, and due to considerable heterogeneity and methodological shortcomings, a meta-analysis could not be performed.<br><br>For the critical outcomes:	There were 13 additional non-controlled observational studies.[55-67] These studies demonstrated heterogeneity, varying sample sizes (8 to 631 patients), and considerable overlap of patients in the larger cohorts.<br><br>In the largest study with 631 patients, the overall survival rate was 86% at 6 months and 77% at 12 months.[66] In another large European study (373 patients), overall survival was 46% and graft survival 41% (54% deaths, 5% re-transplant) at a median follow-up of 92 months.[65] Benden et al. reported 50% graft survival over 10 years (14% re-transplantation, 33% died) and a median overall survival of 7 years.[55] 5-year overall survival in the study by Isenring et al. was 66% (6/9 patients) and graft survival 44%.[58] In the study by Morrell et al., median survival after ECP initiation was

	- Occurrence of graft loss due to CLAD was reported in 4 studies with no consistent results.[49,50,53,54] Jaksch et al. reported a 35% re-transplantation rate in the ECP group vs 15% in controls (p=0.004).[50] Note that the ECP group had more patients with a higher CLAD stage and more rapid decliners than the control group. Two other cohorts described 31-55% graft loss during follow-up, both in ECP and observation, despite significant cross-over to ECP in one of these studies.[49,54] The last cohort had too few events for comparison.[53]<br>- Overall survival was reported in 6 studies with varying results.[48-50,52-54] The majority of studies reported no significant differences in survival.[48,49,52,54] Jaksch et al. reported better survival in the ECP group vs controls (p=0.046)[50] and Pecoraro et al. reported a significantly higher survival in the ECP group vs controls (155 vs 114 months; p=0.03) in a small study[53], but with very poor methodological quality and high risk of bias.<br>Regarding important outcomes, FEV1 slope/response, definition of the outcomes was highly variable. Some studies assessed lung function slope, others a 20% FEV1 drop as lack of response and others assessed >10% FEV1 decline rate/100 person months. Overall, 1 study[53] found a positive effect of ECP compared to control, 2 studies[51,52] found no differences, two studies[48,50] reported worse FEV1 decline in the ECP group and the last study[49] could not assess due to early death in a large proportion of the ECP group. Response rate in the controlled studies (usually defined as FEV1 stabilization and/or improvement) varied between 33 to 80%, which may be explained by differences in patient populations or CLAD stages, even within studies. For example: Del Fante et al. had 60% stable ECP vs. 70% stable controls, but with 58% of patients having CLAD stage 1 in ECP vs. 91% in control group. Also, overall treatment failure over time was 67% in ECP vs. 93% in controls in this study.[48]<br><br>There were no data on quality of life.	2.6 years.[61] Finally, in Gautschi et al., 61% of patients died at a mean of 15 months post-ECP.[64]<br><br>Studies focused mostly on (evolution of) graft function after vs. before ECP initiation. The largest study reported FEV1 stabilization and/or improvement in 62% at 6 months and 51% at 12 months.[66] Other studies reported FEV1 stabilization and/or improvement in 19%-80%.[57,60-63,66,67]<br><br>Subgroup analyses in some of the larger studies may help in defining a responder profile:<br>- Patients with a BOS phenotype responded significantly better than patients with a RAS phenotype.[52,57,66] On the other hand, Del Fante et al. found no impact of CLAD phenotype.[48]<br>- Patients with CLAD stages I/II responded significantly better than patients with stages III/IV.[50,58,65,66] Gautschi et al. had similar findings, though not significant.[64] Other studies did not find this correlation.[48,53,57]<br>- Rapid FEV1 decliners responded less in several studies[48,50,57,65,66] and others[49,64] reported a trend toward less response. In contrast, Karnes et al.[59] reported better response rates in rapid decliners, while Leroux et al.[51] found no impact.<br>A recent prospective randomized trial studied the preventive use of ECP in the early post-operative course (16 courses) and found a significantly lower incidence of CLAD at year 3 in the ECP group vs induction-free controls without ECP (85% vs. 71%, p=0.015).[68]<br><br>Multiple other types of bias were identified such as a logistical bias (only patients living close to facilities could undergo ECP) or a bias of co-intervention (patient under ECP are assessed more frequently). At last, in the absence of adequately paired control groups, the proper effect of ECP on FEV1 evolution could not be differentiated from natural CLAD course history. In conclusion, the true effectiveness of ECP in the treatment of established CLAD remains questionable and deserves further research. A randomized controlled trial on ECP in CLAD is ongoing in the UK (eCLAD trial, NCT05721079), which may provide stronger evidence. At long term, additional studies on the frequency and duration of ECP are also needed.
**UNDESIRABLE EFFECTS**<br>How substantial are the undesirable anticipated effects?		
Judgement	Research evidence	Additional considerations

○ Large<br>○ Moderate<br>**X Small**<br>○ Trivial<br>○ Varies<br>○ Don't know	There were 7 studies with a control group that reported on undesirable effects.[48-54]<br><br>Infections were assessed in 4 studies: 3 studies reported no difference in the infection burden between groups.[50,52,53] Jaksch et al. reported fewer CMV infections in the ECP group.[50] Del Fante et al. reported a decrease in bacterial and CMV infection post- vs. pre-ECP without comparison to controls.[48] Serious complications from ECP were rare in these comparative studies.<br><br>There were no data on serious adverse events, kidney function and incidence of malignancies.	Some other undesirable effects were reported in the 13 studies[55-67] without control groups:<br>- Venous access problems may lead to the need for a central line (although the true incidence is not well known) with treatable line-related infections in 13-14% of patients.[61,67]<br>- No signal for ECP-specific long-term toxicity was identified.<br>- Some studies reported no important undesirable effects despite long-term ECP with 10 years follow-up.[55,58]<br>- At mid/long term, the profile of malignancies reported by some studies seemed reassuring.[58,66,67]<br>- Nephrotoxicity is neither expected nor reported.<br>Given its specificities, the profile of side effects of ECP differs significantly on some points from other treatments used in CLAD:<br>- Venous access is an important problem and may limit access to the treatment. From experience of the group, the use of central lines differs widely from one center to another.<br>- Iron deficiency anemia is another side effect not well reported in the studies. From experience, iron levels should be followed and corrected.<br>- Fatigue in the days following treatment can also occur, known from experience and underreported in studies.<br>As ECP has mainly immunomodulatory – rather than immunosuppressive – effects, less infections may occur compared to other immunosuppressive treatments[66], and also drug-induced side effects are limited in ECP.<br><br>At last, quality of life and patient-reported outcome measures were not assessed, and this point is particularly missing in current studies. Including patient-reported outcomes in future studies would help to assess the balance between the negative effect of a time-consuming procedure vs. the expected positive effects.<br><br>ECP is a long-term treatment requiring repeated sessions that will impact the burden for the patient. It deserves clear explanations and discussion with the patient in order to take their preferences into account before ECP is initiated. Also, stopping protocols should be in place in case of ineffectiveness, to avoid futile, costly treatment continuation.
**CERTAINTY OF EVIDENCE**<br>What is the overall certainty of the evidence of effects?		
Judgement	Research evidence	Additional considerations
**X Very low**<br>○ Low<br>○ Moderate<br>○ High<br>○ No included studies	The level of certainty is very low due to downgrading because of risk of bias, mainly due to selection bias, inconsistency because of heterogeneity across studies, and imprecision due to insufficient sample sizes for precise estimates.<br><br>Studies were all observational, often single center and with a retrospective design.	More than 1,000 patients treated with ECP have been reported in the literature, with a substantial proportion demonstrating favorable trends following therapy. However, because all available studies are non-randomized, it remains uncertain whether these outcomes can be attributed to ECP itself or if similar disease trajectories might have occurred without intervention. The lack of adequate comparator groups

		significantly limits confidence in interpreting the impact of ECP on disease progression.<br><br>Since the first study was published over 25 years ago, variability across publications extends beyond study design to differences in concomitant or prior treatments—particularly regarding the choice of calcineurin inhibitors and the use of azithromycin—further complicating interpretation and comparison of outcomes.<br><br>Conducting large randomized controlled trials for ECP is highly complex due to several factors, including significant variability in treatment protocols across centers and uneven access to the technique between countries. Moreover, the nature of ECP makes blinding virtually impossible. Despite these challenges, a non-blinded RCT is currently underway in the UK, that will bring more robust evidence in a few years' time.
**VALUES**<br>Is there important uncertainty about or variability in how much people value the main outcomes?		
Judgement	Research evidence	Additional considerations
o Important uncertainty or variability<br>**X Possibly important uncertainty or variability**<br>o Probably no important uncertainty or variability<br>o No important uncertainty or variability	No included studies.	No study that we are aware of formally assessed patient preferences regarding the main outcomes. Patients and clinicians consistently place high value on preserving lung function, preventing graft loss and survival, but also quality of life is highly valued.<br><br>Quality of life and patient-reported outcome measures should be assessed in future studies. Because of that, there is currently possibly important uncertainty about the value of the main outcomes.
**BALANCE OF EFFECTS**<br>Does the balance between desirable and undesirable effects favor the intervention or the comparison?		
Judgement	Research evidence	Additional considerations
o Favors the comparison<br>o Probably favors the comparison<br>**X Does not favor either the intervention or the comparison**<br>o Probably favors the intervention<br>o Favors the intervention<br>o Varies<br>o Don't know	After reviewing the available data on desirable and undesirable effects of ECP in the treatment of CLAD, the Task Force concluded that the desirable effects of ECP probably outweigh the undesirable effects among adult lung transplant recipients.	ECP may be associated with a more favorable disease course, but current evidence remains highly uncertain. Numerous unknowns persist regarding the optimal recipient characteristics, including phenotype and FEV1 slope, as well as the ideal timing for initiating treatment. Early use in patients with mild BOS or slower decline profiles appeared promising, yet these observations are not definitive. Additionally, treatment strategies—particularly the duration and frequency of ECP courses—lack standardization and remain poorly understood. As such, drawing firm conclusions about the effectiveness and best use of ECP remains challenging and it remains unclear whether and to what extent the desirable effects outweigh the undesirable effects.<br><br>The responder profile-definition should be assessed and consolidated in any further study on ECP.
**RESOURCES REQUIRED**<br>How large are the resource requirements (costs)?		
Judgement	Research evidence	Additional considerations

**X Large costs**<br>o Moderate costs<br>o Negligible costs and savings<br>o Moderate savings<br>o Large savings<br>oVaries<br>o Don't know	No studies included.	The resource requirements for ECP are substantial, both in terms of financial costs and logistical demands. The procedure involves specialized equipment and staff, and repeated sessions over extended periods, all of which contribute to high treatment costs. Additional costs related to travel and accommodation must also be considered. If effective, it may on the other hand delay the need for further high-cost interventions (e.g., re-transplant). Depending on the availability of reimbursement, costs charged to the patient also vary. Because of often long-term continuation, costs are much higher than the annual spending on immunosuppressive agents or alternative CLAD treatments assessed in this document.
**CERTAINTY OF EVIDENCE OF REQUIRED RESOURCES**<br>What is the certainty of the evidence of resource requirements (costs)?		
Judgement	Research evidence	Additional considerations
o Very low<br>o Low<br>o Moderate<br>o High<br>**X No included studies**	No studies included.	General cost components—such as equipment, staffing, and repeated treatment sessions—are well recognized.
**COST-EFFECTIVENESS**<br>Does the cost-effectiveness of the intervention favor the intervention or the comparison?		
Judgement	Research evidence	Additional considerations
o Favors the comparison<br>o Probably favors the comparison<br>o Does not favor either the intervention or the comparison<br>o Probably favors the intervention<br>o Favors the intervention<br>o Varies<br>**X No included studies**	No studies included.	The cost-effectiveness of ECP remains uncertain due to limited comparative economic data. Effectiveness remains not definitively established. Resource demands are high, but improved survival and delayed re-transplantation in eligible patients may offset some costs.
**EQUITY**<br>What would be the impact on health equity?		
Judgement	Research evidence	Additional considerations
o Reduced<br>**X Probably reduced**<br>o Probably no impact<br>o Probably increased<br>o Increased<br>o Varies<br>o Don't know	No studies included.	The impact of ECP on health equity is potentially concerning.<br><br>Access to ECP varies across centers and countries, potentially increasing inequities in care. Despite access to oral immunosuppressive agents, azithromycin, most intravenous agents in the management of CLAD and even redo transplantation, access to an ECP center is variable from one country to another, and also from one region to another in the same country. In the present conditions, access to the procedure is very frequently unequal from one patient to another.<br><br>Access and reimbursement depend on the local healthcare system structure and vary from country to country.

		Access to ECP is limited to specialized centers, often located in urban or high-resource settings, which can disproportionately exclude patients from rural areas, lower-income communities, or regions with limited healthcare infrastructure. The high cost of treatment, combined with travel and accommodation expenses for those living far from adapted facilities, may further widen disparities in care. This is also seen in practice: in some comparative studies the control arms consisted largely of patients who were unable or unwilling to undergo ECP treatment.<br><br>Several centers have discontinued ECP programs in the past due to logistical challenges and/or financial constraints.[69] Moreover, the resource demands of long-term ECP protocols may place additional strain on healthcare systems, leading to other disparities.

ACCEPTABILITY
Is the intervention acceptable to key stakeholders?

Judgement	Research evidence	Additional considerations
o No<br>o Probably no<br>o Probably yes<br>o Yes<br>**X Varies**<br>o Don't know	No studies included.	The acceptability of ECP among key stakeholders is mixed. While some patients and clinicians see it as a possible option for treating CLAD, there is still uncertainty about effectiveness. For patients, the treatment can be demanding; it involves regular hospital visits and semi-invasive procedures, which may affect their willingness to undergo this treatment. Healthcare providers may also have concerns about the costs and logistics and the use of resources, especially in settings with limited resources. Overall, ECP is not widely accepted, and opinions about its use vary depending on context and experience.<br><br>ECP is also used in other settings in some countries (e.g., cutaneous malignancies, graft-versus-host disease after allogeneic stem cell transplantation) where it is considered safe. It is generally well tolerated, and there are high adherence and continuation rates where offered.[70]<br><br>Because of these findings, the Task Force's opinion is that acceptability varies at the moment.

FEASIBILITY
Is the intervention feasible to implement?

Judgement	Research evidence	Additional considerations
o No<br>o Probably no<br>o Probably yes<br>o Yes<br>**X Varies**<br>o Don't know	No studies included.	Because (long-term) use of unequally available, reimbursed, specific and costly equipment and trained personnel in a tertiary specialized unit, this intervention is less feasible to implement.<br><br>As stated before, the therapy requires highly specialized equipment and personnel and is not uniformly available nor reimbursed across healthcare systems. Geography can make adherence and travel logistics complicated.

		Altogether, the feasibility of widely implementing ECP seems more limited.

TYPE OF RECOMMENDATION				
Strong recommendation against the intervention	Conditional recommendation against the intervention	Conditional recommendation for either the intervention or the alternative	Conditional recommendation for the intervention	Strong recommendation for the intervention
☐	☐	☒	☐	☐

RECOMMENDATION

- In adults with (progressive) CLAD, we suggest either ECP or no ECP be used. (Conditional recommendation for either the intervention or the comparison, very low certainty of evidence)

REMARKS

JUSTIFICATION

The balance between small and not very well-established desirable effects, few severe side effects, but the high therapeutic and logistic burden for the patients justified that the group could not recommend for neither against the use of ECP in the management of CLAD.
The ongoing multicenter RCT in the UK (eCLAD) can highly influence the current statement, and this recommendation might need to be updated once these data become available.

SUBGROUP CONSIDERATIONS

There might be patient groups that respond better. From the data available now, there might be a higher response rate in BOS patients, non-rapid decliners, and/or patients with early CLAD stages, but those responder profiles also need to be consolidated by more data.

IMPLEMENTATION CONSIDERATIONS

High costs for each procedure, need for specific equipment, trained personnel, possible need of central line and travel logistics should be taken into account in the assessment of indication.
The high cost is associated with differences in reimbursement among different healthcare systems.

FURTHER RESEARCH SUGGESTIONS

The group suggests further research on the following issues, related to the use of ECP for established CLAD:

- Definition and prediction of response to treatment.
- Consolidation of subgroup analyses for the responder profile definition. These data, and others, are awaited from the ongoing eCLAD trial.
- Harmonization of practices regarding frequency and duration of ECP. In stable treated patient, a randomized trial addressing ECP continuation vs. discontinuation could be considered.
- Evaluation of new treatment targets: use of biomarkers, patient-reported outcome measures (including quality of life, burden for patients), acceptable or non-acceptable side effects/adverse events, evaluation of the need for central line.
- A reliable and comparative cost-effectiveness study.

In addition to data in the management of CLAD, more data are needed regarding ECP in prevention of CLAD.

PICO question 5

In adults with (progressive) CLAD, should anti-thymocyte globulin be used?

PROBLEM<br>Is the problem a priority?		
Judgement	Research evidence	Additional considerations
○ No<br>○ Probably no<br>○ Probably yes<br>**X Yes**<br>○ Varies<br>○ Don't know	Chronic lung allograft dysfunction (CLAD) is thought to be a correlate of chronic alloimmune rejection after lung transplantation, severely limiting survival. There are no effective treatments except for re-transplantation in strictly selected cases. CLAD affects about one third of lung transplant recipients within five years and presents with different phenotypes, including bronchiolitis obliterans syndrome (BOS), with an obstructive spirometric pattern, or restrictive allograft syndrome (RAS), which is associated with a restrictive pulmonary function test and radiological opacities. Mixed or undefined cases of CLAD also exist. CLAD leads to progressive, irreversible lung function decline, culminating in graft failure and increased mortality. Patients affected by the RAS phenotype have a worse prognosis. CLAD remains an important cause of death beyond the first year after transplant. The primary goal of treatment is to prevent further functional decline, graft loss, and mortality.<br>Despite its impact, treatment options for CLAD are limited and inconsistent, with no universally accepted strategy. This lack of consensus underscores the urgent need for clearer guidance.	Anti-thymocyte globulin (ATG) has been used since the 1990s for induction and refractory acute cellular rejection (ACR) treatment. Based on that, it has been considered as an option for BOS, and then CLAD treatment.<br><br>In Europe, based on a recent survey, ATG is used as a 2nd line treatment for CLAD in 8% of centres and as a 3rd line treatment in another 8% of centres.[10]
DESIRABLE EFFECTS<br>How substantial are the desirable anticipated effects?		
Judgement	Research evidence	Additional considerations
○ Trivial<br>**X Small**<br>○ Moderate<br>○ Large<br>○ Varies<br>○ Don't know	Eight retrospective observational studies investigated ATG as a treatment for CLAD and reported results on a total of 353 patients (10 to 108 patients per study).[71-78] Only one study provided graft survival comparison with a paired control ATG-free CLAD group in a sub-analysis.[77] No prospective randomized controlled trials nor metanalyses have been published.<br><br>Three studies[73,75,77] included data on graft survival: two[73,75] reported a graft survival of 77-80% at a median follow-up of 10-63 months. In a propensity-matched sub-analysis, median graft survival was 26 months in 50 ATG-treated patients vs. 49 months in 50 non-treated patients (p=0.021).[77] This difference in graft survival may be related to a more severe baseline condition in the ATG group and residual confounding despite propensity matching.	There was wide variability regarding length of follow-up, both in terms of survival (3-12 months) and FEV1 (3-63 months). There was also variability regarding time between CLAD diagnosis and treatment with ATG (1.9-17 months).<br><br>The rate of FEV1 decline as a predictor for ATG response was investigated with contradictory results. Dunn et al. described a trend toward better response in rapid decliners (p=0.078).[77] January et al. found no predictors of response.[72] Two studies[73,74] reported a non-significant trend toward better response in CLAD grade < 3; while two other studies[72,77] found no correlation.<br>No correlation between response and CLAD phenotype was reported in three studies.[72,73,77]<br><br>Studies also differed regarding the total target ATG dose and monitoring of subsequent lymphopenia as well as the type of ATG administered (equine vs. rabbit). The oldest

	Overall survival was 95% at 6 months[72], 90% at 10 months[75], 54% at 12 months[74] and 56% at 17 months[71], in heterogeneous cohorts.<br><br>Between 13-40% were responders when defined as FEV1 improvement. When considering improved or stable FEV1, response ranged between 23-52%.[71-74,76-78] When defined as a decreased rate of FEV1 decline of > 20%, response was noted in 40-65%.[73,77]<br><br>An update of the literature retrieved one more retrospective study with a control group (n=55 ATG, n=69 no ATG) that showed lower 1-year post-CLAD mortality but not significant (SHR 0.66, 95%CI 0.39-1.14, p=0.134).[79] Regarding rate of FEV1 decline, no difference was found post- vs. pre-ATG (-0,09 L/year, 95%CI -0,21-0,03) and compared with non-treated patients [−0.1481 L/year, 95% CI (−0.32-0.021). Although it needs to be said that no pre-defined lung function period was compared, but that the rate of decline three months after CLAD diagnosis to last follow-up was compared with the lung function evolution from transplant up to three months before CLAD diagnosis.<br><br>Quality of life was not assessed in any of the studies.	studies reported indifferently on equine or rabbit ATG.[75,76,78] (Snell, Kesten, Date) In more recent studies, rabbit ATG was most commonly used.[71-74,77]<br><br>ATG was used as a 2nd or 3rd line treatment for CLAD, with variability of previous treatments that could influence the results.<br><br>Indication of ATG was individualized and probably favored patients with worse prognosis. Moreover, it is difficult to know the effect size of this treatment since no comparison with a control and the natural disease course of CLAD is available.<br><br>In all studies, patients with documented ACR were excluded. However, some authors excluded any ACR[72] and some excluded ACR grade 2 and higher. ATG remains an option to consider in a CLAD patient with associated ACR.<br><br>The Task Force judged the desirable effect to be small given the lack of control groups in most studies; however, members' clinical experience suggests that ATG can sometimes stabilize lung function.
**UNDESIRABLE EFFECTS**<br>How substantial are the undesirable anticipated effects?		
Judgement	Research evidence	Additional considerations
o Large<br>**X Moderate**<br>o Small<br>o Trivial<br>o Varies<br>o Don't know	Regarding malignancy, between 9-19% of patients developed skin cancer and 7-8% other types of malignancy.[72,77] The incidence of PTLD ranged between 0-10%.[77,78]<br><br>Other undesirable outcomes, such as serious adverse events and impairment in kidney function were not assessed.	There is heterogenicity regarding the evaluation of general tolerance of the treatment: fever and chills were reported in 0 to 50% of patients. Snell et al.[75] and Margallo et al.[74] reported good tolerance with mild adverse events and Izhakian et al.[71] reported 4% of patients having an infusion-related reaction. However, January et al.[72] described higher prevalence of symptoms related to the infusion, including 22% serum sickness and 15% cytokine release syndrome. In clinical experience, severe infusion-related symptoms are rare; premedication with for example higher dose of corticosteroids, acetaminophen and/or an antihistamine may prevent some symptoms.<br><br>Kesten et al. used systematically antibiotic prophylaxis in colonized patients and reported no infectious complications.[78] In the other studies, incidence of bacterial and/or viral infections ranged between 12-35%, and both Kotecha et al. and Margallo et al. described serious infections in 8-14% of patients.[73,74]<br><br>Cytopenia were inconsistently reported. Severe leukopenia was reported in 4% by Izhakian et al.[71] and neutropenia in 14% by Margallo et al.[74] Differences may exist between studies regarding

		discontinuation of cell cycle inhibitor during ATG treatment.<br><br>Administration via a central line or a large bore IV is generally preferred given the risk of local irritation and thrombophlebitis.<br><br>Regarding safety, there is also data available on ATG in the setting of induction therapy. A systematic review of six small randomized controlled trials with high risk of bias found no clear harm from T-cell antibody induction (ATG, ALG, IL-2RA, alemtuzumab, OKT3) compared with no induction or with each other in the setting of lung transplantation.[80] ATG increases immunosuppressive load, yet retrospective studies showed no clear rise in PTLD risk in lung or other solid organ transplants.[81] Current expert opinion favor the lowest effective dose, with particular caution in EBV-seronegative and other high-risk recipients. Some studies have reported a more favorable infection profile for alemtuzumab compared with ATG in lung, heart, and kidney transplantation.[82,83]<br><br>Finally, from clinical experience, some centers have experienced that muscle weakness is more pronounced in patients treated with ATG.
**CERTAINTY OF EVIDENCE**<br>What is the overall certainty of the evidence of effects?		
Judgement	Research evidence	Additional considerations
**X Very low**<br>○ Low<br>○ Moderate<br>○ High<br>○ No included studies	The overall certainty of the evidence for ATG treatment for CLAD is rated as very low, because of downgrading due to risk of bias, inconsistency related to clinical and methodological heterogenicity across studies and imprecision, owing to small sample sizes and a limited number of studies.	Only retrospective observational studies with relatively small sample sizes and no control group were available.
**VALUES**<br>Is there important uncertainty about or variability in how much people value the main outcomes?		
Judgement	Research evidence	Additional considerations
○ Important uncertainty or variability<br>○ Possibly important uncertainty or variability<br>**X Probably no important uncertainty or variability**<br>○ No important uncertainty or variability	No included studies.	Although there are no studies, there is high certainty that most patients value CLAD prevention and, after CLAD is diagnosed, CLAD stabilization. Qualitative studies and patient-reported outcomes consistently show that avoiding chronic rejection and preserving long-term graft function are top priorities.[8,9]<br><br>In this case, there might be some concerns about potentially serious adverse events that have hampered the generalization of this treatment, together with the fact that the evidence regarding its benefits is scarce. Available data on the risk of PTLD and infections remain insufficient to provide clear guidance, as existing studies are limited by small sample sizes, heterogeneous dosing strategies, and

		inadequate follow-up. Larger, well-designed cohorts with standardized protocols and long-term monitoring are needed to better define these risks and inform clinical practice.
**BALANCE OF EFFECTS**<br>Does the balance between desirable and undesirable effects favor the intervention or the comparison?		
Judgement	Research evidence	Additional considerations
o Favors the comparison<br>o Probably favors the comparison<br>**X Does not favor either the intervention or the comparison**<br>o Probably favors the intervention<br>o Favors the intervention<br>o Varies<br>o Don't know	Based on the current evidence, the balance is neither in favor nor against the use of ATG for CLAD treatment. There is very low evidence of the expected effect, only retrospective observational series mostly without control groups, but they describe some positive effect stabilizing FEV1 decline in up to 50% of patients. Small cohorts, selection bias, heterogenicity of previous treatments and definition of response limit our ability to measure the estimated effect. On the other hand, there are some relevant adverse effects including infections and possibly malignancies, that hamper the recommendation.<br>Nevertheless, given the high clinical impact of CLAD and the lack of other treatments, ATG might be used in selected patients.	The conclusion of this Task Force aligns with what has been described as the current practice in Europe, where ATG is scarcely used as a 2nd- or 3rd-line treatment for CLAD.[10]
**RESOURCES REQUIRED**<br>How large are the resource requirements (costs)?		
Judgement	Research evidence	Additional considerations
o Large costs<br>**X Moderate costs**<br>o Negligible costs and savings<br>o Moderate savings<br>o Large savings<br>oVaries<br>o Don't know	No studies included.	The panel assumes the costs related to the use of ATG are relatively expensive as it necessitates some resources: hospitalization, IV lines, trained nurses and monitoring and treatment of potential complications.<br>Although outpatient administration of rATG has been reported occasionally in kidney transplantation as feasible and safe, it is not common practice in most centers.[84]
**CERTAINTY OF EVIDENCE OF REQUIRED RESOURCES**<br>What is the certainty of the evidence of resource requirements (costs)?		
Judgement	Research evidence	Additional considerations
o Very low<br>o Low<br>o Moderate<br>o High<br>**X No included studies**	No studies included.	
**COST-EFFECTIVENESS**<br>Does the cost-effectiveness of the intervention favor the intervention or the comparison?		
Judgement	Research evidence	Additional considerations

o Favors the comparison<br>o Probably favors the comparison<br>o Does not favor either the intervention or the comparison<br>o Probably favors the intervention<br>o Favors the intervention<br>o Varies<br>**X No included studies**	No studies included.	No cost-effectiveness studies for ATG in the CLAD population are available. Results from other studies in kidney transplantation are not representative for our population because ATG was used for other indications (mainly induction).
**EQUITY**<br>What would be the impact on health equity?		
Judgement	Research evidence	Additional considerations
o Reduced<br>o Probably reduced<br>**X Probably no impact**<br>o Probably increased<br>o Increased<br>o Varies<br>o Don't know	No studies included.	ATG is an old and well-known treatment option that is relatively widely available. The risk of inequity is most likely low. ATG might not be available and/or reimbursed in some countries.
**ACCEPTABILITY**<br>Is the intervention acceptable to key stakeholders?		
Judgement	Research evidence	Additional considerations
o No<br>o Probably no<br>**X Probably yes**<br>o Yes<br>o Varies<br>o Don't know	No studies included.	ATG is already used in different solid organ transplants where it is considered safe. In the setting of lung transplantation, substantial clinical experience also supports its use as an induction therapy.<br><br>Before treatment with ATG can be accepted more widely, more data on efficacy and safety are needed. It is a treatment option that is being used as induction therapy and for CLAD by some centers; the latter indicating that some clinicians believe it has some effectiveness and can help stabilize CLAD progression.<br><br>Patient and healthcare provider concerns about safety do not seem to pose substantial barriers to administration due to the high risk of CLAD.<br><br>The fact that it is an intravenous treatment that requires about one week of hospital admission may limit patient acceptability. However, taking into account concerns about the prognosis of progressive CLAD, it is often well understood by patients. Moreover, compared with other treatment options, such as ECP, the treatment time for ATG is short.
**FEASIBILITY**<br>Is the intervention feasible to implement?		
Judgement	Research evidence	Additional considerations
o No<br>o Probably no<br>o Probably yes<br>**X Yes**<br>o Varies	No studies included.	Administration of ATG requires some resources: hospitalization, IV lines, trained nurses and monitoring. ATG might not be available and/or reimbursed everywhere.

○ Don't know		Although outpatient administration of rATG has been reported occasionally in kidney transplantation as feasible and safe, it is not common practice in the majority of centers.[84]

TYPE OF RECOMMENDATION				
Strong recommendation against the intervention	Conditional recommendation against the intervention	No recommendation against the intervention or in favor of the intervention	Conditional recommendation for the intervention	Strong recommendation for the intervention
☐	☐	☒	☐	☐

RECOMMENDATION

- In adults with (progressive) CLAD, we suggest either using ATG or not using it. (Conditional recommendation for either the intervention or the comparison, very low certainty of evidence)

REMARKS

(You may provide additional remarks, to supplement the recommendation. These will appear just below the recommendation in the manuscript)

We suggest using ATG with caution in patients with a history of or risk for malignancy (e.g., recent malignancy, malignancy with a high risk of recurrence, EBV mismatch) or a history of infections (especially difficult-to-treat and fungal infections).

JUSTIFICATION

There are no high-quality data supporting the use of ATG in the management of CLAD, although available studies suggest some lung function stabilization and clinical experience also indicates potential benefit. The main known drawback is an increased risk of infections. Overall, it may be considered a treatment option for selected CLAD patients. It can also be considered in patients with CLAD and concomitant ACR.

SUBGROUP CONSIDERATIONS

IMPLEMENTATION CONSIDERATIONS

Treatment requires hospitalization and intravenous access.
Availability might be limited in some countries.
There is currently no clear guidance on the optimal duration of treatment and monitoring of treatment effects.

FURTHER RESEARCH SUGGESTIONS

Randomized controlled trials are needed to better define efficacy and safety.
Further evidence is required to clarify optimal dosing, treatment duration and appropriate monitoring strategies.
Specific data are also lacking for high-risk groups, such as EBV-mismatched recipients at increased PTLD risk, as well as for distinct clinical subgroups, including different CLAD phenotypes.

PICO question 6

In adults with (progressive) CLAD, should alemtuzumab be used?

PROBLEM<br>Is the problem a priority?		
Judgement	Research evidence	Additional considerations
o No<br>o Probably no<br>o Probably yes<br>**X Yes**<br>o Varies<br>o Don't know	Chronic lung allograft dysfunction (CLAD) is thought to be a correlate of chronic alloimmune rejection after lung transplantation, severely limiting survival. There are no effective treatments except for re-transplantation in strictly selected cases. CLAD affects about one third of lung transplant recipients within five years and presents with different phenotypes, including bronchiolitis obliterans syndrome (BOS), with an obstructive spirometric pattern, or restrictive allograft syndrome (RAS), which is associated with a restrictive pulmonary function test and radiological opacities. Mixed or undefined cases of CLAD also exist. CLAD leads to progressive, irreversible lung function decline, culminating in graft failure and increased mortality. Patients affected by the RAS phenotype have a worse prognosis. CLAD remains an important cause of death beyond the first year after transplant. The primary goal of treatment is to prevent further functional decline, graft loss, and mortality.<br>Despite its impact, treatment options for CLAD are limited and inconsistent, with no universally accepted strategy. This lack of consensus underscores the urgent need for clearer guidance.	Several centers have explored rescue immunomodulatory strategies, including alemtuzumab, to attenuate the rapid decline in lung function in CLAD patients. Given the high disease burden and absence of effective alternatives, evaluation of such interventions is considered a clinical priority.<br><br>Alemtuzumab, mainly used as an induction immunosuppressive agent at the time of transplantation, has been used scarcely as an option for CLAD treatment. In Europe, alemtuzumab is used as a 3rd-line treatment for CLAD in 8% of centers.[10]
DESIRABLE EFFECTS<br>How substantial are the desirable anticipated effects?		
Judgement	Research evidence	Additional considerations
o Trivial<br>o Small<br>o Moderate<br>o Large<br>o Varies<br>**X Don't know**	Three retrospective observational studies investigated alemtuzumab for CLAD and reported results on a total of 41 patients (10 to 17 patients).[52,85,86] One study also included a control group of 78 CLAD patients not treated with alemtuzumab.[52] There were no randomized control trials studying this option.<br><br>Reams et al. reported graft survival at 1 and 2 years of 69%.[86] Overall survival was 69% at 1 and 2 years[86] and 76% at 5 years[85]. When compared to a non-treated group, no significant survival difference was seen at 6–12 months.[52]<br><br>In the study by Reams et al., mean FEV1 did not improve, but BOS grade improved in 40% of patients, remained stable in 30% and worsened in 30%.[86] Moniodis et al. reported a slower FEV1 decline after alemtuzumab initiation. However, compared with no	Across available cohorts, alemtuzumab was associated with short-term attenuation of spirometric decline, particularly in patients with BOS and in early stages, but survival benefits were not demonstrated and effects beyond 3–6 months remain uncertain. 86% of patients in BOS 2-3 presented FEV1 decline >10% but only 30% of patients in BOS1. Only one study described the effects in RAS patients and there was no change in FEV1 slope at 1 and 6 months.[52] Interpretation is further complicated by concomitant therapies, such as azithromycin or anti-reflux interventions, and by the preferential use of alemtuzumab in patients with rapidly progressive disease, which introduces selection bias and limits causal inference. Alemtuzumab was also used as different lines of treatment for CLAD.<br><br>Further data are needed, as the true effect size is unknown. However, some Task Force centers report possible lung function stabilization, and it may

	treatment, FEV1 decline was only slower at 1 month and not at 3 and 6 months.[52] In Ensor et al., a >10% FEV1 increase was noted at 30 days, but this was followed by a 10% decline by day 120. Freedom from BOS progression was 53% at 6 months.[85]<br><br>Quality of life was not assessed.	represent a potential option in patients with prior donor-specific antibodies.
**UNDESIRABLE EFFECTS**<br>How substantial are the undesirable anticipated effects?		
Judgement	Research evidence	Additional considerations
○ Large<br>**X Moderate**<br>○ Small<br>○ Trivial<br>○ Varies<br>○ Don't know	Undesirable outcomes, including serious adverse events, impairment in kidney function and malignancies, were not assessed in the included studies.	Infections are an important risk when using antibodies against T cells and are highly prevalent, affecting >50% of patients in different studies[52,86] and up to 77%[85]. Infectious complications were not different when using alemtuzumab (50%) compared to ECP (82%) p=0.12, but in this study there was no comparison with non-treated patients.[52] Studies reported 10-30% of deaths related to infectious complications.[52,85,86]<br>This needs to be put in perspective as CLAD patients are at increased risk of infection, but real-world clinical experience also suggests a further increased risk with alemtuzumab. We considered this risk to be moderate, based on clinical experience.<br><br>Some studies in kidney transplant recipients have reported good long-term graft survival and function after either alemtuzumab or ATG therapy for severe acute T cell-mediated rejection or glucocorticoid-resistant rejection, but also report frequent infections and worse patient survival outcomes compared to patients that did not require alemtuzumab treatment.[82,87,88] Infections were not more frequent in alemtuzumab compared to ATG, in treated renal transplant patients.[82]<br><br>In a Vienna study using alemtuzumab induction, 44% of patients developed infections under prophylaxis (mostly viral; 27% hospitalized, 4% ICU), nearly half within six months, and 9% developed malignancies (2% PTLD, 7% non-PTLD).[89]<br><br>Although data do not show a clear increase in post-transplant malignancies with alemtuzumab in kidney or lung recipients, clinical experience supports cautious use in patients with a prior history of cancer.
**CERTAINTY OF EVIDENCE**<br>What is the overall certainty of the evidence of effects?		
Judgement	Research evidence	Additional considerations
**X Very low**<br>○ Low<br>○ Moderate<br>○ High<br>○ No included studies	The overall certainty of the evidence for alemtuzumab treatment for CLAD is rated as very low, based on the lowest certainty among critical outcomes. For graft loss and overall survival, the certainty was downgraded by one level due to risk of bias, related to the selection of participants and short follow-up; and by another level for imprecision due to few studies and participants. Moreover, for overall survival certainty was downgraded	CLAD can be considered as a rare disease, and so randomized controlled trials are difficult to perform, and data are in general scarce. Only three retrospective observational studies with small sample sizes were available, with only one study with a control group.

	another level due to inconsistency in the results with different effect estimates and use of different time points making it difficult to compare the results. For FEV1 the certainty was downgraded by one level due to overall risk of bias due to the selection of participants and by one level for imprecision due to few studies and participants.	

VALUES
Is there important uncertainty about or variability in how much people value the main outcomes?

Judgement	Research evidence	Additional considerations
○ Important uncertainty or variability<br>○ Possibly important uncertainty or variability<br>**X Probably no important uncertainty or variability**<br>○ No important uncertainty or variability	No included studies.	Although there are no studies, there is high certainty that most patients value CLAD prevention and, after CLAD is diagnosed, CLAD stabilization. Qualitative studies and patient-reported outcomes (Singer et al 2019; Kugler et al. 2021) consistently show that avoiding chronic rejection and preserving long-term graft function are top priorities.[8,9]<br><br>In this case, there might be some concerns about potentially serious adverse events that have hampered the generalization of this treatment, together with the fact that the evidence regarding its benefits is scarce. Specifically, there is an increased risk of infectious complications—even with prophylaxis—which can be potentially severe in up to 30% of patients, demanding careful consideration before use. (respiratory) Infections is an important outcome for patients to be included in future studies.

BALANCE OF EFFECTS
Does the balance between desirable and undesirable effects favor the intervention or the comparison?

Judgement	Research evidence	Additional considerations
○ Favors the comparison<br>○ Probably favors the comparison<br>**X Does not favor either the intervention or the comparison**<br>○ Probably favors the intervention<br>○ Favors the intervention<br>○ Varies<br>○ Don't know	Based on the current evidence, the balance of effect does not favor the use of alemtuzumab for CLAD treatment. Evidence for the expected effect is very limited, consisting only of retrospective observational studies, which reported a positive impact in stabilizing FEV1 decline in up to 70% of patients. Small cohorts, selection bias and heterogeneity of previous treatments limit our ability to measure this estimated effect. On the other hand, side effects are frequent with infections affecting more than 50% of patients. Although they are generally treatable, these studies reported 10-30% of deaths related to this complication.<br><br>Nevertheless, given the high clinical impact of CLAD and the lack of other treatments, alemtuzumab might be used in selected patients. The data support short-term attenuation of spirometric decline, particularly in patients with BOS, but survival benefits were not demonstrated yet.<br>Taking all factors into account, we decided for a neutral recommendation, not in favor but	Clinically, alemtuzumab seems to have some effect, and although infection risks are acknowledged, its use can be considered cautiously, underlining the need for further data.<br><br>The conclusion of this Task Force aligns with what has been described as the current practice in Europe, where alemtuzumab is scarcely used as a 3rd-line of treatment for CLAD.[10]

	also not against the use of alemtuzumab in the treatment of CLAD.	
**RESOURCES REQUIRED**<br>How large are the resource requirements (costs)?		
Judgement	Research evidence	Additional considerations
**X Large costs**<br>o Moderate costs<br>o Negligible costs and savings<br>o Moderate savings<br>o Large savings<br>oVaries<br>o Don't know	No studies included.	The panel assumes the costs related to the use of alemtuzumab are relatively expensive as it necessitates some resources: hospitalization, IV lines (although IM or SC administration is also possible), trained nurses and monitoring. Costs may vary between countries and might be an issue to implement the therapy. It is also necessary to consider the costs associated with managing adverse events. Although outpatient administration of alemtuzumab has been reported as feasible and safe in other indications like aplastic anemia, it is not common practice in the transplantation field.[90]
**CERTAINTY OF EVIDENCE OF REQUIRED RESOURCES**<br>What is the certainty of the evidence of resource requirements (costs)?		
Judgement	Research evidence	Additional considerations
o Very low<br>o Low<br>o Moderate<br>o High<br>**X No included studies**	No studies included	
**COST-EFFECTIVENESS**<br>Does the cost-effectiveness of the intervention favor the intervention or the comparison?		
Judgement	Research evidence	Additional considerations
o Favors the comparison<br>o Probably favors the comparison<br>o Does not favor either the intervention or the comparison<br>o Probably favors the intervention<br>o Favors the intervention<br>o Varies<br>**X No included studies**	No studies.	No cost-effectiveness observational studies for alemtuzumab in the CLAD population are available. Results from other studies in kidney transplantation are not representative for our population because alemtuzumab was used for other indications (mainly induction). In some countries like the UK, alemtuzumab is available for solid organ transplantation through a special access program on a compassionate basis.
**EQUITY**<br>What would be the impact on health equity?		
Judgement	Research evidence	Additional considerations
o Reduced<br>**X Probably reduced**<br>o Probably no impact<br>o Probably increased<br>o Increased<br>o Varies<br>o Don't know	No studies included.	Alemtuzumab is not widely available, but usually available through special programs like for compassionate use. It is an off-label agent used as an induction agent and for rejection episodes, but it is not considered a standard or readily accessible treatment.

		Availability is limited in parts of Europe, and there is a history of temporary shortages, which poses a potential challenge.

ACCEPTABILITY<br>Is the intervention acceptable to key stakeholders?		
Judgement	Research evidence	Additional considerations
○ No<br>○ Probably no<br>**X Probably yes**<br>○ Yes<br>○ Varies<br>○ Don't know	No studies included.	Before alemtuzumab can be more widely adopted, additional data on its efficacy and safety are needed. Some centers use it as an induction therapy and for CLAD, reflecting a clinical perception that it may help stabilize disease progression. Its acceptability is influenced by availability and potential side effects. In countries like the UK, alemtuzumab for solid organ transplantation can be accessed at no cost through special compassionate-use programs.[91]

FEASIBILITY<br>Is the intervention feasible to implement?		
Judgement	Research evidence	Additional considerations
○ No<br>○ Probably no<br>**X Probably yes**<br>○ Yes<br>○ Varies<br>○ Don't know	No studies included.	Implementation needs hospitalization. Premedication with oral or IV steroids is required, and analgesics are advised prior to infusion. Antibiotics and antivirals should be administered routinely to all patients throughout and following treatment according to the summary of product characteristics. In addition to IV administration, SC/IM administration is also possible. Laboratory monitoring is advised. Finally, availability may limit feasibility.

TYPE OF RECOMMENDATION				
Strong recommendation against the intervention	Conditional recommendation against the intervention	No recommendation against the intervention or in favor of the intervention	Conditional recommendation for the intervention	Strong recommendation for the intervention
☐	☐	☒	☐	☐

RECOMMENDATION

- In adults with (progressive) CLAD, we suggest either using alemtuzumab or not using it. (Conditional recommendation for either the intervention or the comparison, very low certainty of evidence)

REMARKS

Alemtuzumab should be used with caution in patients with a history of or risk for malignancy (e.g., recent cancer, malignancy with high-risk of recurrence, EBV mismatch) or a history of infections, particularly difficult-to-treat or fungal infections.

JUSTIFICATION

Alemtuzumab might stabilize lung function in some patients, but the true effect size remains unknown, and more data are needed. Its use is supported by limited clinical experience from select centers. The risk of infections is recognized and should be considered in decision-making.

SUBGROUP CONSIDERATIONS

We consider alemtuzumab a potential treatment option in CLAD patients with DSAs, given its broad immunosuppressive effects.

IMPLEMENTATION CONSIDERATIONS

Alemtuzumab's availability may be limited in some regions, which could affect access.
It is generally given intravenously, but SC or IM administration is also possible.
In addition, the costs associated with the drug and the management of potential adverse events should be carefully considered when planning its use.

FURTHER RESEARCH SUGGESTIONS

Randomized controlled trials are needed to provide more robust data on alemtuzumab's efficacy and safety, as well as optimal dosing and frequency of administration.
Additional studies should in our opinion also gather data on certain risk factors for adverse events (such as patients with EBV mismatch, given the risk of PTLD) and focus on specific subgroups, such as different CLAD phenotypes and the presence of DSAs.

PICO question 7

In adults with (progressive) CLAD, should antifibrotics be used?

PROBLEM<br>Is the problem a priority?		
Judgement	Research evidence	Additional considerations
o No<br>o Probably no<br>o Probably yes<br>**X Yes**<br>o Varies<br>o Don't know	Chronic lung allograft dysfunction (CLAD) is thought to be a correlate of chronic alloimmune rejection after lung transplantation, severely limiting survival. There are no effective treatments except for re-transplantation in strictly selected cases. CLAD affects about one third of lung transplant recipients within five years and presents with different phenotypes, including bronchiolitis obliterans syndrome (BOS), with an obstructive spirometric pattern, or restrictive allograft syndrome (RAS), which is associated with a restrictive pulmonary function test and radiological opacities. Mixed or undefined cases of CLAD also exist. CLAD leads to progressive, irreversible lung function decline, culminating in graft failure and increased mortality. Patients affected by the RAS phenotype have a worse prognosis. CLAD remains an important cause of death beyond the first year after transplant. The primary goal of treatment is to prevent further functional decline, graft loss, and mortality.<br>Despite its impact, treatment options for CLAD are limited and inconsistent, with no universally accepted strategy. This lack of consensus underscores the urgent need for clearer guidance.	It is well established that both the main phenotypes of CLAD (BOS and RAS) are associated with fibroproliferative features, which may be caused by increased release of growth factors, such as fibroblast growth factor, platelet-derived growth factor and vascular endothelial growth factor.[92-96]<br><br>Therefore, several groups have proposed the use of pirfenidone and/or nintedanib for CLAD treatment, given their antifibrotic mechanism of action.
DESIRABLE EFFECTS<br>How substantial are the desirable anticipated effects?		
Judgement	Research evidence	Additional considerations
o Trivial<br>o Small<br>o Moderate<br>o Large<br>o Varies<br>**X Don't know**	Two randomized trials[97,98] are currently available on the use of pirfenidone in lung transplant recipients; the results of one of these randomized trials, the EPOS (European Trial of Pirfenidone in BOS) study, had yet to be published at the time of our initial literature search. Unfortunately, no study of similar kind is available for nintedanib at the time of writing.<br><br>The STOP-CLAD study is a randomized trial of pirfenidone in patients with CLAD: only 23 patients were enrolled (12 pirfenidone, 11 placebo) instead of 60 as originally planned.[97] The study was terminated early because of an Institution wide pause on clinical research due to the COVID-19 pandemic.<br>This group reported that pirfenidone did not significantly attenuate spirometric or radiographic decline; in particular, they found that the change in FEV1 over the 24-week study period for patients treated with pirfenidone was −3.1% (95% CI −9.2% to +2.9%) vs. patients	In addition to our predefined outcomes, the randomized controlled trial also found no change in FVC: change in FVC for the pirfenidone group was −2.5% (95% CI −8.8% to +3.7%) vs. patients treated with placebo having an observed change of −4.6% (95% CI −10.8% to +1.6%; p = 0.62).[97] Interestingly, they did not find a significant difference between CLAD phenotypes (obstructive vs. restrictive), although these subgroups were too small to draw solid conclusions. A significant impact on FEV1 and FVC was noted in female patients treated with pirfenidone. However, this finding might be due to chance. Overall in the general interstitial lung disease (ILD) population, no clear difference in effect due to sex was noted.[99,100]<br><br>In addition to the randomized controlled trial, two cohort studies of 9 and 11 patients investigated the role of pirfenidone in lung transplant recipients with CLAD.[101,102]

<table>
<tr>
<td></td>
<td>treated with placebo having an observed change of −3.7% (95% CI −9.6% to +2.3%; p = 0.90). Importantly, effect on lung function was a secondary outcome in this trial.

The results of the EPOS trial[98] (European multicenter randomized controlled trial on pirfenidone over placebo in BOS after lung transplantation) were published at the time of our recommendation meeting. Pirfenidone did not show superiority over placebo and standard care in this exploratory trial and, therefore, it was not recommended for treating BOS. 48 patients were randomized to pirfenidone and 42 to placebo with both groups showing continued decline in FEV1 from baseline to week 26. Secondary endpoints (graft loss, death, re-transplantation) were similar between groups, as well as the incidence of serious adverse events.[98]

No data on graft loss, overall survival and quality of life is available.</td>
<td>Regarding overall survival and graft survival, Vos et al. reported that 45% died 14 (10-21) months after initiation of pirfenidone with a median 3-year overall survival after RAS diagnosis of 55%. 3 patients underwent re-transplantation after 11 (5-12) months of pirfenidone. Therefore, graft loss occurred in 73% of patients. Median graft loss-free survival after RAS diagnosis was 1.8 years.[101] In the study by Bennett et al., median overall survival was 221 days from the start of treatment.[102]

In the study of Vos et al., FEV1 decline reduced from 50 (28-101) 12 months pre-pirfenidone to 10 (-11-47) mL/month 12 months after pirfenidone initiation (p<0.05) in 6/11 evaluable patients, no significant difference at 3 and 6 months.[101] In the study by Bennett et al., FEV1 decline reduced from -45±41 to -13±34 mL/month in 3/9 evaluable patients with lung function data at 6 months (p=0.874). (2 stable FEV1 (1 BOS, 1 RAS), 1 decline (RAS)). There was no difference in FEV1 at 3 months (-50±42 in 6/9 patients with data).[102]
No significant CT/PET scan differences were reported.[101] Quality of life was not mentioned in these studies.

However, a silver lining in this field is that a number of trials are ongoing to establish whether antifibrotics might be useful to treat CLAD, in particular:
- PIRCLAD: a single-center study, based at UCSF, assessing safety and tolerability of pirfenidone in restrictive CLAD.
- INFINITx-BOS: a multicenter randomized controlled trial, based in France (8 centers), testing the efficacy of nintedanib in LTx recipients with BOS.

For BOS, there does not seem to be a signal that pirfenidone is effective. For RAS, however, we do not have sufficient data at the moment to draw any conclusions. This needs to be studied further first. There are also novel antifibrotic drugs that became available recently or that will become available (e.g., nerandomilast,[103]) and might be explored in the future as possible treatments for CLAD as well.
Inhaled antifibrotics are also being studied in the ILD population and it might be of interest for future research in RAS.[104]</td>
</tr>
<tr>
<td colspan="3">UNDESIRABLE EFFECTS
How substantial are the undesirable anticipated effects?</td>
</tr>
<tr>
<td>Judgement</td>
<td>Research evidence</td>
<td>Additional considerations</td>
</tr>
<tr>
<td>○ Large
○ Moderate
X Small
○ Trivial
○ Varies
○ Don't know</td>
<td>Two randomized controlled trial investigated serious adverse events of the use of pirfenidone in lung transplant recipients with CLAD; no evidence was available for nintedanib.

In the STOP-CLAD trial, no life-threatening side effects were observed. Five serious adverse events (i.e., not life- threatening, but requiring</td>
<td>In addition to serious adverse events, Combs et al. reported 33 adverse events (average 2.8 per patient) in the pirfenidone group vs. 35 adverse events (average 3.2 per patient, p=0.65) in the placebo group. The pirfenidone group had more gastrointestinal (19 vs. 9) and fewer infectious (1 vs. 7) adverse events than the placebo group (p=0.022). The number of adverse events that</td>
</tr>
</table>

	medical intervention) were observed, all of which were in the placebo group (these included four infections requiring antibiotics and one case of hyponatremia).[97] The incidence of serious adverse events was similar between groups in the EPOS trial.[98]	were likely to be attributed to the study medication by the blinded adjudicator was higher in the pirfenidone group (p = 0.003).[97]<br><br>In the study from the Leuven group, subjective intolerance (mainly anorexia and nausea) was described in more than half of the patients (six patients, 55%), who needed dose de-escalation.[101] On the contrary, the Siena group reported only one case of severe gastrointestinal side effects, requiring interruption of treatment.[102]<br><br>Antifibrotics have a quite well-known risk profile, known from the ILD population. Especially the gastrointestinal symptoms can sometimes be debilitating, requiring treatment discontinuation. This is something that needs to be discussed with the patient prior to treatment initiation. Besides the risk of gastrointestinal side effects, we know from the general ILD population that there is also a risk of phototoxicity and hepatotoxicity.<br>Finally, the trough levels of CNI need to be monitored when antifibrotic treatment is initiated due to the possibility of drug interactions.[101]
**CERTAINTY OF EVIDENCE**<br>What is the overall certainty of the evidence of effects?		
Judgement	Research evidence	Additional considerations
o Very low<br>**X Low**<br>o Moderate<br>o High<br>o No included studies	The certainty of evidence was low due to downgrading because of risk of bias and imprecision due to few studies and participants and a low number of events.	As already stated for other PICOs, it is very challenging to obtain strong scientific evidence in the context of lung transplantation, a rare entity with possible significant heterogeneity across centers in terms of immunosuppressive treatments and clinical practice for follow up.
**VALUES**<br>Is there important uncertainty about or variability in how much people value the main outcomes?		
Judgement	Research evidence	Additional considerations
o Important uncertainty or variability<br>**X Possibly important uncertainty or variability**<br>o Probably no important uncertainty or variability<br>o No important uncertainty or variability	No included studies.	Given the significant impact of CLAD on survival, both patients and clinicians aim to reduce its incidence and burden.<br><br>Since finding a cure for chronic rejection is surely a key priority, many patients are willing to tolerate mild/moderate side effects and increased monitoring for even a small improvement in their lung function and consequent quality of life. However, data are scarce, especially regarding patient-reported outcomes, and future studies should prioritize these aspects. Notably, gastrointestinal side effects of antifibrotic therapy (especially when they are given together with immunosuppressive drugs) are particularly important and must be carefully considered and discussed.

BALANCE OF EFFECTS<br>Does the balance between desirable and undesirable effects favor the intervention or the comparison?		
Judgement	Research evidence	Additional considerations
o Favors the comparison<br>o Probably favors the comparison<br>o Does not favor either the intervention or the comparison<br>o Probably favors the intervention<br>o Favors the intervention<br>o Varies<br>**X Don't know**	At present, there is insufficient scientific evidence to make a judgment on the risk-benefit ratio of introducing an antifibrotic treatment for CLAD. The Task Force group was not in favor of using pirfenidone in the management of BOS but would like to see more data in RAS before making a recommendation for the latter patient population.	From clinical experience the high burden of gastrointestinal toxicity, which may necessitate treatment reduction or discontinuation, needs to be taken into account. Of note, in the above-mentioned EPOS trial[98], the incidence of gastrointestinal side effects was similar between patients who received pirfenidone and those on placebo.
RESOURCES REQUIRED<br>How large are the resource requirements (costs)?		
Judgement	Research evidence	Additional considerations
o Large costs<br>**X Moderate costs**<br>o Negligible costs and savings<br>o Moderate savings<br>o Large savings<br>**X Varies**<br>o Don't know	No studies included.	Both pirfenidone and nintedanib are currently off-label drugs for the treatment of any form of CLAD.<br>The drug might therefore not be accessible everywhere and the cost of an off-label drug can vary significantly, with some studies showing that off-label treatments are more expensive on average than on-label ones.<br>Based on clinical experience, resource implications differ as the costs of medications vary between countries; finally, the off-label use of these molecules may require prior authorization from the Institution where the patient is treated. In some countries, nintedanib is available if RAS is classified under progressive pulmonary fibrotic disease.
CERTAINTY OF EVIDENCE OF REQUIRED RESOURCES<br>What is the certainty of the evidence of resource requirements (costs)?		
Judgement	Research evidence	Additional considerations
o Very low<br>o Low<br>o Moderate<br>o High<br>**X No included studies**	No studies included.	None
COST-EFFECTIVENESS<br>Does the cost-effectiveness of the intervention favor the intervention or the comparison?		
Judgement	Research evidence	Additional considerations
o Favors the comparison<br>o Probably favors the comparison<br>o Does not favor either the intervention or the comparison<br>o Probably favors the intervention<br>o Favors the intervention<br>o Varies<br>**X No included studies**	No studies included.	No cost-effectiveness studies for antifibrotics in the CLAD population are available.
EQUITY<br>What would be the impact on health equity?		

Judgement	Research evidence	Additional considerations
o Reduced<br>**X Probably reduced**<br>o Probably no impact<br>o Probably increased<br>o Increased<br>o Varies<br>o Don't know	No studies included.	No strong evidence is available.<br><br>Both pirfenidone and nintedanib are currently given "on label" only for ILD, and even in those cases strict regulation and monitoring are applied. Antifibrotic drugs are often not available for CLAD and therefore, equity might be affected.
**ACCEPTABILITY**<br>Is the intervention acceptable to key stakeholders?		
Judgement	Research evidence	Additional considerations
o No<br>o Probably no<br>o Probably yes<br>o Yes<br>**X Varies**<br>o Don't know	No studies included.	Given the high clinical relevance of finding an effective treatment for CLAD and the limited choice of alternative strategies, the experimental use of these drugs might be considered acceptable for both healthcare providers and patients, although concerns exist in terms of potential serious side effects. Antifibrotic therapy does not seem to be effective in the management of BOS, but it might be tried in patients with RAS.<br><br>Finally, thanks to a recent survey on the management of CLAD, involving 44 lung transplant centers in Europe, it became even clearer that these drugs are not commonly used at the moment (also due to relative lack of availability).[10]
**FEASIBILITY**<br>Is the intervention feasible to implement?		
Judgement	Research evidence	Additional considerations
o No<br>o Probably no<br>**X Probably yes**<br>o Yes<br>o Varies<br>o Don't know	No studies included.	Both pirfenidone and nintedanib are currently given "on label" only for ILD, and even in those cases strict regulation and monitoring are applied.<br>For these reasons, this intervention might be not very feasible to implement.

**TYPE OF RECOMMENDATION**				
Strong recommendation against the intervention	Conditional recommendation against the intervention	No recommendation	Conditional recommendation for the intervention	Strong recommendation for the intervention
☐	☒	☒	☐	☐

RECOMMENDATION

- In adults with (progressive) CLAD phenotype BOS, we suggest not using antifibrotic therapy with pirfenidone. (Conditional recommendation against the intervention, low certainty of evidence)
- In adults with (progressive) CLAD phenotype RAS, we suggest either using antifibrotic therapy or not using it. (Conditional recommendation for either the intervention or the comparison, low certainty of evidence)

REMARKS

JUSTIFICATION

Based on our review of the currently available scientific evidence, we do not suggest using antifibrotic therapies in BOS, as no meaningful signal of efficacy has been identified to date.
In contrast, we believe that additional data are needed before we can comment on their potential role in RAS.

SUBGROUP CONSIDERATIONS

We do not recommend using antifibrotic therapy in BOS patients given the lack of efficacy.
However, antifibrotic therapy might be considered in RAS patients, as it might be more effective in this CLAD phenotype, although more data are needed to better understand the effect size in this patient population.

IMPLEMENTATION CONSIDERATIONS

Lack of availability and reimbursement might limit implementation. In some countries, nintedanib can be prescribed for RAS if it is classified under progressive pulmonary fibrosis and/or as an off-label treatment.
Patients should be monitored for side effects, such as gastrointestinal side effects, hepatotoxicity and skin toxicity. Additionally, trough levels of calcineurin inhibitors should be monitored as drug interactions are possible.

FURTHER RESEARCH SUGGESTIONS

Depending on the expected results from the PIRCLAD study in patients with restrictive CLAD, we recommend further research in this CLAD population. Studies with other antifibrotics, such as nerandomilast, could be considered.

PICO question 8

In adults with (progressive) CLAD, should montelukast be used?

PROBLEM<br>Is the problem a priority?		
Judgement	Research evidence	Additional considerations
○ No<br>○ Probably no<br>○ Probably yes<br>**x Yes**<br>○ Varies<br>○ Don't know	Chronic lung allograft dysfunction (CLAD) is thought to be a correlate of chronic alloimmune rejection after lung transplantation, severely limiting survival. There are no effective treatments except for re-transplantation in strictly selected cases. CLAD affects about one third of lung transplant recipients within five years and presents with different phenotypes, including bronchiolitis obliterans syndrome (BOS), with an obstructive spirometric pattern, or restrictive allograft syndrome (RAS), which is associated with a restrictive pulmonary function test and radiological opacities. Mixed or undefined cases of CLAD also exist. CLAD leads to progressive, irreversible lung function decline, culminating in graft failure and increased mortality. Patients affected by the RAS phenotype have a worse prognosis. CLAD remains an important cause of death beyond the first year after transplant. The goal of immunosuppression is to prevent functional decline, graft loss, and mortality and to achieve a good quality of life.<br>Despite its impact, treatment options for CLAD are limited and inconsistent, with no universally accepted strategy. This lack of consensus underscores the urgent need for clearer guidance.	Montelukast works by blocking leukotriene D4 (LTD4) at the cysteinyl leukotriene receptor (CysLT1) in the airways. Leukotrienes are inflammatory mediators involved in bronchoconstriction, mucus production, airway edema, and eosinophil recruitment. By blocking their action, montelukast helps reduce airway inflammation and prevent bronchospasm.
**DESIRABLE EFFECTS**<br>How substantial are the desirable anticipated effects?		
Judgement	Research evidence	Additional considerations
**X Trivial**<br>○ Small<br>○ Moderate<br>○ Large<br>○ Varies<br>○ Don't know	Only one randomized, double-blind, placebo-controlled trial has tested montelukast in lung allograft recipients with BOS and slowly progressive loss of FEV1 despite prior azithromycin. In that proof-of-concept study (n=30; montelukast 15 vs. placebo 15), most patients had BOS stage 1–2 at inclusion.[105]<br><br>This randomized controlled trial comparing montelukast vs. placebo in lung transplant recipients found no significant difference in graft loss (re-transplantation or death at 2 years), with a risk ratio (RR) of 0.48 [95% CI 0.12–1.87], or mortality at the limited follow-up available (1 and 2 years).[105]<br><br>Overall, there was no significant difference on FEV1 (RR for FEV1 decline at 6 months	There were two additional observational studies, all from the same center.[106,107]<br><br>Observational evidence suggests that montelukast may confer clinically relevant benefits in patients with CLAD, particularly in those with BOS. In the largest retrospective cohort[107] from Leuven (n=153; 115 BOS, 38 RAS), treatment with montelukast after at least three months of azithromycin was associated with a significant attenuation of lung function decline (at 3 and 6 months (both p<0.0001)). At three months, 81% of patients demonstrated stable or improved FEV1, and benefits persisted, albeit attenuated, at six and 12 months. At 12 months, CLAD progression was reported in 39%. Responders had superior progression-free and overall survival compared to non-

	0.83, 0.32-2.15, p=0.71). However, a post-hoc subgroup analysis for BOS stage 1 reported significant reduction in FEV1 decline with montelukast (reported response 4/8 with montelukast vs. 0/8 with placebo).[105]<br><br>Regarding FEV1 improvement or stabilization at one year, results showed no difference between groups (RR 1.00; 95% CI 0.48–2.08).[105]<br><br>There were no data for quality of life.	responders (adjusted HR for death 2.816 in non-responders).[107]<br>A smaller comparative observational study (n=22) reported a substantially slower decline in FEV1 with montelukast (13 ml/month) compared to standard therapy (114 ml/month).[106]<br><br>Taken together, these data suggest that montelukast may stabilize lung function and may delay disease progression in selected CLAD patients. Notably, rapid decliners were less likely to respond in the study by Vos et al.[107] and were excluded in the randomized trial[105].<br><br>From data on pulmonary chronic graft-versus-host disease after allogeneic hematopoietic stem cell transplantation, we know that montelukast is recommended as part of the typical first-line "FAM" therapy, combining an inhalation corticosteroid, azithromycin and montelukast.[108] However, the magnitude of effect of montelukast itself was unclear here. One small study of 25 patients reported that all patients on montelukast had a stable (< 15% FEV1 decline) or improved lung function at 6 months. However, there was no control group to compare with.[109]
**UNDESIRABLE EFFECTS**<br>How substantial are the undesirable anticipated effects?		
Judgement	Research evidence	Additional considerations
o Large<br>o Moderate<br>**X Small**<br>o Trivial<br>o Varies<br>o Don't know	Undesirable effects in the randomized trial were limited.[105] The incidence of serious adverse events was similar (RR 0.83; 95% CI 0.44–1.56) and treatment discontinuation rates did not differ significantly (RR 1.00; 95% CI 0.24–4.21) between groups. Notably, serious adverse events were not pre-registered as an outcome in the protocol and it is unclear what definition was used. Likewise, the occurrence of respiratory infections was comparable in both groups (RR 0.80; 95% CI 0.38–1.70).[105]<br><br>The trial did not include renal outcomes and incidence of malignancy.	Across the observational cohorts, montelukast was generally well tolerated. In Vos et al., no severe adverse events were attributed to the drug; a minority of patients reported vivid dreams, but none discontinued therapy for this reason.[107] From clinical experience, we know that nightmares and other sleep disturbances can occur.<br><br>From its broader use in asthma and allergic disease, rare but recognized risks include neuropsychiatric adverse events. In March 2020, the U.S. Food and Drug Administration (FDA) strengthened the warnings on montelukast by requiring a Boxed Warning (the most prominent safety warning) regarding serious mental-health side effects. The FDA's decision followed a comprehensive review of post-marketing reports, observational data (including from the FDA Sentinel system), and published literature on neuropsychiatric adverse events.[110] Although these warnings derive

		largely from non-transplant populations, they apply to all uses and must be weighed in transplant recipients who often have complex neuropsychiatric vulnerabilities. The study of Vos et al.[107] reported no severe neuropsychiatric disorders and also from clinical experience, neuropsychiatric effects are not often seen. Other side effects are headaches and abdominal pain. Overall, the safety profile from observational lung transplant studies seemed acceptable, with a low likelihood of serious harm in the transplant population.<br><br>Prescribers should be aware of the risk of neurological adverse events, which can be severe. Therefore, the group debated between moderate and small undesirable effect. The group voted (taking into account COIs) and decided to go with small.
CERTAINTY OF EVIDENCE<br>What is the overall certainty of the evidence of effects?		
Judgement	Research evidence	Additional considerations
o Very low<br>**X Low**<br>o Moderate<br>o High<br>o No included studies	The certainty of evidence is low, as data are limited to a small single-center randomized controlled trial, with downgrading due to imprecision.	
VALUES<br>Is there important uncertainty about or variability in how much people value the main outcomes?		
Judgement	Research evidence	Additional considerations
o Important uncertainty or variability<br>o Possibly important uncertainty or variability<br>**X Probably no important uncertainty or variability**<br>o No important uncertainty or variability	No studies included.	Values and preferences likely favor interventions that maintain lung function and delay re-transplantation, with a high willingness among patients to accept therapies that might slow decline. No studies directly assessed patient values or preferences regarding montelukast in CLAD. However, given the high burden associated with CLAD progression and the strong link between FEV1 decline and survival, it is likely that patients and clinicians would place high value on even modest stabilization of lung function. Observational evidence that montelukast responders achieved better survival reinforces the perception that small gains may be meaningful in this context.[107] Still, given the Black Box warning, variability in how patients value neuropsychiatric risk is expected; shared decision-making is essential.

BALANCE OF EFFECTS
Does the balance between desirable and undesirable effects favor the intervention or the comparison?

Judgement	Research evidence	Additional considerations
o Favors the comparison<br>o Probably favors the comparison<br>**X Does not favor either the intervention or the comparison**<br>o Probably favors the intervention<br>o Favors the intervention<br>o Varies<br>o Don't know	Overall, montelukast showed no statistically significant advantage over placebo for graft function, safety, or infection outcomes, and results were limited by the small sample size and wide confidence intervals.<br><br>Montelukast showed limited but potentially meaningful benefit in some CLAD patients, particularly those with early-stage BOS. Evidence suggests it may slow FEV1 decline, stabilize lung function, and improve survival in patients who respond, while effects in RAS remain uncertain due to insufficient data. Benefits appear greatest in earlier CLAD stages and slowly progressive disease. While uncommon, the risk of neuropsychiatric effects (FDA black box warning) warrant caution and systematic monitoring. We suggest that a therapeutic trial of montelukast can be tried in carefully selected CLAD patients, especially BOS stage 1 with slowly progressive decline after azithromycin, provided vigilant safety monitoring and early discontinuation in non-responders.	Montelukast is currently not commonly used in clinical practice (26% of centers), according to a European report, suggesting many clinicians are currently not in favor of using it.[10]

RESOURCES REQUIRED
How large are the resource requirements (costs)?

Judgement	Research evidence	Additional considerations
o Large costs<br>o Moderate costs<br>**X Negligible costs and savings**<br>o Moderate savings<br>o Large savings<br>oVaries<br>o Don't know	No studies included.	No economic evaluations were identified. As a generic oral medication administered once daily without the need for therapeutic monitoring, montelukast entails negligible additional costs in most healthcare systems. The drug does not demand specialized infusion capacity or repeated hospital resources, in contrast to extracorporeal photopheresis or total lymphoid irradiation; the principal “costs” are clinical time for counseling and follow-up spirometry, with potential downstream costs if neuropsychiatric events require evaluation.<br><br>No differences among health care systems are expected due to the low cost and general availability of the therapy.

CERTAINTY OF EVIDENCE OF REQUIRED RESOURCES
What is the certainty of the evidence of resource requirements (costs)?

Judgement	Research evidence	Additional considerations
o Very low<br>o Low<br>o Moderate<br>o High<br>**x No included studies**	No studies included.	
**COST-EFFECTIVENESS**<br>Does the cost-effectiveness of the intervention favor the intervention or the comparison?		
Judgement	Research evidence	Additional considerations
o Favors the comparison<br>o Probably favors the comparison<br>o Does not favor either the intervention or the comparison<br>o Probably favors the intervention<br>o Favors the intervention<br>o Varies<br>**X No included studies**	No studies included.	No cost-effectiveness observational studies for montelukast in the CLAD population are available. Results from other studies (e.g., from the asthma population) are not representative for our population because the underlying disease mechanism is different.
**EQUITY**<br>What would be the impact on health equity?		
Judgement	Research evidence	Additional considerations
o Reduced<br>o Probably reduced<br>**X Probably no impact**<br>o Probably increased<br>o Increased<br>o Varies<br>o Don't know	No studies included.	Montelukast is relatively widely available in most settings and simple to implement within standard post-transplant care pathways.
**ACCEPTABILITY**<br>Is the intervention acceptable to key stakeholders?		
Judgement	Research evidence	Additional considerations
o No<br>o Probably no<br>**X Probably yes**<br>x Yes<br>o Varies<br>o Don't know	No studies included.	Treatment with montelukast is most likely acceptable. Montelukast is widely used in other respiratory conditions, with a well-known safety profile. Consensus recommendations for analogous conditions (pulmonary chronic graft-versus-host disease) list it as a first-line treatment.[108] Acceptability may be tempered by patients' and clinicians' awareness of the boxed warning; clear counseling improves acceptability.<br><br>So generally, treatment with montelukast is accepted by physicians after explaining the side effect profile. However, there is limited data on efficacy in our CLAD population and

		no overall effect is seen in the randomized trial.
**FEASIBILITY**<br>Is the intervention feasible to implement?		
Judgement	Research evidence	Additional considerations
o No<br>o Probably no<br>o Probably yes<br>**X Yes**<br>o Varies<br>o Don't know	No studies included.	Montelukast is administered orally once daily, requires no laboratory monitoring, is widely available and can be easily integrated into existing CLAD treatment pathways.

TYPE OF RECOMMENDATION				
Strong recommendation against the intervention	Conditional recommendation against the intervention	No recommendation against the intervention or in favor of the intervention	Conditional recommendation for the intervention	Strong recommendation for the intervention
☐	☐	☒	☐	☐

RECOMMENDATION

- In adults with (progressive) CLAD, we suggest either using montelukast or not using it. (Conditional recommendation for either the intervention or the comparison, low certainty of evidence)

REMARKS

Montelukast may be effective in cases of early BOS.

JUSTIFICATION

Overall, no effect of montelukast on FEV1 was seen in the randomized controlled trial, but some effectiveness was demonstrated in patients with early stages of BOS (BOS 1). Therefore, we suggest that montelukast can be initiated in these patients. In case of further progressive disease, we suggest discontinuing the treatment.

SUBGROUP CONSIDERATIONS

Montelukast may be effective in cases of early BOS. In patients with new-onset BOS 1, a trial of montelukast can be considered.

IMPLEMENTATION CONSIDERATIONS

The advantage of montelukast compared with some of the other treatments (e.g., ECP, ATG) is that it can be administered easily orally.
We suggest monitoring treatment response. In case of lack of response, we suggest discontinuation.
We suggest monitoring any effects on neurological and psychiatric symptoms and sleep pattern.

FURTHER RESEARCH SUGGESTIONS

The Task Force did not formulate specific future research recommendations for montelukast, as they believe priorities rather lie elsewhere. If further research is being performed, then best for early stages of CLAD.

References

1. Li D, Duan Q, Weinkauf J, et al. Azithromycin prophylaxis after lung transplantation is associated with improved overall survival. *J Heart Lung Transplant.* 2020;39(12):1426-1434.
2. Cristeto Porras M, Mora Cuesta VM, Iturbe Fernández D, et al. Early onset of azithromycin to prevent CLAD in lung transplantation: Promising results of a retrospective single centre experience. *Clin Transplant.* 2023;37(1):e14832.
3. Van Herck A, Frick AE, Schaevers V, et al. Azithromycin and early allograft function after lung transplantation: A randomized, controlled trial. *J Heart Lung Transplant.* 2019;38(3):252-259.
4. Vos R, Vanaudenaerde BM, Verleden SE, et al. A randomised controlled trial of azithromycin to prevent chronic rejection after lung transplantation. *Eur Respir J.* 2011;37(1):164-172.
5. Ruttens D, Verleden SE, Vandermeulen E, et al. Prophylactic Azithromycin Therapy After Lung Transplantation: Post hoc Analysis of a Randomized Controlled Trial. *Am J Transplant.* 2016;16(1):254-261.
6. Li H, Liu DH, Chen LL, et al. Meta-analysis of the adverse effects of long-term azithromycin use in patients with chronic lung diseases. *Antimicrob Agents Chemother.* 2014;58(1):511-517.
7. Spence CD, Vanaudenaerde B, Einarsson GG, et al. Influence of azithromycin and allograft rejection on the post-lung transplant microbiota. *J Heart Lung Transplant.* 2020;39(2):176-183.
8. Kugler C, Fischer S, Gottlieb J, et al. Health-related quality of life in two hundred-eighty lung transplant recipients. *J Heart Lung Transplant.* 2005;24(12):2262-2268.
9. Singer JP, Soong A, Chen J, et al. Development and Preliminary Validation of the Lung Transplant Quality of Life (LT-QOL) Survey. *Am J Respir Crit Care Med.* 2019;199(8):1008-1019.
10. Gottlieb J, Vos R, Jaksch P, et al. Chronic lung allograft dysfunction after lung transplantation: prevention, diagnosis and treatment in 44 European centres. *ERJ Open Res.* 2025;11(3).
11. Sheshadri A, Sacks NC, Healey B, Cyr P, Boerner G, Huang HJ. The healthcare resource utilization and costs of chronic lung allograft dysfunction following lung transplantation in patients with commercial insurance in the United States. *J Med Econ.* 2022;25(1):650-659.
12. Kapnadak SG, Morrell ED, Wai TH, et al. Variability in azithromycin practices among lung transplant providers in the International Society for Heart and Lung Transplantation Community. *J Heart Lung Transplant.* 2022;41(1):20-23.
13. Dellgren G, Lund TK, Raivio P, et al. Effect of once-per-day tacrolimus versus twice-per-day ciclosporin on 3-year incidence of chronic lung allograft dysfunction after lung transplantation in Scandinavia (ScanCLAD): a multicentre randomised controlled trial. *Lancet Respir Med.* 2024;12(1):34-44.
14. Zuckermann A, Klepetko W, Birsan T, et al. Comparison between mycophenolate mofetil- and azathioprine-based immunosuppressions in clinical lung transplantation. *J Heart Lung Transplant.* 1999;18(5):432-440.
15. Hachem RR, Yusen RD, Chakinala MM, et al. A randomized controlled trial of tacrolimus versus cyclosporine after lung transplantation. J Heart Lung Transplant. 2007 Oct;26(10):1012-8.
16. Treede H, Glanville AR, Klepetko W, et al. Tacrolimus and cyclosporine have differential effects on the risk of development of bronchiolitis obliterans syndrome: results of a prospective, randomized international trial in lung transplantation. In: *J Heart Lung Transplant.* Vol 31. United States: © 2012 International Society for Heart and Lung Transplantation. Published by Elsevier Inc; 2012:797-804.
17. Griffith BP, Bando K, Hardesty RL, et al. A prospective randomized trial of FK506 versus cyclosporine after human pulmonary transplantation. *Transplantation.* 1994;57(6):848-851.
18. Keenan RJ, Konishi H, Kawai A, et al. Clinical trial of tacrolimus versus cyclosporine in lung transplantation. Ann Thorac Surg. 1995 Sep;60(3):580-4; discussion 584-5. doi: 10.1016/0003-4975(95)00407-C.
19. Treede H, Klepetko W, Reichenspurner H, et al. Tacrolimus versus cyclosporine after lung transplantation: a prospective, open, randomized two-center trial comparing two different immunosuppressive protocols. In: *J Heart Lung Transplant.* Vol 20. United States2001:511-517.
20. Erdman J, Wolfram J, Nimke D, et al. Lung Transplant Outcomes in Adults in the United States: Retrospective Cohort Study Using Real-world Evidence from the SRTR. *Transplantation.* 2022;106(6):1233-1242.
21. Reichenspurner H, Kur F, Treede H, et al. Optimization of the immunosuppressive protocol after lung transplantation. *Transplantation.* 1999;68(1):67-71.
22. Slebos DJ, Kauffman HF, Koëter GH, Verschuuren EA, Bij W, Postma DS. Airway cellular response to two different immunosuppressive regimens in lung transplant recipients. *Clin Transplant.* 2005;19(2):243-249.

23. Combs MP, Walter K, Hixson H, et al. Impact of tacrolimus vs cyclosporine on chronic lung allograft dysfunction incidence and allograft survival in the International Society of Heart and Lung Transplantation registry. In: *J Heart Lung Transplant.* Vol 44. United States: Published by Elsevier Inc.; 2025:307-317.
24. Webster A, Woodroffe RC, Taylor RS, Chapman JR, Craig JC. Tacrolimus versus cyclosporin as primary immunosuppression for kidney transplant recipients. *Cochrane Database Syst Rev.* 2005(4):CD003961.
25. Haddad EM, McAlister VC, Renouf E, Malthaner R, Kjaer MS, Gluud LL. Cyclosporin versus tacrolimus for liver transplanted patients. *Cochrane Database Syst Rev.* 2006;2006(4):CD005161.
26. Penninga L, Møller CH, Gustafsson F, Steinbrüchel DA, Gluud C. Tacrolimus versus cyclosporine as primary immunosuppression after heart transplantation: systematic review with meta-analyses and trial sequential analyses of randomised trials. *Eur J Clin Pharmacol.* 2010;66(12):1177-1187.
27. Muduma G, Saunders R, Odeyemi I, Pollock RF. Systematic Review and Meta-Analysis of Tacrolimus versus Ciclosporin as Primary Immunosuppression After Liver Transplant. *PLoS One.* 2016;11(11):e0160421.
28. Yang K, Zhang M, Zhang B, Zhang Y, Zhao Q. Systematic review and meta-analysis of calcineurin inhibitors on long-term prognosis of renal transplant patients. In: *Transpl Immunol.* Vol 75. Netherlands: © 2022. Published by Elsevier B.V.; 2022:101741.
29. Liu JY, You RX, Guo M, et al. Tacrolimus Versus Cyclosporine as Primary Immunosuppressant After Renal Transplantation: A Meta-Analysis and Economics Evaluation. *Am J Ther.* 2016;23(3):e810-824.
30. Godinas L, Dobbels F, Hulst L, et al. Once daily tacrolimus conversion in lung transplantation: A prospective study on safety and medication adherence. In: *J Heart Lung Transplant.* Vol 40. United States: © 2021 International Society for Heart and Lung Transplantation. Published by Elsevier Inc; 2021:467-477.
31. Grootjans H, Verschuuren E, Kerstjens H, et al. Randomized Controlled Trial Comparing Immediate versus Extended Release Tacrolimus; Reducing Calcineurin Inhibitor Related Toxicity in Lung Transplantation Patients; The Revolution Trial. *The Journal of Heart and Lung Transplantation.* 2024;43(4):S36.
32. von Steinbüchel N, Limm H, Leopold C, Carr D. Assessment of health-related quality-of-life in patients after heart transplantation under therapy with tacrolimus or cyclosporine. *Transpl Int.* 2000;13 Suppl 1:S609-614.
33. Chambers DC, Cherikh WS, Harhay MO, et al. The International Thoracic Organ Transplant Registry of the International Society for Heart and Lung Transplantation: Thirty-sixth adult lung and heart-lung transplantation Report-2019; Focus theme: Donor and recipient size match. *J Heart Lung Transplant.* 2019;38(10):1042-1055.
34. Valapour M, Lehr CJ, Schladt DP, et al. OPTN/SRTR 2023 Annual Data Report: Lung. *Am J Transplant.* 2025;25(2s1):S422-s489.
35. McNeil K, Glanville AR, Wahlers T, et al. Comparison of mycophenolate mofetil and azathioprine for prevention of bronchiolitis obliterans syndrome in de novo lung transplant recipients. *Transplantation.* 2006;81(7):998-1003.
36. Palmer SM, Baz MA, Sanders L, et al. Results of a randomized, prospective, multicenter trial of mycophenolate mofetil versus azathioprine in the prevention of acute lung allograft rejection. *Transplantation.* 2001;71(12):1772-1776.
37. Bhorade SM, Jordan A, Villanueva J, et al. Comparison of three tacrolimus-based immunosuppressive regimens in lung transplantation. *Am J Transplant.* 2003;3(12):1570-1575.
38. Speich R, Schneider S, Hofer M, et al. Mycophenolate mofetil reduces alveolar inflammation, acute rejection and graft loss due to bronchiolitis obliterans syndrome after lung transplantation. *Pulm Pharmacol Ther.* 2010;23(5):445-449.
39. Tague LK, Byers DE, Hachem R, et al. Impact of SLCO1B3 polymorphisms on clinical outcomes in lung allograft recipients receiving mycophenolic acid. *Pharmacogenomics J.* 2020;20(1):69-79.
40. Sanborn DM, Friesen JN, Yadav H, et al. Outcomes in lung transplant recipients on azathioprine compared to mycophenolate. *Respir Med.* 2025;239:107991.
41. Ross DJ, Waters PF, Levine M, Kramer M, Ruzevich S, Kass RM. Mycophenolate mofetil versus azathioprine immunosuppressive regimens after lung transplantation: preliminary experience. *J Heart Lung Transplant.* 1998;17(8):768-774.
42. Betonico GN, Abudd-Filho M, Goloni-Bertollo EM, Pavarino-Bertelli E. Pharmacogenetics of mycophenolate mofetil: a promising different approach to tailoring immunosuppression? *J Nephrol.* 2008;21(4):503-509.
43. Nelson J, Alvey N, Bowman L, et al. Consensus recommendations for use of maintenance immunosuppression in solid organ transplantation: Endorsed by the American College of Clinical Pharmacy,

American Society of Transplantation, and the International Society for Heart and Lung Transplantation. *Pharmacotherapy.* 2022;42(8):599-633.
44. Eichelbaum M, Ingelman-Sundberg M, Evans WE. Pharmacogenomics and individualized drug therapy. *Annu Rev Med.* 2006;57:119-137.
45. Dean L. Azathioprine Therapy and TPMT and NUDT15 Genotype. In: Pratt VM, Scott SA, Pirmohamed M, Esquivel B, Kattman BL, Malheiro AJ, eds. *Medical Genetics Summaries.* Bethesda (MD): National Center for Biotechnology Information (US); 2012.
46. Grieves K, Keller BC, Waldman G, Clark JE. Impact of reduced mycophenolate exposure on chronic lung allograft dysfunction incidence after lung transplant. *JHLT Open.* 2024;6:100156.
47. Bergan S, Brunet M, Hesselink DA, et al. Personalized Therapy for Mycophenolate: Consensus Report by the International Association of Therapeutic Drug Monitoring and Clinical Toxicology. *Ther Drug Monit.* 2021;43(2):150-200.
48. Del Fante C, Scudeller L, Oggionni T, et al. Long-term off-line extracorporeal photochemotherapy in patients with chronic lung allograft rejection not responsive to conventional treatment: A 10-year single-centre analysis. *Respiration.* 2015;90(2):118-128.
49. Hage CA, Klesney-Tait J, Wille K, et al. Extracorporeal photopheresis to attenuate decline in lung function due to refractory obstructive allograft dysfunction. *Transfus Med.* 2021;31(4):292-302.
50. Jaksch P, Scheed A, Keplinger M, et al. A prospective interventional study on the use of extracorporeal photopheresis in patients with bronchiolitis obliterans syndrome after lung transplantation. *J Heart Lung Transplant.* 2012;31(9):950-957.
51. Leroux J, Hirschi S, Essaydi A, et al. Initiation of extracorporeal photopheresis in lung transplant patients with mild to moderate refractory BOS: A single-center real-life experience. *Respir Med Res.* 2022;81:100913.
52. Moniodis A, Townsend K, Rabin A, et al. Comparison of extracorporeal photopheresis and alemtuzumab for the treatment of chronic lung allograft dysfunction. *J Heart Lung Transplant.* 2018;37(3):340-348.
53. Pecoraro Y, Carillo C, Diso D, et al. Efficacy of Extracorporeal Photopheresis in Patients With Bronchiolitis Obliterans Syndrome After Lung Transplantation. *Transplant Proc.* 2017;49(4):695-698.
54. Salerno CT, Park SJ, Kreykes NS, et al. Adjuvant treatment of refractory lung transplant rejection with extracorporeal photopheresis. *J Thorac Cardiovasc Surg.* 1999;117(6):1063-1069.
55. Benden C, Speich R, Hofbauer GF, et al. Extracorporeal photopheresis after lung transplantation: a 10-year single-center experience. *Transplantation.* 2008;86(11):1625-1627.
56. Chionis L, Grossman BJ, Hachem R, et al. The efficacy of extracorporeal photopheresis to arrest bronchiolitis obliterans in lung allograft recipients was compared between two automated photopheresis instruments. *Transfusion.* 2018;58(12):2933-2941.
57. Greer M, Dierich M, De Wall C, et al. Phenotyping established chronic lung allograft dysfunction predicts extracorporeal photopheresis response in lung transplant patients. *Am J Transplant.* 2013;13(4):911-918.
58. Isenring B, Robinson C, Buergi U, et al. Lung transplant recipients on long-term extracorporeal photopheresis. *Clin Transplant.* 2017;31(10).
59. Karnes HE, Schindler EI, Morrell M, et al. Factors Associated With Mortality and Response to Extracorporeal Photopheresis in Lung Allograft Recipients With Bronchiolitis Obliterans Syndrome. *Transplantation.* 2019;103(5):1036-1042.
60. Meloni F, Cascina A, Miserere S, Perotti C, Vitulo P, Fietta AM. Peripheral CD4(+)CD25(+) TREG cell counts and the response to extracorporeal photopheresis in lung transplant recipients. *Transplant Proc.* 2007;39(1):213-217.
61. Morrell MR, Despotis GJ, Lublin DM, Patterson GA, Trulock EP, Hachem RR. The efficacy of photopheresis for bronchiolitis obliterans syndrome after lung transplantation. *J Heart Lung Transplant.* 2010;29(4):424-431.
62. Vazirani J, Routledge D, Snell GI, et al. Outcomes Following Extracorporeal Photopheresis for Chronic Lung Allograft Dysfunction Following Lung Transplantation: A Single-Center Experience. *Transplant Proc.* 2021;53(1):296-302.
63. O'Hagan AR, Stillwell PC, Arroliga A, Koo A. Photopheresis in the treatment of refractory bronchiolitis obliterans complicating lung transplantation. Chest. 1999 May;115(5):1459-62.
64. Gautschi F, Vogelmann T, Ortmanns G, et al. Early extracorporeal photopheresis treatment is associated with better survival in patients with chronic or recurrent acute lung allograft dysfunction. J Clin Apher. 2024 Jun;39(3):e22128.

65. Greer M, Liu B, Magnusson JM, et al. Assessing treatment outcomes in CLAD: The Hannover-extracorporeal photopheresis model. *J Heart Lung Transplant.* 2023;42(2):209-217.
66. Benazzo A, Bagnera C, Ius F, et al. A European Multi-Center Analysis of Extracorporeal Photopheresis as Therapy for Chronic Lung Allograft Dysfunction. *Transpl Int.* 2023;36:11551.
67. Villanueva J, Bhorade SM, Robinson JA, Husain AN, Garrity ER, Jr. Extracorporeal photopheresis for the treatment of lung allograft rejection. *Ann Transplant.* 2000;5(3):44-47.
68. Benazzo A, Cho A, Auner S, et al. Extracorporeal photopheresis for the prevention of rejection after lung transplantation: a prospective randomised controlled trial. *Eur Respir J.* 2025;65(2).
69. Robinson CA, Huber L, Murer C, et al. Cessation of extracorporeal photopheresis in chronic lung allograft dysfunction: effects on clinical outcome in adults. *Swiss Med Wkly.* 2017;147:w14429.
70. Alemanno S, Jaksch P, Benazzo A. Extracorporeal Photopheresis in Lung Transplantation: Present Applications and Emerging Research. *Transplant Direct.* 2025;11(9):e1831.
71. Izhakian S, Wasser WG, Fox BD, Vainshelboim B, Reznik JE, Kramer MR. Effectiveness of Rabbit Antithymocyte Globulin in Chronic Lung Allograft Dysfunction. *Transplant Proc.* 2016;48(6):2152-2156.
72. January SE, Fester KA, Bain KB, et al. Rabbit antithymocyte globulin for the treatment of chronic lung allograft dysfunction. *Clin Transplant.* 2019;33(10):e13708.
73. Kotecha S, Paul E, Ivulich S, et al. Outcomes Following ATG Therapy for Chronic Lung Allograft Dysfunction. *Transplant Direct.* 2021;7(4):e681.
74. Margallo Iribarnegaray J, De Pablo Gafas A, Alonso Moralejo R, et al. Antithymocyte Globulin Treatment for Chronic Lung Allograft Dysfunction in Lung Transplant Recipients: Experience From a National Reference Transplant Center. *Transplant Proc.* 2021;53(9):2710-2717.
75. Snell GI, Esmore DS, Williams TJ. Cytolytic therapy for the bronchiolitis obliterans syndrome complicating lung transplantation. *Chest.* 1996;109(4):874-878.
76. Date H, Lynch JP, Sundaresan S, Patterson GA, Trulock EP. The impact of cytolytic therapy on bronchiolitis obliterans syndrome. *J Heart Lung Transplant.* 1998;17(9):869-875.
77. Dunn CT, Brandon W, Jacob A, et al. Anti-Thymocyte Globulin as a Therapy for Chronic Lung Allograft Dysfunction: A Single-Center Experience. *Clin Transplant.* 2024;38(10):e15461.
78. Kesten S, Rajagopalan N, Maurer J. Cytolytic therapy for the treatment of bronchiolitis obliterans syndrome following lung transplantation. *Transplantation.* 1996;61(3):427-430.
79. Padhye AA, Guffey D, Leon-Pena A, et al. Antithymocyte globulin therapy in chronic lung allograft dysfunction. *Front Transplant.* 2025;4:1607678.
80. Penninga L, Møller CH, Penninga EI, Iversen M, Gluud C, Steinbrüchel DA. Antibody induction therapy for lung transplant recipients. *Cochrane Database Syst Rev.* 2013;2013(11):Cd008927.
81. Hertig A, Zuckermann A. Rabbit antithymocyte globulin induction and risk of post-transplant lymphoproliferative disease in adult and pediatric solid organ transplantation: An update. In: *Transpl Immunol.* Vol 32. Netherlands: © 2015. Published by Elsevier B.V.; 2015:179-187.
82. van der Zwan M, Clahsen-Van Groningen MC, van den Hoogen MWF, et al. Comparison of Alemtuzumab and Anti-thymocyte Globulin Treatment for Acute Kidney Allograft Rejection. *Front Immunol.* 2020;11:1332.
83. Li KHC, Ho JCS, Recaldin B, et al. Acute Cellular Rejection and Infection Rates in Alemtuzumab vs Traditional Induction Therapy Agents for Lung and Heart Transplantation: A Systematic Review and Meta-analysis. *Transplant Proc.* 2018;50(10):3723-3731.
84. Varga AN, Johnson D, Sawinski DL, et al. Safety and Feasibility of Outpatient Rabbit Antithymocyte Globulin Induction Therapy Administration in Kidney Transplant Recipients. *Pharmacotherapy.* 2018;38(6):620-627.
85. Ensor CR, Rihtarchik LC, Morrell MR, et al. Rescue alemtuzumab for refractory acute cellular rejection and bronchiolitis obliterans syndrome after lung transplantation. *Clin Transplant.* 2017;31(4).
86. Reams BD, Musselwhite LW, Zaas DW, et al. Alemtuzumab in the treatment of refractory acute rejection and bronchiolitis obliterans syndrome after human lung transplantation. *Am J Transplant.* 2007;7(12):2802-2808.
87. van Vugt LK, van der Zwan M, Clahsen-van Groningen MC, et al. A Decade of Experience With Alemtuzumab Therapy for Severe or Glucocorticoid-Resistant Kidney Transplant Rejection. *Transpl Int.* 2023;36:11834.
88. van Vugt LK, Tegzess E, van der Zwan M, et al. Multicenter, Real-World Clinical Evaluation of Alemtuzumab and Anti-Thymocyte Globulin for Severe Acute T Cell-Mediated Kidney Transplant Rejection. *Clin Transplant.* 2024;38(12):e70046.

89. Benazzo A, Auner S, Boehm PM, et al. Outcomes with alemtuzumab induction therapy in lung transplantation: a comprehensive large-scale single-center analysis. *Transpl Int.* 2021;34(12):2633-2643.
90. da Fonseca AR, Justino CC, Campos de Molla V, et al. Outpatient subcutaneous alemtuzumab is feasible and safe for aplastic anemia and is associated with high response rates. *Blood Adv.* 2025;9(4):906-912.
91. Chukwu CA, Spiers HVM, Middleton R, et al. Alemtuzumab in renal transplantation. Reviews of literature and usage in the United Kingdom. *Transplant Rev (Orlando).* 2022;36(2):100686.
92. Aris RM, Walsh S, Chalermskulrat W, Hathwar V, Neuringer IP. Growth factor upregulation during obliterative bronchiolitis in the mouse model. *Am J Respir Crit Care Med.* 2002;166(3):417-422.
93. Hertz MI, Henke CA, Nakhleh RE, et al. Obliterative bronchiolitis after lung transplantation: a fibroproliferative disorder associated with platelet-derived growth factor. *Proc Natl Acad Sci U S A.* 1992;89(21):10385-10389.
94. Krebs R, Tikkanen JM, Ropponen JO, et al. Critical role of VEGF-C/VEGFR-3 signaling in innate and adaptive immune responses in experimental obliterative bronchiolitis. *Am J Pathol.* 2012;181(5):1607-1620.
95. Kallio EA, Koskinen PK, Aavik E, Buchdunger E, Lemström KB. Role of platelet-derived growth factor in obliterative bronchiolitis (chronic rejection) in the rat. *Am J Respir Crit Care Med.* 1999;160(4):1324-1332.
96. Bos S, De Sadeleer LJ, Vanstapel A, et al. Antifibrotic drugs in lung transplantation and chronic lung allograft dysfunction: a review. *Eur Respir Rev.* 2021;30(160).
97. Combs MP, Belloli EA, Gargurevich N, et al. Results from randomized trial of pirfenidone in patients with chronic rejection (STOP-CLAD study). *J Heart Lung Transplant.* 2024;43(9):1468-1477.
98. Perch M, Corris P, Lordan J, et al. A European multi-center, randomized controlled trial of Pirfenidone in bronchiolitis obliterans syndrome after bilateral lung transplantation. *Eur Respir J.* 2025.
99. Noble PW, Albera C, Bradford WZ, et al. Pirfenidone in patients with idiopathic pulmonary fibrosis (CAPACITY): two randomised trials. *Lancet.* 2011;377(9779):1760-1769.
100. King TE, Jr., Bradford WZ, Castro-Bernardini S, et al. A phase 3 trial of pirfenidone in patients with idiopathic pulmonary fibrosis. *N Engl J Med.* 2014;370(22):2083-2092.
101. Vos R, Wuyts WA, Gheysens O, et al. Pirfenidone in restrictive allograft syndrome after lung transplantation: A case series. *Am J Transplant.* 2018;18(12):3045-3059.
102. Bennett D, Lanzarone N, Fossi A, et al. Pirfenidone in chronic lung allograft dysfunction: a single cohort study. *Panminerva Med.* 2020;62(3):143-149.
103. Richeldi L, Azuma A, Cottin V, et al. Nerandomilast in Patients with Idiopathic Pulmonary Fibrosis. *N Engl J Med.* 2025;392(22):2193-2202.
104. West A, Chaudhuri N, Barczyk A, et al. Inhaled pirfenidone solution (AP01) for IPF: a randomised, open-label, dose-response trial. In: *Thorax.* Vol 78. England: © Author(s) (or their employer(s)) 2023. No commercial re-use. See rights and permissions. Published by BMJ.; 2023:882-889.
105. Ruttens D, Verleden SE, Demeyer H, et al. Montelukast for bronchiolitis obliterans syndrome after lung transplantation: A randomized controlled trial. *PLoS One.* 2018;13(4):e0193564.
106. Verleden GM, Verleden SE, Vos R, et al. Montelukast for bronchiolitis obliterans syndrome after lung transplantation: a pilot study. *Transpl Int.* 2011;24(7):651-656.
107. Vos R, Eynde RV, Ruttens D, et al. Montelukast in chronic lung allograft dysfunction after lung transplantation. *J Heart Lung Transplant.* 2019;38(5):516-527.
108. Bos S, Murray J, Marchetti M, et al. ERS/EBMT clinical practice guidelines on treatment of pulmonary chronic graft-versus-host disease in adults. *Eur Respir J.* 2024;63(3).
109. Williams KM, Pavletic SZ, Lee SJ, et al. Prospective Phase II Trial of Montelukast to Treat Bronchiolitis Obliterans Syndrome after Hematopoietic Cell Transplantation and Investigation into Bronchiolitis Obliterans Syndrome Pathogenesis. *Transplant Cell Ther.* 2022;28(5):264.e261-264.e269.
110. Clarridge K, Chin S, Eworuke E, Seymour S. A Boxed Warning for Montelukast: The FDA Perspective. *J Allergy Clin Immunol Pract.* 2021;9(7):2638-2641.
